# Meat consumption and risk of 25 common conditions: outcome-wide analyses in 475,000 men and women in the UK Biobank study

**DOI:** 10.1186/s12916-021-01922-9

**Published:** 2021-03-02

**Authors:** Keren Papier, Georgina K. Fensom, Anika Knuppel, Paul N. Appleby, Tammy Y. N. Tong, Julie A. Schmidt, Ruth C. Travis, Timothy J. Key, Aurora Perez-Cornago

**Affiliations:** 1 Cancer Epidemiology Unit, Nuffield Department of Population Health, University of Oxford, Richard Doll Building, Old Road Campus, Oxford, OX3 7LF UK; 2 Department of International Development, University of Oxford, 3 Mansfield Rd, Oxford, OX1 3TB UK

**Keywords:** Red meat, Processed meat, Poultry, Prospective cohort study, UK Biobank, Risk, Outcome-wide

## Abstract

**Background:**

There is limited prospective evidence on the association between meat consumption and many common, non-cancerous health outcomes. We examined associations of meat intake with risk of 25 common conditions (other than cancer).

**Methods:**

We used data from 474,985 middle-aged adults recruited into the UK Biobank study between 2006 and 2010 and followed up until 2017 (mean follow-up 8.0 years) with available information on meat intake at baseline (collected via touchscreen questionnaire), and linked hospital admissions and mortality data. For a large sub-sample (~ 69,000), dietary intakes were re-measured three or more times using an online, 24-h recall questionnaire.

**Results:**

On average, participants who reported consuming meat regularly (three or more times per week) had more adverse health behaviours and characteristics than participants who consumed meat less regularly, and most of the positive associations observed for meat consumption and health risks were substantially attenuated after adjustment for body mass index (BMI). In multi-variable adjusted (including BMI) Cox regression models corrected for multiple testing, higher consumption of unprocessed red and processed meat combined was associated with higher risks of ischaemic heart disease (hazard ratio (HRs) per 70 g/day higher intake 1.15, 95% confidence intervals (CIs) 1.07–1.23), pneumonia (1.31, 1.18–1.44), diverticular disease (1.19, 1.11–1.28), colon polyps (1.10, 1.06–1.15), and diabetes (1.30, 1.20–1.42); results were similar for unprocessed red meat and processed meat intakes separately. Higher consumption of unprocessed red meat alone was associated with a lower risk of iron deficiency anaemia (IDA: HR per 50 g/day higher intake 0.80, 95% CIs 0.72–0.90). Higher poultry meat intake was associated with higher risks of gastro-oesophageal reflux disease (HR per 30 g/day higher intake 1.17, 95% CIs 1.09–1.26), gastritis and duodenitis (1.12, 1.05–1.18), diverticular disease (1.10, 1.04–1.17), gallbladder disease (1.11, 1.04–1.19), and diabetes (1.14, 1.07–1.21), and a lower IDA risk (0.83, 0.76–0.90).

**Conclusions:**

Higher unprocessed red meat, processed meat, and poultry meat consumption was associated with higher risks of several common conditions; higher BMI accounted for a substantial proportion of these increased risks suggesting that residual confounding or mediation by adiposity might account for some of these remaining associations. Higher unprocessed red meat and poultry meat consumption was associated with lower IDA risk.

**Supplementary Information:**

The online version contains supplementary material available at 10.1186/s12916-021-01922-9.

## Background

The World Health Organization [1] and many national dietary advice bodies (e.g. the UK dietary guidelines [2]) have in recent years recommended a reduction of red and processed meat consumption, based on consistent evidence linking high processed meat, and probably red meat consumption, with colorectal cancer risk [1]. While the association between meat intake and cancer risk has been comprehensively studied [3, 4], there is less information on the association between meat consumption, especially poultry meat, and incidence of major non-cancerous health outcomes [5]. Although several prospective studies have assessed the association of unprocessed red meat and processed meat consumption with risk of cardiovascular disease [6] and diabetes [7], the evidence is equivocal for ischaemic heart disease [8–10] and limited for stroke subtypes (e.g. haemorrhagic stroke [11]). Moreover, the evidence on poultry and CVD is particularly limited [12], while the evidence on poultry and diabetes is unclear [13, 14]. This lack of clear and available evidence for major non-cancerous health outcomes might relate to outcome selection bias (i.e. only reporting the outcomes that are found to be statistically significant [15]), differences in the definition of outcomes and exposures, sample size, control of confounders, and/or length of follow-up used among different studies. Examining the association between meat consumption and multiple non-cancerous health outcomes in the same large cohort may help to clarify these associations [16].

This study uses an outcome-wide approach to prospectively examine associations of meat consumption with risk of 25 common conditions identified as the 25 leading causes of hospital admission (other than cancer) in a large UK cohort.

## Methods

### Study population

We used data from the UK Biobank study, a cohort of 503,317 men and women from across the UK [17]. Potential participants were recruited through the National Health Service (NHS) Patient Registers and invited to attend one of the 22 assessment centres between 2006 and 2010. Participants joining the study completed a baseline touchscreen questionnaire, provided anthropometric and biological data, and gave informed consent for their health to be followed up through linkage to electronic medical records.

### Assessment of dietary intake

Dietary intake was assessed using a touchscreen dietary questionnaire administered to all participants at baseline that included 29 questions on diet, assessing the consumption frequency of each listed food. Responses to the five questions on meat (unprocessed beef, unprocessed lamb/mutton, unprocessed pork, unprocessed poultry, and processed meat) were assigned values for frequency per week (never = 0, less than once per week = 0.5, once per week = 1, 2–4 times per week = 3, 5–6 times per week = 5.5, and once or more a day = 7). We then collapsed these meat intake frequencies into three or four categories to create approximately equal-sized groups (see Additional file 1: Methods 1 for additional detail).

Participants recruited after 2009, as well as participants who provided UK Biobank with an email address and agreed to be re-contacted, were additionally invited to complete the Oxford WebQ [18], an online 24-h recall questionnaire. Participants were asked to select how many portions of each food item they consumed over the previous 24 h, enabling calculation of mean grams per day by multiplying frequencies of consumption by standard portion sizes. Similar foods were then grouped together into meat types to match the touchscreen dietary questionnaire. We then assigned the mean WebQ meat intakes in participants who had completed at least three WebQs to each touchscreen meat category defined for all participants. Using these assigned means, we calculated trends in risk across categories of baseline meat intakes [4, 19]. This approach uses repeat measurements to estimate usual mean meat intakes in each category of meat intake, thereby reducing random error in the assessment of usual meat consumption (see Additional file 1: Methods 1 for additional detail).

### Assessment of health outcomes

The outcomes of interest in this study were incident cases of 25 common conditions. The conditions selected were those identified as the 25 leading, well-defined causes of non-cancerous hospital admission in this cohort based on the primary International Classification of Diseases (ICD) 10 diagnosis codes recorded during admission. Some of the commonest causes of hospital admission in this cohort (e.g. nausea or heartburn) were not considered to be separate conditions, because they were not well-defined and/or were likely to be associated with a diverse range of underlying conditions. Moreover, although diabetes was not among the 25 most common primary diagnoses associated with admission, it is a common secondary reason for admission and therefore *any* diagnosis of diabetes was included among the 25 common conditions examined (see Additional file 1: Table 1 for selected conditions and relevant diagnosis, and procedure codes).

**Table 1:** Baseline characteristics of participants by unprocessed red and processed meat intake in UK Biobank (*n* = 467,741, see Additional file 1: Fig. 1)

Characteristic	0–1 time/week	2 times/week	3–4 times/week	> 5 times/week
Mean (SD) or *n* (%)	*N* = 44,019	*N* = 160,069	*N* = 140,674	*N* = 122,979
**Sociodemographic**				
Sex, *n* (%)				
Women	31,318 (71.1)	101,747 (63.6)	71,679 (51.0)	47,657 (38.8)
Men	12,701 (28.9)	58,322 (36.4)	68,995 (49.0)	75,322 (61.2)
Age (years), mean (SD)	54.9 (8.2)	56.5 (8.0)	56.4 (8.1)	56.5 (8.2)
Race, *n* (%)				
White	38,451 (87.4)	151,727 (94.8)	134,509 (95.6)	116,815 (95.0)
Asian or Asian British	3468 (7.9)	2914 (1.8)	2245 (1.6)	1897 (1.5)
Black or Black British	886 (2.0)	2607 (1.6)	1685 (1.2)	2047 (1.7)
Mixed race/others	997 (2.3)	2323 (1.5)	1807 (1.3)	1774 (1.4)
Unknown	217 (0.5)	498 (0.3)	428 (0.3)	446 (0.4)
Townsend deprivation, *n* (%)				
Most affluent (mean − 4.7)	7036 (16.0)	32,846 (20.5)	29,803 (21.2)	24,666 (20.1)
2 (mean − 3.3)	7524 (17.1)	32,745 (20.5)	29,079 (20.7)	24,304 (19.8)
3 (mean − 2.1)	8341 (18.9)	32,737 (20.5)	28,324 (20.1)	24,343 (19.8)
4 (mean − 0.1)	10,146 (23.0)	31,753 (19.8)	27,446 (19.5)	24,066 (19.6)
Most deprived (mean 3.8)	10,910 (24.8)	29,773 (18.6)	25,869 (18.4)	25,448 (20.7)
Unknown	62 (0.1)	215 (0.1)	153 (0.1)	152 (0.1)
Qualification, *n* (%)				
College/university degree/NVQ	28,490 (64.7)	96,075 (60.0)	82,364 (58.5)	72,437 (58.9)
National examination at ages 17–18	2491 (5.7)	8708 (5.4)	7795 (5.5)	6626 (5.4)
National examination at age 16	6321 (14.4)	27,682 (17.3)	24,133 (17.2)	19,675 (16.0)
Others/unknown	6717 (15.3)	27,604 (17.2)	26,382 (18.8)	24,241 (19.7)
Employment, *n* (%)				
In paid employment	27,650 (62.8)	93,965 (58.7)	81,641 (58.0)	70,102 (57.0)
Pension	10,229 (23.2)	48,219 (30.1)	42,698 (30.4)	37,181 (30.2)
Not in paid employment	5556 (12.6)	16,487 (10.3)	15,236 (10.8)	14,560 (11.8)
Unknown	584 (1.3)	1398 (0.9)	1099 (0.8)	1136 (0.9)
**Physical measurements**				
BMI (kg/m^2^), mean (SD)	25.9 (4.7)	27.1 (4.7)	27.6 (4.8)	28.1 (4.9)
**Lifestyle**				
Smoking, *n* (%)				
Never	26,347 (59.9)	90,448 (56.5)	76,682 (54.5)	62,688 (51.0)
Former	13,962 (31.7)	54,796 (34.2)	48,534 (34.5)	43,268 (35.2)
Current < 15 cigarettes/day	1297 (2.9)	4581 (2.9)	4247 (3.0)	4074 (3.3)
Current ≥ 15 cigarettes/day	1030 (2.3)	4825 (3.0)	6015 (4.3)	7622 (6.2)
Current, amount unknown	1213 (2.8)	4876 (3.0)	4727 (3.4)	4914 (4.0)
Unknown	170 (0.4)	543 (0.3)	469 (0.3)	413 (0.3)
Physical activity level, *n* (%)				
Low < 10 excess METs	12,405 (28.2)	49,709 (31.1)	45,467 (32.3)	39,706 (32.3)
Moderate 10 to < 50 excess METs	22,380 (50.8)	80,039 (50.0)	68,196 (48.5)	58,006 (47.2)
High ≥ 50 excess METs	7769 (17.6)	24,715 (15.4)	21,860 (15.5)	20,458 (16.6)
Unknown	1465 (3.3)	5606 (3.5)	5151 (3.7)	4809 (3.9)
Alcohol intake, *n* (%)				
Non-drinkers	7503 (17.0)	11,938 (7.5)	9611 (6.8)	7790 (6.3)
< 1 g/day	6814 (15.5)	19,866 (12.4)	14,469 (10.3)	10,861 (8.8)
1 to < 10 g/day	14,742 (33.5)	57,211 (35.7)	43,226 (30.7)	31,588 (25.7)
10 to < 20 g/day	7984 (18.1)	36,199 (22.6)	31,681 (22.5)	25,627 (20.8)
20+ g/day	6740 (15.3)	34,045 (21.3)	41,068 (29.2)	46,544 (37.8)
Unknown	236 (0.5)	810 (0.5)	619 (0.4)	569 (0.5)
**Diet**				
Fruit and vegetable intake (s/day), mean (SD)	5.59 (3.19)	4.89 (2.54)	4.50 (2.45)	4.33 (2.50)
Cereal fibre intake (g/day), mean (SD)	4.66 (3.11)	4.52 (2.89)	4.52 (2.91)	4.44 (2.96)
Oily fish, *n* (%)				
0 time/week	12,568 (28.6)	12,192 (7.6)	13,264 (9.4)	13,296 (10.8)
< 1 time/week	8444 (19.2)	52,209 (32.6)	49,864 (35.4)	44,506 (36.2)
1 time/week	11,522 (26.2)	62,503 (39.0)	55,502 (39.5)	46,265 (37.6)
> 2 times/week	11,299 (25.7)	32,572 (20.3)	21,480 (15.3)	18,279 (14.9)
Unknown	186 (0.4)	593 (0.4)	564 (0.4)	633 (0.5)
Non-oily fish, *n* (%)				
< 1 time/week	19,962 (45.3)	54,020 (33.7)	45,185 (32.1)	38,703 (31.5)
1 time/week	14,427 (32.8)	79,177 (49.5)	74,260 (52.8)	63,929 (52.0)
> 2 times/week	9432 (21.4)	26,393 (16.5)	20,801 (14.8)	19,887 (16.2)
Unknown	198 (0.4)	479 (0.3)	428 (0.3)	460 (0.4)
Poultry meat, *n* (%)				
0–1 time/week	26,359 (59.9)	23,487 (14.7)	13,477 (9.6)	10,760 (8.7)
2 times/week	7141 (16.2)	61,114 (38.2)	56,696 (40.3)	42,291 (34.4)
> 3 times/week	10,461 (23.8)	75,358 (47.1)	70,405 (50.0)	69,835 (56.8)
Unknown	58 (0.1)	110 (0.1)	96 (0.1)	93 (0.1)
**Women factors**				
Menopausal status, *n* (%)				
Premenopausal	8960 (28.6)	22,946 (22.6)	17,038 (23.8)	11,207 (23.5)
Postmenopausal	20,588 (65.7)	73,267 (72.0)	50,660 (70.7)	33,725 (70.8)
Unknown	1770 (5.7)	5534 (5.4)	3981 (5.6)	2725 (5.7)
Parity, *n* (%)				
0 births	8314 (26.5)	19,781 (19.4)	11,737 (16.4)	7298 (15.3)
1–2 births	16,231 (51.8)	58,204 (57.2)	42,076 (58.7)	27,654 (58.0)
≥ 3 births	6717 (21.4)	23,671 (23.3)	17,812 (24.8)	12,655 (26.6)
Unknown	56 (0.2)	91 (0.1)	54 (0.1)	50 (0.1)
HRT use, *n* (%)				
Never	21,436 (68.4)	61,911 (60.8)	44,006 (61.4)	28,975 (60.8)
Past	7951 (25.4)	33,162 (32.6)	23,018 (32.1)	15,560 (32.6)
Current	1773 (5.7)	6379 (6.3)	4429 (6.2)	2897 (6.1)
Unknown	158 (0.5)	295 (0.3)	226 (0.3)	225 (0.5)
OCP use, *n* (%)				
Never	6596 (21.1)	18,180 (17.9)	12,849 (17.9)	8917 (18.7)
Past	23,874 (76.2)	81,528 (80.1)	57,266 (79.9)	37,642 (79.0)
Current	691 (2.2)	1812 (1.8)	1388 (1.9)	918 (1.9)
Unknown	157 (0.5)	227 (0.2)	176 (0.2)	180 (0.4)

The *x*^2^ test was used to compare the distribution between meat intakes for all categorical variables. Analysis of variance (ANOVA) was used to compare the means between meat intakes. The *P* heterogeneity between meat intakes was < 0.001 for all variables. All dietary data come from the touchscreen questionnaire. *BMI* body mass index, *HRT* hormone replacement therapy, *OCP* oral contraceptive pill use, *NVQ* national vocational qualification, *s/day* servings/day, *g/day* grams/day

Participant information on cause-specific in-patient hospital admissions and deaths (primary cause for all outcomes except diabetes which also included any diagnosis for hospital admission or mention on the death certificate) was obtained through linkage to the NHS Central Registers. For participants in England, Hospital Episode Statistics (HES) and information on date and cause of death were available until the 31st of March 2017; for participants in Scotland, Scottish Morbidity Records and information on date and cause of death were available until the 31st of October 2016; and for participants in Wales, the Patient Episode Database and information on date and cause of death were available until the 29th of February 2016. We also obtained information on cancer registrations (including date and cancer site) from the NHS Central Registers (see Additional file 1: Methods 2 and Additional file 1: Table 1 for information on exclusion, diagnosis and procedure codes).

### Exclusions

Of the 503,317 recruited participants, 28,332 were excluded due to study withdrawals, prevalent cancer (except non-melanoma skin cancer, ICD-10 C44), or because their genetic sex differed from their reported gender, resulting in a maximal study sample of 474,985 (94%). Participants with a relevant diagnosis or procedure prior to recruitment, ascertained through the touchscreen questionnaire, nurse-guided interviews, and hospital admission data, were excluded for each condition (see Additional file 1: Table 1 for details about the exclusions for each outcome). Participants who did not report their meat intake in the touchscreen questionnaire or reported ‘prefer not to say’ or ‘do not know’ were classified as missing and excluded for the respective exposure analyses (see Additional file 1: Fig. 1 for participant flowchart and Additional file 1: Tables 6, 7, 8, 9, and 10 for total numbers for each exposure and outcome).

### Statistical analysis

**Fig. 1:**
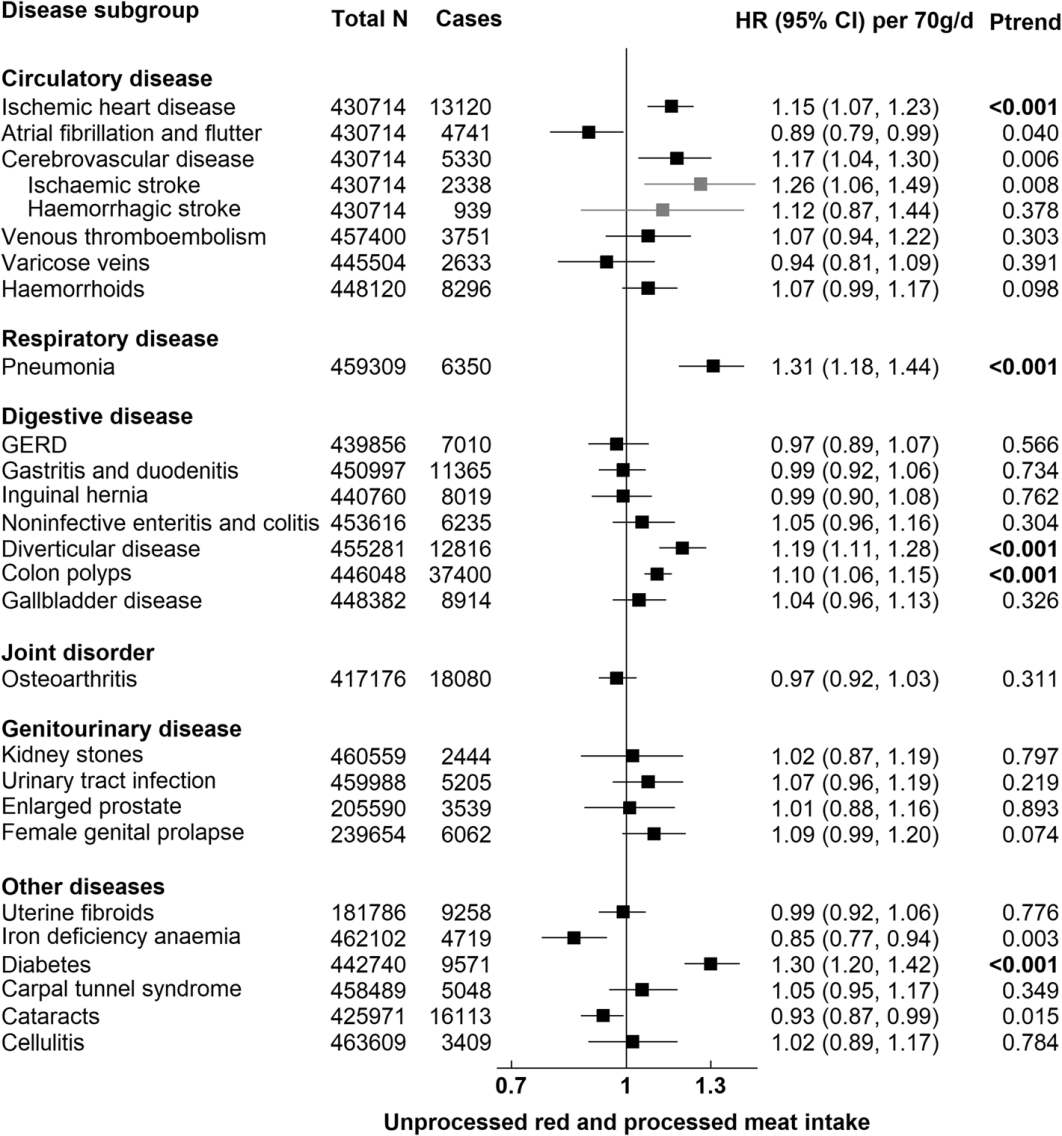
Risk of 25 common conditions per 70 grams/day (g/d) higher daily intake of unprocessed red and processed meat. Stratified for sex, age group, and region and adjusted for age (underlying time variable), race (4 groups where possible: White, Asian or Asian British, Black or Black British, mixed race or other, unknown), deprivation (Townsend index quintiles, unknown), qualification (college or university degree/vocational qualification, national examination at ages 17–18, national examination at age 16, others/unknown), employment (in paid employment, receiving pension, not in paid employment, unknown), smoking (never, former, current < 15 cigarettes/day, current > 15 cigarettes/day, current unknown amount of cigarettes/day, unknown), physical activity (< 10 excess METs per/week, 10 to < 50 excess METs per/week, ≥ 50 excess METs per/week, unknown), alcohol intake (none, < 1 g/day, 1 to < 10 g/day, 10 to < 20 g/day, ≥ 20 g/day, unknown), total fruit and vegetable intake (< 3 servings/day, 3 to < 4 servings/day, 4 to < 6 servings/day, ≥ 6 servings/day, unknown), cereal fibre score (sex-specific quintiles, unknown), oily fish intake (0 time/week,< 1 time/week, 1 time/week, > 2 times/week, unknown), non-oily fish intake (< 1 time/week, 1 time/week, > 2 times/week, unknown), BMI (sex-specific quintiles, unknown), in women: menopausal status (pre-, postmenopausal, unknown), HRT (never, past, current, unknown), OCP use (never, past, current, unknown), and parity (nulliparous, 1–2, ≥ 3, unknown). BMI body mass index, HRT hormone replacement therapy, OCP oral contraceptive pill, GERD Gastro-oesophageal reflux disease. *P* trend in bold indicates *P* value robust to Bonferroni correction (*P* < 0.002)

**Fig. 2:**
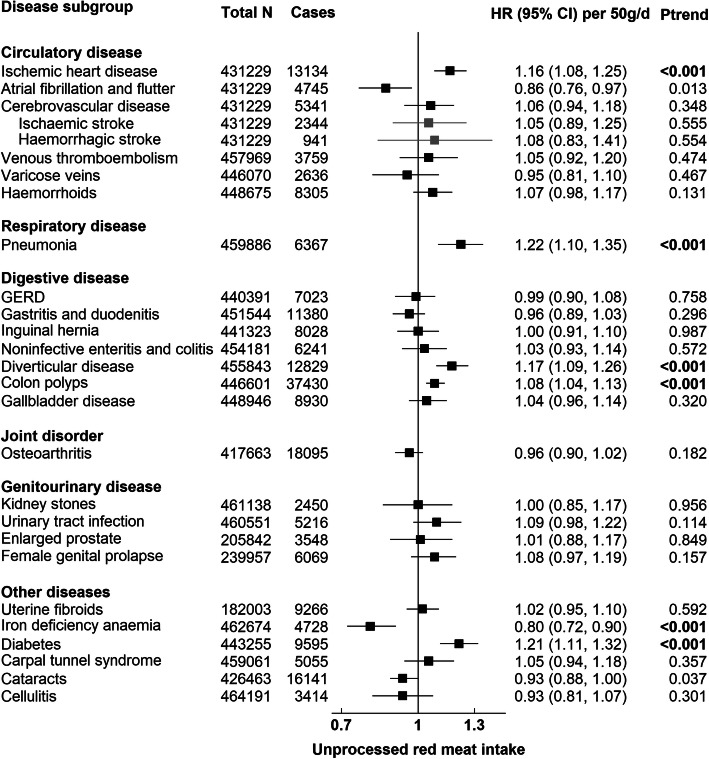
Risk of 25 common conditions per 50 grams/day (g/d) higher daily intake of unprocessed red meat. Stratified for sex, age group, and region and adjusted for age (underlying time variable), race (4 groups where possible: White, Asian or Asian British, Black or Black British, mixed race or others, unknown), deprivation (Townsend index quintiles, unknown), qualification (college or university degree/vocational qualification, national examination at ages 17–18, national examination at age 16, others/unknown), employment (in paid employment, receiving pension, not in paid employment, unknown), smoking (never, former, current < 15 cigarettes/day, current > 15 cigarettes/day, current unknown amount of cigarettes/day, unknown), physical activity (< 10 excess METs per/week, 10 to < 50 excess METs per/week, ≥ 50 excess METs per/week, unknown), alcohol intake (none, < 1 g/day, 1 to < 10 g/day, 10 to < 20 g/day, ≥ 20 g/day, unknown), total fruit and vegetable intake (< 3 servings/day, 3 to < 4 servings/day, 4 to < 6 servings/day, ≥ 6 servings/day, unknown), cereal fibre score (sex-specific quintiles, unknown), oily fish intake (0 time/week,< 1 time/week, 1 time/week, > 2 times/week, unknown), non-oily fish intake (< 1 time/week, 1 time/week, > 2 times/week, unknown), BMI (sex-specific quintiles, unknown), in women: menopausal status (pre-, postmenopausal, unknown), HRT (never, past, current, unknown), OCP use (never, past, current, unknown), and parity (nulliparous, 1–2, ≥ 3, unknown). BMI body mass index, HRT hormone replacement therapy, OCP oral contraceptive pill, GERD Gastro-oesophageal reflux disease. *P* trend in bold indicates *P* value robust to Bonferroni correction (*P* < 0.002)

**Fig. 3:**
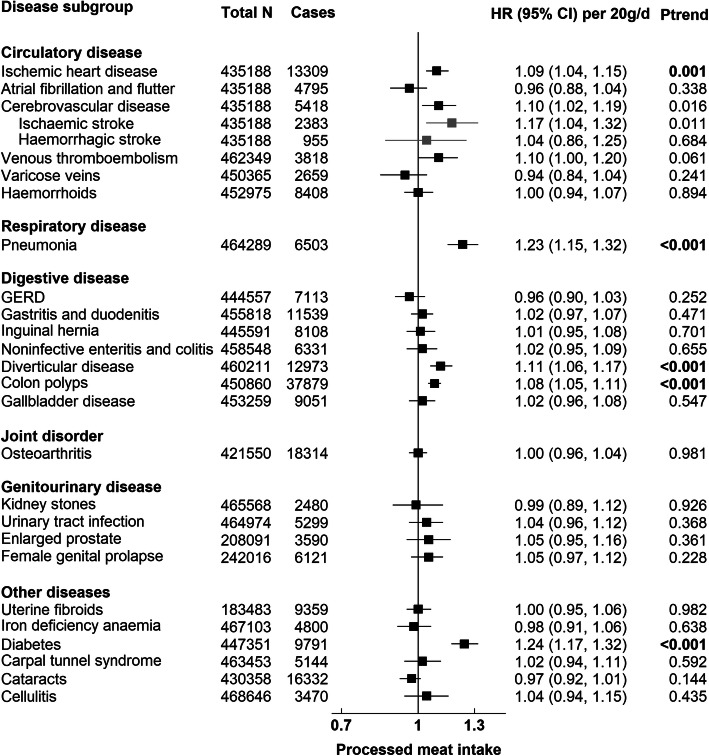
Risk of 25 common conditions per 20 grams/day (g/d) higher daily intake of processed meat. Stratified for sex, age group, and region and adjusted for age (underlying time variable), race (4 groups where possible: White, Asian or Asian British, Black or Black British, mixed race or others, unknown), deprivation (Townsend index quintiles, unknown), qualification (college or university degree/vocational qualification, national examination at ages 17–18, national examination at age 16, others/unknown), employment (in paid employment, receiving pension, not in paid employment, unknown), smoking (never, former, current < 15 cigarettes/day, current > 15 cigarettes/day, current unknown amount of cigarettes/day, unknown), physical activity (< 10 excess METs per/week, 10 to < 50 excess METs per/week, ≥ 50 excess METs per/week, unknown), alcohol intake (none, < 1 g/day, 1 to < 10 g/day, 10 to < 20 g/day, ≥ 20 g/day, unknown), total fruit and vegetable intake (< 3 servings/day, 3 to < 4 servings/day, 4 to < 6 servings/day, ≥ 6 servings/day, unknown), cereal fibre score (sex-specific quintiles, unknown), oily fish intake (0 time/week, < 1 time/week, 1 time/week, > 2 times/week, unknown), non-oily fish intake (< 1 time/week, 1 time/week, > 2 times/week, unknown), BMI (sex-specific quintiles, unknown), in women: menopausal status (pre-, postmenopausal, unknown), HRT (never, past, current, unknown), OCP use (never, past, current, unknown), and parity (nulliparous, 1–2, ≥ 3, unknown). BMI body mass index, HRT hormone replacement therapy, OCP oral contraceptive pill, GERD Gastro-oesophageal reflux disease. *P* trend in bold indicates *P* value robust to Bonferroni correction (*P* < 0.002)

**Fig. 4:**
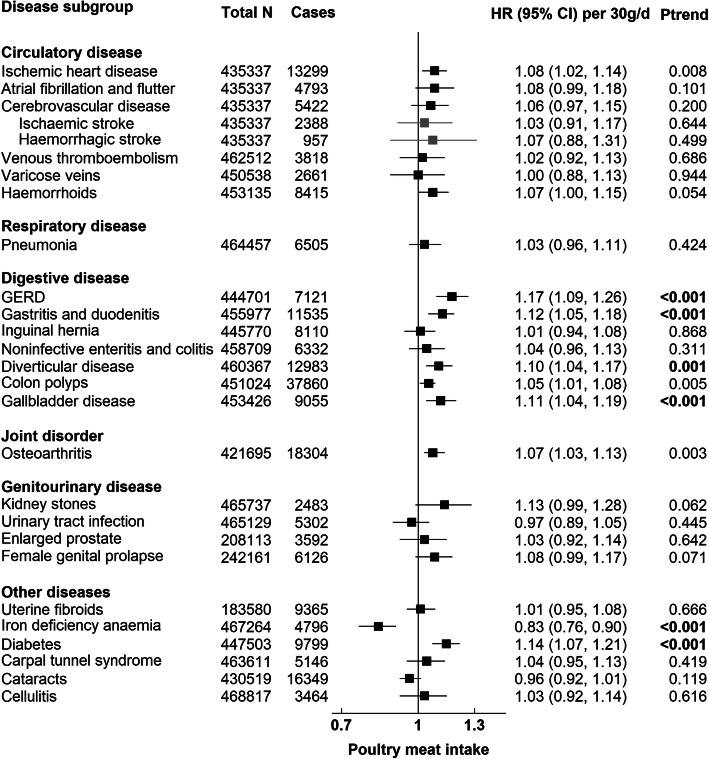
Risk of 25 common conditions per 30 grams/day (g/d) higher daily intake of poultry meat. Stratified for sex, age group, and region and adjusted for age (underlying time variable), race (4 groups where possible: White, Asian or Asian British, Black or Black British, mixed race or others, unknown), deprivation (Townsend index quintiles, unknown), qualification (college or university degree/vocational qualification, national examination at ages 17–18, national examination at age 16, other/unknown), employment (in paid employment, receiving pension, not in paid employment, unknown), smoking (never, former, current < 15 cigarettes/day, current > 15 cigarettes/day, current unknown amount of cigarettes/day, unknown), physical activity (< 10 excess METs per/week, 10 to < 50 excess METs per/week, ≥ 50 excess METs per/week, unknown), alcohol intake (none, < 1 g/day, 1 to < 10 g/day, 10 to < 20 g/day, ≥ 20 g/day, unknown), total fruit and vegetable intake (< 3 servings/day, 3 to < 4 servings/day, 4 to < 6 servings/day, ≥ 6 servings/day, unknown), cereal fibre score (sex-specific quintiles, unknown), oily fish intake (0 time/week, < 1 time/week, 1 time/week, > 2 times/week, unknown), non-oily fish intake (< 1 time/week, 1 time/week, > 2 times/week, unknown), BMI (sex-specific quintiles, unknown), in women: menopausal status (pre-, postmenopausal, unknown), HRT (never, past, current, unknown), OCP use (never, past, current, unknown), and parity (nulliparous, 1–2, ≥ 3, unknown). BMI body mass index, HRT hormone replacement therapy, OCP oral contraceptive pill, GERD Gastro-oesophageal reflux disease. *P* trend in bold indicates *P* value robust to Bonferroni correction (*P* < 0.002)

We used Cox proportional hazards regression models to assess associations between meat consumption and risk for incident cases separately for each disease or condition, calculating trends using the mean meat intakes calculated using the WebQ questionnaires for each category from the touchscreen questionnaire and the trend test variables. Participants’ survival time in person-years was calculated from their age at recruitment until their age at hospital admission, death, loss to follow-up, or administrative censoring. All analyses were stratified by sex, age at recruitment, and geographical region (Model 0). In Model 1, we estimated hazard ratios (HRs) and 95% confidence intervals (CIs) adjusted for race, Townsend deprivation index [20], education, employment, smoking, alcohol consumption, and physical activity, and in women, we additionally adjusted for menopausal status, hormone replacement therapy, oral contraceptive pill use, and parity. In Model 2, we further adjusted for total fruit and vegetable intake, cereal fibre intake score (calculated by multiplying the frequency of consumption of bread and breakfast cereal by the fibre content of these foods [21]), oily fish intake, and non-oily fish intake. For Model 3, we added adjustment for body mass index (BMI). Missing data for all covariates was minimal (< 10%) and thus a ‘missing’ category was created for each covariate (see Figs. 1, 2, 3, and 4 footnotes and Additional file 1: Methods 3 for full adjustment description with definitions of categories)

#### Sensitivity analyses

To examine whether the associations between meat intake and risk of incidence for specific diagnoses could be affected by reverse causality or residual confounding by smoking, we repeated the analyses (1) after excluding the first 4 years of follow-up and (2) restricted to never smokers.

All analyses were conducted using STATA version 15.1 (Stata Corp LP, College Station, TX). All *P* values were two-sided and Bonferroni correction was used to allow for multiple testing (for 25 outcomes, *P* < 0.002).

## Results

### Baseline characteristics

Table 1 shows baseline characteristics of participants by categories of unprocessed red meat and processed meat intake. Around one-third of participants consumed unprocessed red and/or processed meat once or more daily. On average, participants who consumed unprocessed red and processed meat regularly (three or more times per week) were more likely to be men, older, of White European race, retired, have higher BMI, smoke and consume alcohol, and consume less fruit and vegetables, fibre, and fish and more poultry meat; they were also less likely to have attained a tertiary education, and among women to have two or more children, not use oral contraceptives, use hormone replacement therapy, or be postmenopausal compared with participants who consumed meat less than three times per week (*P* < 0.001 for heterogeneity between meat intakes for all baseline characteristics). Participants who consumed higher amounts of unprocessed red meat were more likely to consume higher amounts of processed meat and poultry meat (see Additional file 1: Table 3). Baseline characteristics in relation to poultry meat consumption were somewhat different (see Additional file 1: Table 5).

### Risk analyses

Figures 1, 2, 3, and 4 present the numbers of incident cases for 25 common conditions and their HRs and 95% CIs per unit higher intake of meat for the multiple-adjusted model (Model 3) over an average follow-up of 8.0 years (standard deviation 1.0). Risks by categories of meat intake at baseline for Models 0–3 can be found in Additional file 1: Tables 6, 7, 8, 9, and 10. Overall, many of the positive associations were substantially attenuated, and in some cases were no longer statistically significant, with the additional adjustment for BMI (Model 3). Here we describe the results for Model 3 that were robust to correction for multiple testing. Risks for total meat intake (unprocessed red, processed, and poultry meat combined) did not yield any additional associations and these results are therefore only presented in Additional file 1: Table 6 and Fig. 7.

#### Total unprocessed red meat and processed meat

Total unprocessed red meat and processed meat intake was associated with a higher risk of ischaemic heart disease (IHD) (HR per 70 g/day higher intake = 1.15, 95% CI 1.07–1.23), pneumonia (1.31, 1.18–1.44), diverticular disease (1.19, 1.11–1.28), colon polyps (1.10, 1.06–1.15), and diabetes (1.30, 1.20–1.42) (Fig. 1).

#### Unprocessed red meat

Unprocessed red meat intake was associated with a higher risk of IHD (HR per 50 g/day higher intake = 1.16, 95% CI 1.08–1.25), pneumonia (1.22, 1.10–1.35), diverticular disease (1.17, 1.09–1.26), colon polyps (1.08, 1.04–1.13), and diabetes (1.21, 1.11–1.32), and a lower risk of IDA (0.80, 0.72–0.90) (Fig. 2).

#### Processed meat

Processed meat intake was associated with a higher risk of IHD (HR per 20 g/day higher intake = 1.09, 95% CI 1.04–1.15), pneumonia (1.23, 95% CI 1.15–1.32), diverticular disease (1.11, 1.06–1.17) colon polyps (1.08, 95% CI 1.05–1.11), and diabetes (1.24, 1.17–1.32) (Fig. 3).

#### Poultry meat

Poultry meat intake was associated with a higher risk of gastro-oesophageal reflux disease (GERD) (HR per 30 g/day higher intake = 1.17, 95% CI 1.09–1.26), gastritis and duodenitis (1.12, 1.05–1.18), diverticular disease (1.10, 1.04–1.17), gallbladder disease (1.11, 1.04–1.19), and diabetes (1.14, 1.07–1.21), and a lower risk of IDA (0.83, 0.76–0.90) (Fig. 4).

#### Sensitivity analysis

Associations were similar when excluding the first 4 years of follow-up and in never smokers (Additional file 1: Figs. 2, 3, 4, 5, and 6.). However, we did note a positive association between unprocessed red and processed meat intake (combined) and haemorrhagic stroke (HR per 70 g/day higher intake = 1.53, 95% CI 1.10–2.14) in participants diagnosed after 4 or more years of follow-up and that the associations between unprocessed red meat intake and diabetes risk, and processed meat intake and IHD risk, were no longer statistically significant in never smokers.

## Discussion

In this large, prospective cohort of nearly 0.5 million UK adults, we observed that after allowing for multiple testing, higher consumption of unprocessed red and processed meat combined was associated with higher risks of IHD, pneumonia, diverticular disease, colon polyps, and diabetes, and higher consumption of poultry meat was associated with higher risks of GERD, gastritis and duodenitis, diverticular disease, gallbladder disease, and diabetes. Differences in BMI across the categories of meat consumption appear to account for a substantial part of the increased risks, suggesting that residual confounding by adiposity may still operate. We also observed inverse associations between higher intakes of unprocessed red meat and poultry meat and IDA, which were minimally affected by adjustment for BMI.

### Circulatory diseases

Similar to our findings, a recent meta-analysis of prospective studies [6] and a recent prospective study from the Pan-European EPIC cohort which included over 7000 IHD cases [9] reported positive associations between unprocessed red meat and processed meat consumption and risk of IHD. For stroke, previous meta-analyses of prospective studies [22, 23] and a recent prospective study from the EPIC cohort [24] both reported null associations for unprocessed red and processed meat intake and haemorrhagic stroke; this is consistent with our main findings but not with our findings in participants diagnosed after 4 or more years of follow-up, although this might be a chance finding due to shorter follow-up. Processed meats contain high amounts of sodium [25], a risk factor for high blood pressure [26], which is a causal risk factor for IHD and stroke [27]. Furthermore, unprocessed red meat and processed meat are major dietary sources of saturated fatty acids (SFAs) which can increase low-density lipoprotein (LDL) cholesterol, an established causal risk factor for IHD [28]. It is also possible that the positive association we observed for unprocessed red meat intake and IHD risk might relate to gut microbiota metabolism, for example through the production of trimethylamine-*N*-oxide [25–27], but the importance of this potential pathway is uncertain.

### Respiratory disease

Higher consumption of unprocessed red and processed meat was associated with a higher risk of pneumonia. To the best of our knowledge, these associations have not been shown previously, except for one recent study that found that higher intake of red meat (both processed and unprocessed) was associated with a higher risk of death due to respiratory disease, which included pneumonia [21]. It is possible that the observed association might reflect a causal link, for example related to the high availability of iron in unprocessed red and processed meat (see further discussion below in relation to anaemia), since excess iron has been found to be associated with a higher risk of infection [29] and increased availability of iron for pathogens [30]. It is also possible that hospital admission for pneumonia is a marker for co-morbidity and overall frailty [31]; therefore, residual confounding might operate (see further discussion on residual confounding below).

### Digestive diseases

Few prospective studies have examined the risk for diverticular disease [32, 33], but consistent with our findings, the Health Professionals Follow-up Study (HPFS) observed increased risks of incident diverticulitis with higher consumption of unprocessed red and processed meat [32]. The HPFS did not observe an association for poultry meat, but had lower power than the current study. Meat consumption might affect the risk of diverticular disease via the intestinal microbiome, by altering microbial community structure and metabolism [34].

A recent meta-analysis of prospective studies reported that unprocessed red and processed meat consumption was positively associated with the risk of colorectal adenomas [35], which is consistent with our findings for colon polyps. Unprocessed red meat is a source of heme iron and processed meat usually contains nitrite and nitrates; these can increase the formation of *N*-nitroso compounds [36], which are mutagenic and have been associated with a higher risk of colorectal adenomas [37].

To our knowledge, this is the first prospective study of meat consumption and risk of GERD and gastritis and duodenitis. We found a positive association between poultry meat intake and GERD risk, whereas the available cross-sectional evidence suggests a null association for meat (total) [38–41]. We also found a positive association between poultry meat consumption and risk of gastritis and duodenitis. *Helicobacter pylori*, a bacterium that increases the risk of gastritis [42], has been previously detected in raw poultry meat [43]. Therefore, it is possible that the observed association might relate to inappropriate handling or cooking of poultry meat, but additional research is needed.

Some published studies have found evidence of an association between higher unprocessed red and processed meat consumption and gallbladder disease which remained after BMI adjustment [16, 17], whereas in our analyses this association was greatly attenuated and not significant after adjusting for BMI. In the present study, BMI was calculated from standardised measurements of weight and height, whereas previous studies used self-reported weight and height. Therefore, it is possible that adjusting for BMI in this study explained a larger proportion of the observed associations; high BMI has been consistently shown to be associated with a large increase in the risk of gallbladder disease in both observational and genetic studies [18–20]. We observed a novel association between poultry intake and gallbladder disease, though additional research is needed to assess this association.

### Other diseases

We found an inverse association between the consumption of unprocessed red meat and poultry meat and risk of IDA. Some previous evidence from prospective studies [44] supports these findings and has also shown a positive association between unprocessed red meat [45] and total meat [46–48] consumption and indicators of body iron stores. Moreover, previous cross-sectional work from the UK Biobank has shown that people who did not consume meat were more likely to be anaemic [49]. This association is likely related to the high availability of heme iron in meat, which is more easily absorbed than non-heme iron [50].

Similar to our findings, meta-analyses of prospective cohort studies have consistently reported a positive association between unprocessed red and processed meat consumption and risk of diabetes [7, 51, 52]. We also found a positive association between poultry meat consumption and risk of diabetes, which has been reported in some [14] but not all prospective studies [13, 53]. Obesity is the major risk factor for diabetes, and the association for unprocessed and processed meat intake (combined) and diabetes in the present study was substantially attenuated (by ~ 60%) after adjusting for BMI, suggesting that the remaining association with meat may be entirely due to higher adiposity. It is also possible that meat could affect risk independently of adiposity; for example, high intakes of heme iron and greater iron storage may promote the formation of hydroxyl radicals that damage the pancreatic beta cells, thereby impairing insulin synthesis and excretion [54, 55].

### Role of BMI

In the present study, most of the positive associations between meat consumption and health risks were substantially attenuated after adjusting for BMI, suggesting that BMI was a strong confounder or possible mediator for many of the meat and disease associations. BMI is an important risk factor for many of the diseases examined (e.g. diabetes [7]). BMI was highest in participants who consumed meat most frequently, and some previous studies have found that high meat consumption is associated with weight gain [56, 57], but it is unclear whether this indicates any specific impact of meat or an association in these populations of high meat intakes with high total energy intakes. The associations of meat with disease risk reported here which remain after adjustment for BMI might still be due to higher adiposity, because BMI is not a perfect measure of this characteristic; we observed similar effects when adjusting for waist circumference (results not shown), but, as with BMI, waist circumference is not a perfect measure of adiposity and there could still be residual confounding.

### Strengths and limitations

As far as we are aware, this is the first outcome-wide study of meat intake and risk of 25 common conditions (other than cancer). Additional strengths of this study include the large size of the cohort, its prospective design, and the comprehensive array of confounders considered. This allowed us to investigate a large number of common conditions and thus avoid outcome selection bias, while simultaneously controlling for confounding. Additionally, we used national record linkage to ascertain information on disease incidence, which is objective and minimises selective loss to follow-up. Nevertheless, some potential methodological issues should be considered when interpreting our findings. Some measurement error would have occurred while measuring meat consumption at baseline; however, we reduced the impact of random error and short-term variation in diet by using the repeated 24-h recall WebQ data and applying corrected intakes to each category of the baseline intakes. Another limitation was that the touchscreen dietary questionnaire only included a subset of food groups and food items and therefore total dietary intake could not be calculated, and confounding by energy balance could not be directly accounted for. We addressed this by adjusting for BMI, physical activity, and other dietary factors [24]; however, there might still be some residual confounding by energy intake. Likewise, participants who consumed high amounts of unprocessed red meat also consumed high amounts of processed meat. Therefore, we could not mutually adjust the meat types, and there may be residual confounding. Multiple testing might have led to some spurious findings; we addressed this by using Bonferroni correction, but this is a stringent approach and it is possible that some real associations did not meet the Bonferroni threshold. Another consideration is the use of hospital records for incident case ascertainment. Some conditions might only require hospital use at later stages (e.g. diabetes), and therefore, some admissions might reflect prevalent and/or more severe cases. Finally, given the observational nature of this study, it is possible that there is still unmeasured confounding, residual confounding, and reverse causality. For instance, in analyses restricted to never smokers, some of the adjusted risk estimates were lower than in the main analysis (e.g. for unprocessed red meat intake and diabetes and for processed meat intake and IHD), suggesting that even after adjustment for smoking there may be residual confounding. However, most of our results were similar after excluding participants who smoked or formerly smoked and after excluding the first 4 years of follow-up.

## Conclusions

Our findings from this large, prospective study of British adults show that meat consumption is associated with higher risks of several common conditions but a lower risk of IDA. The higher risks are at least partly accounted for by higher BMI, and some of the associations remaining after adjusting for BMI or waist circumference may still be due to other aspects of adiposity. Additional research is needed to evaluate whether these differences in risk reflect causal relationships, and if so what proportion of incident cases for these different outcomes that could be prevented by decreasing meat consumption.

## Supplementary Information

**Additional file 1 Methods 1.** Assessment of dietary intake for meat. **Methods 2.** Assessment of health outcomes. **Methods 3.** Covariates. **Table 1.** Disease outcome definition and exclusion criteria. **Table 2.** Baseline characteristics of participants by total meat intake in UK Biobank (*n* = 467,384). **Table 3.** Baseline characteristics of participants by unprocessed red meat intake in UK Biobank (*n* = 468,328). **Table 4.** Baseline characteristics of participants by processed meat intake in UK Biobank (*n* = 472,844). **Table 5.** Baseline characteristics of participants by poultry meat intake in UK Biobank (*n* = 473,011). **Table 6**. Risk of 25 common conditions by total meat intake in UK Biobank. **Table 7.** Risk of 25 common conditions by unprocessed red and processed meat intake in UK Biobank. **Table 8.** Risk of 25 common conditions by unprocessed red meat intake in UK Biobank. **Table 9.** Risk of 25 common conditions by processed meat intake in UK Biobank. **Table 10.** Risk of 25 common conditions by poultry meat intake in UK Biobank. **Figure 1**. Participant flow chart of the study. **Figure 2.** Risk of 25 common conditions by higher daily intake of total meat excluding the first 4 years of follow-up, in never smokers. **Figure 3.** Risk of 25 common conditions by higher daily intake of unprocessed red and processed meat excluding the first 4 years of follow-up, in never smokers. **Figure 4.** Risk of 25 common conditions by higher daily intake of unprocessed red meat excluding the first 4 years of follow-up, in never smokers. **Figure 5.** Risk of 25 common conditions by higher daily intake of processed meat excluding the first 4 years of follow-up, in never smokers. **Figure 6.** Risk of 25 common conditions by higher daily intake of poultry meat excluding the first 4 years of follow-up, in never smokers. **Figure 7.** Risk of 25 common conditions per 100 g/day higher daily intake of total meat.

## Data Availability

The datasets generated/and or analysed in the current study will be made available for bona fide researchers who apply to use the UK Biobank data set by registering and applying at http://www.ukbiobank.ac.uk/register-apply.

## Abbreviations

BMI: Body mass index
CI: Confidence interval
GERD: Gastro-oesophageal reflux disease
HR: Hazard ratio
HRT: Hormone replacement therapy
ICD: International Statistical Classification of Diseases
IDA: Iron deficiency anaemia
IHD: Ischaemic heart disease
MET: Metabolic equivalent
OCP: Oral contraceptive pill
