# Supplementary material for: Meat consumption and risk of 25 common conditions: outcome-wide analyses in 475,000 men and women in the UK Biobank study
Source: BMC Med. 2021 Mar 2;19:53. doi: 10.1186/s12916-021-01922-9 (PMC7923515; doi:10.1186/s12916-021-01922-9)
Supplement: Supplementary file 1 — Additional file 1 Methods 1. Assessment of dietary intake for meat. Methods 2. Assessment of health outcomes. Methods 3. Covariates. Table 1. Disease outcome definition and exclusion criteria. Table 2. Baseline characteristics of participants by total meat intake in UK Biobank (n = 467,384). Table 3. Baseline characteristics of participants by unprocessed red meat intake in UK Biobank (n = 468,328). Table 4. Baseline characteristics of participants by processed meat intake in UK Biobank (n = 472,844). Table 5. Baseline characteristics of participants by poultry meat intake in UK Biobank (n = 473,011). Table 6. Risk of 25 common conditions by total meat intake in UK Biobank. Table 7. Risk of 25 common conditions by unprocessed red and processed meat intake in UK Biobank. Table 8. Risk of 25 common conditions by unprocessed red meat intake in UK Biobank. Table 9. Risk of 25 common conditions by processed meat intake in UK Biobank. Table 10. Risk of 25 common conditions by poultry meat intake in UK Biobank. Figure 1. Participant flow chart of the study. Figure 2. Risk of 25 common conditions by higher daily intake of total meat excluding the first 4 years of follow-up, in never smokers. Figure 3. Risk of 25 common conditions by higher daily intake of unprocessed red and processed meat excluding the first 4 years of follow-up, in never smokers. Figure 4. Risk of 25 common conditions by higher daily intake of unprocessed red meat excluding the first 4 years of follow-up, in never smokers. Figure 5. Risk of 25 common conditions by higher daily intake of processed meat excluding the first 4 years of follow-up, in never smokers. Figure 6. Risk of 25 common conditions by higher daily intake of poultry meat excluding the first 4 years of follow-up, in never smokers. Figure 7. Risk of 25 common conditions per 100 g/day higher daily intake of total meat. [file 12916_2021_1922_MOESM1_ESM.docx]

# Additional File 1

[Additional file 1: Methods 2](#_Toc62026437)

[Additional file 1: Methods 1. Assessment of dietary intake for meat. 2](#_Toc62026438)

[Additional file 1: Methods 2. Assessment of health outcomes. 3](#_Toc62026439)

[Additional file 1: Methods 3. Covariates. 4](#_Toc62026440)

[Additional file 1: Tables 9](#_Toc62026441)

[Additional file 1: Table 1. Disease outcome definition and exclusion criteria. 9](#_Toc62026442)

[Additional file 1: Table 2. Baseline characteristics of participants by total meat intake in UK Biobank (n= 467 384^a^). 15](#_Toc62026443)

[Additional file 1:Table 3. Baseline characteristics of participants by unprocessed red meat intake in UK Biobank (n=468 328^a^). 18](#_Toc62026444)

[Additional file 1: Table 4. Baseline characteristics of participants by processed meat intake in UK Biobank (n=472 844^a^). 21](#_Toc62026445)

[Additional file 1: Table 5. Baseline characteristics of participants by poultry meat intake in UK Biobank (n=473 011^a^). 24](#_Toc62026446)

[Additional file 1: Table 6. Risk of 25 common conditions by total meat intake in UK Biobank. 27](#_Toc62026447)

[Additional file 1: Table 7. Risk of 25 common conditions by unprocessed red and processed meat intake in UK Biobank. 32](#_Toc62026448)

[Additional file 1: Table 8. Risk of 25 common conditions by unprocessed red meat intake in UK Biobank 37](#_Toc62026449)

[Additional file 1: Table 9. Risk of 25 common conditions by processed meat intake in UK Biobank. 42](#_Toc62026450)

[Additional file 1: Table 10. Risk of 25 common conditions by poultry meat intake in UK Biobank. 47](#_Toc62026451)

[Additional file 1: Figures 52](#_Toc62026452)

[Additional file 1: Fig. 1. Participant flow chart of the study. 52](#_Toc62026453)

[Additional file 1: Fig. 2. Risk of 25 common conditions by higher daily intake of total meat excluding the first 4 years of follow-up, in never smokers. 53](#_Toc62026454)

[Additional file 1: Fig. 3. Risk of 25 common conditions by higher daily intake of unprocessed red and processed meat excluding the first 4 years of follow-up, in never smokers. 54](#_Toc62026455)

[Additional file 1: Fig. 4. Risk of 25 common conditions by higher daily intake of unprocessed red meat excluding the first 4 years of follow-up, in never smokers. 55](#_Toc62026456)

[Additional file 1: Fig. 5. Risk of 25 common conditions by higher daily intake of processed meat excluding the first 4 years of follow-up, in never smokers. 56](#_Toc62026457)

[Additional file 1: Fig. 6. Risk of 25 common conditions by higher daily intake of poultry meat excluding the first 4 years of follow-up, in never smokers. 57](#_Toc62026458)

[Additional file 1: Fig. 7. Risk of 25 common conditions per 100 g/day higher daily intake of total meat. 58](#_Toc62026459)

# Additional file 1: Methods

## Additional file 1: Methods 1. Assessment of dietary intake for meat.

**Touchscreen**

Dietary intake was assessed using a touchscreen dietary questionnaire administered to all participants at baseline. The touchscreen questionnaire included 5 questions on meat. These included the following: ‘How often do you eat processed meats (such as bacon, ham, sausages, meat pies, kebabs, burgers, chicken nuggets)’, ‘How often do you eat chicken, turkey or other poultry? (Do not count processed meats)’, ‘How often do you eat beef? (Do not count processed meats)', ‘How often do you eat lamb/mutton? (Do not count processed meats)’ and ‘How often do you eat pork? (Do not count processed meats such as bacon or ham)’. We converted the responses to each of the five questions on meat into weekly intake equivalents as follows: 0, 0.5, 1, 3, 5.5 and 7 times per week. We then categorized meat intake frequencies into the following groups: total meat (< 3, 3-4, 5-6 and ≥ 7 times per week), which was constructed by summing intake frequencies for all meat types together; unprocessed red and processed meat (0 to 1, 2, 3-4 and ≥ 5 times per week), which was constructed by summing intake frequencies for unprocessed red and processed meat together; unprocessed red meat (< 1, 1, 2-3 and ≥ 4 times per week); processed meat (0,<1,1 and ≥ 2 times per week); and poultry meat (0-1, 2 and ≥ 3 times per week). These categories were determined according to the distribution of the data in order to create approximately equal-sized groups

**Oxford WebQ questionnaire**

Dietary intake was also assessed through the Oxford WebQ questionnaire, an online 24-hour recall questionnaire that included information on 206 food items that was administered in 2009 (available for a subsample recruited after its introduction), and through email four times between February 2011 and June 2012 to all participants who provided UK Biobank with an email address and agreed to be re-contacted. Participants were asked to select how many portions of each food item they consumed over the previous 24 hours, and we calculated mean grams per/day by multiplying frequencies of consumption by standard portion sizes for each meat type. The following portion sizes were used: unprocessed red meat (beef 120 g, pork 120 g, lamb 120g), processed meat (sausage 30g, crumbed chicken 100g, bacon 46g, ham 23g), and poultry meat (chicken 130g). Similar foods were then grouped together into meat-types, accounting for the different serving size of each meat type, to match the touchscreen dietary questionnaire. To account for variation in daily intake in the diet and better capture usual dietary intake, we averaged daily intakes recorded over a minimum of three WebQ questionnaires. To account for extreme intakes we estimated means from participants with reliable WebQ dietary data (defined as having an estimated daily energy intake between 3349 kJ (800 kcal) and 16 747 kJ (4000 kcal) for men and between 2093 kJ (500 kcal) and 14 654 kJ (3500 kcal) for women)); reliable data were available for around 69 000 participants.

**Measurement error correction**

We then assigned the mean WebQ intakes in these participants who had completed at least three WebQs to each touchscreen category for each type of meat. Using these means, we calculated a trend variable using the following increments: unprocessed red meat (50 gram (g)/day increase); processed meat (20 g/day); poultry meat (30 g/day); unprocessed red and processed meat (70 g/day); and total meat (100 g/day). These increments were chosen to be close to the average portions of each type of meat consumed in this cohort.

We also assessed measurement error correction by calculating mean g/day intakes of unprocessed red meat for participants who completed the 2012 follow-up touchscreen questionnaire (n=~20,000) and then assigned these mean intakes to the touchscreen dietary questionnaire intake categories defined for all participants. Overall, we found similar estimates using either method. For example, a 50 g/day higher intake of unprocessed red meat was associated with a 16% higher risk of IHD (95 % CI 1.08-1.25) using the main error correction method (3+ WebQs) and 15% higher risk of IHD (1.07-1.22) using the alternative error correction method (follow-up touchscreen questionnaire).

## Additional file 1: Methods 2. Assessment of health outcomes.

Disease end points, information on diagnoses or procedures associated with hospital admissions, and causes of death were all coded according to the 9^th^ and 10^th^ revisions of the World Health Organization’s International Classification of Diseases (ICD-9 and ICD-10), and the Office of Population Censuses and Surveys classification of surgical operations and procedures (OPCS-4), fourth revision (see Additional file 1: Table 1 for exclusion, diagnosis and procedure codes). Admissions were defined according to the primary diagnosis/and or operation codes recorded during the admission (some of which were first recorded at death).

## Additional file 1: Methods 3. Covariates.

**Socio demographic**

**Age**

Participants provided information about their age at recruitment which we then categorized into the following age groups: 37-44, 45-49, 50-54, 55-59, 60-64 and ≥ 65 years of age.

**Region**

Participants completed their baseline assessments in the 22 recruitment centres, which were then grouped into 10 respective regions accordingly: London (recruitment centres: St Bartholomew’s Hospital, Hounslow, Croydon) Wales (recruitment centres: Swansea, Wrexham, Cardiff), North-West England (recruitment centres: Stockport, Manchester, Liverpool, Bury), North-East England (recruitment centres: Newcastle, Middlesbrough), Yorkshire (recruitment centres: Leeds, Sheffield), West Midlands (recruitment centres: Stoke, Birmingham) East Midlands (recruitment centre: Nottingham), South-East England (recruitment centres: Oxford, Reading), South-West England (recruitment centre: Bristol), Scotland (recruitment centres: Glasgow, Edinburgh).

**Race**

Participants were asked about their race at baseline with the question ‘What is your ethnic group?’ Response options included ‘White’, ‘Mixed’, ‘Asian or Asian British’, ‘Black or Black British’, ‘Chinese’, ‘Other ethnic group’, ‘Do not know’ or ‘Prefer not to answer’ and four supporting questions within groups. We then re-categorised race into 4 groups: White (White, British, Irish or any other white background); Asian or Asian British (Asian or Asian British, Chinese, Indian, Pakistani, Bangladeshi or any other Asian background); Black or Black British ( Black or Black British, Caribbean, African or any other Black background); Mixed race or Other (any other ethnic groups or mixed race); and unknown (included participants who did not know or preferred not to answer).We also categorized race into 2 groups: White (White, British, Irish or any other white background), Non-white (Asian or Asian British, Chinese, Indian, Pakistani, Bangladeshi or any other Asian background, Black or Black British, Caribbean, African or any other Black background, any other ethnic groups or mixed race); and unknown (included participants who did not know or preferred not to answer) and used this for outcomes with fewer cases.

**Townsend deprivation score**

Material deprivation was assessed at baseline using the Townsend index score, which was assigned based on the postcode of residence. The index was calculated using four variables that collected information on percentage of unemployment, percentage of overcrowded households, percentage of people with no car ownership and percentage of non-home owners. We then categorised the score into quintiles, ranging from negative values (areas of low material deprivation) to positive scores (areas with high material deprivation) and unknown (for participants with missing information) [1].

**Education**

For education, participants were asked ‘Which of the following qualifications do you have?’ at baseline. Response options included: ‘College or University degree’, ‘A levels/AS levels or equivalent’, ‘O levels/GCSEs or equivalent’, ‘CSEs or equivalent’, ‘NVQ or HND or HNC or equivalent’, ‘Other professional qualifications e.g.: nursing, teaching’, ‘None of the above’ and ‘Prefer not to answer’. We then categorised qualification into 3 groups: College or university degree/vocational qualification (including College or University degree, NVQ or HND or HNC or equivalent, and Other professional qualifications e.g.: nursing, teaching); National examination at ages 17‑18 (including A levels/AS levels or equivalent’), National examination at age 16 (including O levels/GCSEs or equivalent, and CSEs or equivalent), and unknown (if answered ‘None of the above’ and ‘Prefer not to answer’).

**Employment**

Employment was assessed at baseline with the question ‘Which of the following describes your current situation?’ Response options included: ‘In paid employment or self-employed’, ‘Retired’, ‘Looking after home and/or family’, ‘Unable to work because of sickness or disability’, ‘Unemployed’, ‘Doing unpaid or voluntary work’, ‘Full or part time student’, ‘None of the above’ and ‘Prefer not to answer’. We then categorised employment into 3 groups: Paid/self-employment (including participants who reported being in paid employment or self-employed), ‘retired’ (participants who reported no paid employment); ‘not in paid employment’ (included those reporting no paid employment e.g. caring responsibilities, being a student, unemployed or inability to work); and unknown (including participants who did not answer, preferred not to answer or who’s answers did not fit into the other groups).

**Physical measurements**

***Anthropometric variables***

Standing height was measured at baseline according to standard protocol, using the SECA 240 Height Measure. Weight was measured at baseline using the Tanita BC 418 body composition analyser or using standard scales for participants who did not undergo bioimpedence analysis. Body mass index (BMI) was then calculated by dividing weight in kilograms by the square of height in metres and categorised as into sex-specific quintiles as such: Q1 (women: mean 21 kg/m^2^, men; mean 23 kg/m^2^),Q2 (women: mean 24 kg/m^2^, men: mean 26 kg/m^2^), Q3 (women: mean 26 kg/m^2^, men: mean 27 kg/m^2^), Q4 (women: mean 29 kg/m^2^,men: mean 29 kg/m^2^), Q5 (women: mean 35 kg/m^2^, men: mean 34 kg/m^2^); and unknown (for participants with missing height and/or weight data).

**Lifestyle**

***Smoking***

Participants were asked about their current smoking status at baseline using two questions: ‘Do you smoke tobacco now?’(with response options including ‘Yes, on most or all days’, ‘Only occasionally’, ‘No’ and ‘Prefer not to answer’) and ‘In the past, how often have you smoked tobacco?’ (with response options including ‘Smoked on most or all days’, ‘Smoked occasionally’, ‘Just tried once or twice’, ‘I have never smoked’, ‘Prefer not to answer’). Participants were also asked about the number of cigarettes they smoked per day with the question ‘About how many cigarettes do you smoke on average each day?’(with response options including choosing a number, ‘less than one’, ‘do not know’ and ‘prefer not to say’). Using the data from these questions, we then categorised smoking status into 5 groups including: ‘never smoker’, ‘former smoker’, ‘current smoker of less than 15 cigarettes’, ‘current smoker of more than 15 cigarettes’ and ‘current smoker of unknown amount of cigarettes’ and ‘unknown’ for participants with missing data.

***Physical activity***

For physical activity, participants were asked a group of questions at baseline that were taken from the International Physical activity questionnaire (IPAQ) short form [2]. The questions assessed the frequency, duration (only for participants who answered the question on frequency as more than zero), and types of physical activity undertaken. Using the answers to these questions, we calculated Excess metabolic equivalents (METs) (which only include activity above basal metabolic rate [3]) as follows: sum of walking time per week times 2.3, moderate activity per week (3.0 Excess METs), and vigorous activity per week (7.0 excess METs), and zero METs (where the type of activity was missing). Using the excess METs categories, we then calculated total physical activity using 3 groups: ‘low’ (<10 excess METs per week), ‘moderate’ (>10 to <50 excess METs per week), ‘high’ (>50 excess METs per week), and ‘unknown’ (for participants with missing information, or who reported that they were unable to walk).

***Alcohol intake***

Participants were asked about their weekly and monthly consumption of pints of beer, glasses of red wine, glasses of white wine/champagne, glasses of fortified wine, measures of spirits/liqueurs and glasses of other alcohol at baseline. Additionally, participants were asked more generally how often they drank alcohol with the possible responses including: ‘daily or almost daily’, ‘three or four times a week’, ‘once or twice a week’, ‘one to three times a month’, ‘special occasions only’, ‘never’, and ‘prefer not to answer’. We assigned an average of 20 grams of alcohol per serving for a pint of beer and 10 grams of alcohol per serving for all other types of alcohol. We then created a total alcohol intake variable by grouping intakes as follows: ‘non-drinker’, ‘< 1 g/day’, ‘1-<10 g/day’ ‘10-<20g/day’, ‘≥20 g/day’ and ‘unknown’. We categorised participants who reported drinking only on special occasions into the ‘< 1 g/day’ category and those who reported never drinking alcohol into the ‘non-drinker’ category.

**Diet**

***Fruit and vegetable intake***

The touchscreen questionnaire included 29 questions on diet. For fruit, participants were asked ‘About how many pieces of fresh fruit would you eat per day? (Count one apple, one banana, 10 grapes etc. as one piece; put `0’ if you do not eat any)’ at baseline. For vegetables, participants were asked about their consumption of cooked vegetables (‘On average, how many heaped tablespoons of cooked vegetables would you eat per day? [Do not include potatoes; put ‘0’ if you do not eat any]’) and raw vegetables [‘On average, how many heaped tablespoons of salad or raw vegetables would you eat per day? (Include lettuce, tomato in sandwiches; put `0’ if you do not eat any]’) at baseline. One piece of fruit and two heaped tablespoons of vegetables, respectively were considered as one serving. Accordingly, we categorized participants into the following groups: < 3 servings’, ‘3 to <4 servings’, ‘4 to <6 servings’, ‘≥6 serving per day’, and ‘unknown’.

***Cereal fibre intake***

Participants were asked about their frequency of bread and cereal consumption at baseline with the questions ‘How many slices of bread do you eat each week’ and ‘How many bowls of cereal do you eat a week?’. Response options included open ended answers and ‘less than one’, ‘do not know’ and ‘prefer not to answer’. Participants who responded with >1 or more times to either of these questions were also asked for bread: ‘What type of bread do you mainly eat?’, with response options that included ‘White/Brown/Wholemeal or wholegrain’, ‘Other type of bread’, ‘Do not know’, ‘Prefer not to answer’ and for cereal: ‘What type of cereal do you mainly eat?’ with response options that included ‘Bran cereal’ (e.g. All Bran, Branflakes), ‘Biscuit cereal’ (e.g. Weetabix), ‘Oat cereal’ (e.g. Ready Brek, porridge), ‘Muesli’, ‘Other ‘(e.g. Cornflakes, Frosties), ‘Do not know’, and ‘Prefer not to answer’. Using the responses to these questions, we calculated a fibre score for bread and cereal by multiplying fibre intake frequencies by the fibre content of the type of bread and cereals being consumed (see original publication[4] for additional details). We then categorized fibre intakes from bread and cereal into sex-specific intakes Q1 (women: mean 1.1 g, men: mean 1.3 g), Q2 (women: mean 2.5g, men: mean 2.8g), Q3 (women: mean 3.7g, men: mean 4.3g), Q4(women: mean 5.3 g, men: mean 6.2 g), Q5 (women: mean 8.4g, men: mean 9.9g); and ‘unknown’ (for participants who did not report cereal or bread intakes or preferred not to answer).

***Oily fish intake and non-oily fish intake***

Participants were asked about their fish consumption with the questions: How often do you eat oily fish? (e.g. sardines, salmon, mackerel, herring)’ and ‘How often do you eat other types of fish (e.g. cod, tinned tuna, haddock)’. Response options for these questions included: ‘never’, ‘less than once a week’, ‘once a week’, ‘2-4 times a week’, ‘5-6 times a week’, ‘once or more daily’, ‘do not know’ and ‘prefer not to answer’. Using the responses to the questions, we categorised intakes into 4 categories for oily fish (0 times/week, <1 time/week, 1 time/week, and >2 times/week) and 3 categories for non-oily fish (<1 time/week, 1 time/week, and ≥2 times/week).

**Women factors**

***Parity***

For parity, participants were asked ‘How many children have you given birth to? (Please include live births only)’ at baseline. We then categorised parity into ‘nulliparous’, ‘one to two’, ‘three or more’, and ‘unknown’ (for participants with missing information or who preferred not to answer).

***Hormone replacement therapy (HRT)***

Participants were asked about their use of HRT at baseline with the questions ‘Have you ever used hormone replacement therapy (HRT)?’ (response options included ‘yes’, ‘no’, ’don’t know’, ‘prefer not answer’). Participants who indicated that they had used HRT were then also asked ‘How old were you when you last used HRT?’ and ‘How old were you when you first used HRT’. Using the responses to these questions, we then ‘unknown’ (for those who answered ‘do not know’, ‘prefer not to answer’ or who were missing information).

***Oral contraceptive pill (OCP)***

Participants were asked about their use of OCP at baseline with the questions ‘Have you ever taken the contraceptive pill? (include the mini pill)’ (response options included ‘yes’, ‘no’, ’don’t know’, ‘prefer not answer’). Participants who indicated that they had used HRT were then also asked ‘How old were you when you last used the contraceptive pill?’ and ‘How old were you when you first went on the contraceptive pill. Using the responses to these questions, we then categorised participants into these groups: ’never’ (if they replied no to the first question), ‘past’, ‘current’, and ‘unknown’ (for those who answered ‘do not know’, ‘prefer not to answer’ or who were missing information).

***Menopausal status***

Participants were asked about their menopausal status at baseline with the question ‘Have you had your menopause (periods stopped)?’. Response options included ‘yes’, ‘no’, ‘not sure had hysterectomy’, ‘not sure other reason’ or ‘prefer not to answer’. For participants who responded with ‘not sure had hysterectomy’, ‘not sure other reason’ or ‘prefer not to answer’, information from other questions on bilateral oophorectomy, use of HRT, and age at recruitment was used to categorise participants. We categorized participants as premenopausal if they reported not having their menopause, were younger than 45 and did not report a bilateral oophorectomy. We categorized participants as postmenopausal if they answered yes to ‘Have you had your menopause (periods stopped)?’, were over the age of 53, answered yes to the question ‘Have you had both ovaries removed?’ or reported HRT use. Additionally, we categorized participants as postmenopausal if they reported to not have their menopause but were over the age of 55 at recruitment. We categorised participants as ‘unknown’ if they reported not having their period but having had a bilateral oophorectomy or having used HRT, or if they had missing information on menopausal status and could not be reassigned using the additional information as described above.

**References**

1. Townsend P, Phillimore P, Beattie A. Health and deprivation: inequality and the North. Routledge; 1988.

2. Sjöström M, Ainsworth B, Bauman A, Bull F, Craig C, Sallis J. Guidelines for data processing and analysis of the International Physical Activity Questionnaire (IPAQ)–short and long forms. IPAQ core group. 2005.

3. Perez-Cornago A, Key TJ, Allen NE, Fensom GK, Bradbury KE, Martin RM et al. Prospective investigation of risk factors for prostate cancer in the UK Biobank cohort study. British journal of cancer. 2017;117(10):1562-71. doi:10.1038/bjc.2017.312.

4. Bradbury KE, Young HJ, Guo W, Key TJ. Dietary assessment in UK Biobank: an evaluation of the performance of the touchscreen dietary questionnaire. Journal of nutritional science. 2018;7:e6. doi:10.1017/jns.2017.66.

# Additional file 1: Tables

| Additional file 1: Table 1. Disease outcome definition and exclusion criteria. | | | | | | | | |  |
| --- | --- | --- | --- | --- | --- | --- | --- | --- | --- |
| Admission cause | Outcome definition using ICD-10 | Relevant procedure code using OPSC-4 definition | Exclusion criteria using ICD-10 | Exclusion criteria using OPSC-4 | Exclusion criteria using ICD-9 | Exclusion criteria using touchscreen | Exclusion criteria using UKB interviews for outcomes^a^ | Exclusion criteria using UKB interviews for procedures^b^ | Total N/cases^c^ |
| Circulatory disease |  |  |  |  |  |  |  |  |  |
| Ischaemic heart diseases | I20, I21, I22, I23, I24, I25 |  | I48, I20, I21, I22, I23, I24, I25, G45, I60, I61, I62, I63, I64, I65, I66, I67, I68, I69 |  | 427.31, 427.32, 410, 411, 412, 413, 414, 430, 431, 432, 433, 434, 435, 436, 437, 438 | UKB variable 'Has a doctor ever told you that you have the following conditions 6150?'  1) Heart attack 2) angina  3) stroke | 1471 atrial fibrillation  1483 atrial flutter  1074 angina  1075 heart attack/myocardial infarction  1081 stroke  1082 transient ischaemic attack1083 subdural haemorrhage/haematoma  1086 subarachnoid haemorrhage |  | 437094/13377 |
| Atrial fibrillation and flutter | I48 |  | I48, I20, I21, I22, I23, I24, I25, G45, I60, I61, I62, I63, I64, I65, I66, I67, I68, I69 |  | 427.31, 427.32, 410, 411, 412, 413, 414, 430, 431, 432, 433, 434, 435, 436, 437, 438 | UKB variable 'Has a doctor ever told you that you have the following conditions 6150?'  1) Heart attack, 2) angina,  3) stroke | 1471 atrial fibrillation  1483 atrial flutter  1074 angina  1075 heart attack/myocardial infarction  1081 stroke  1082 transient ischaemic attack1083 subdural haemorrhage/haematoma  1086 subarachnoid haemorrhage |  | 437094/4813 |
| Cerebrovascular disease | G45 I60 I61 I62 I63 I64 I65 I66 I67 I68 I69 |  | I48, I20, I21, I22, I23, I24, I25, G45, I60, I61, I62, I63, I64, I65, I66, I67, I68, I69 |  | 427.31, 427.32, 410, 411, 412, 413, 414, 430, 431, 432, 433, 434, 435, 436, 437, 438 | UKB variable 'Has a doctor ever told you that you have the following conditions 6150?'  1) Heart attack2) angina  3) stroke | 1471 atrial fibrillation  1483 atrial flutter  1074 angina  1075 heart attack/myocardial infarction  1081 stroke  1082 transient ischaemic attack 1083 subdural haemorrhage/haematoma  1086 subarachnoid haemorrhage |  | 437094/5445 |
| ischaemic stroke | I63 |  | I48, I20, I21, I22, I23, I24, I25, G45, I60, I61, I62, I63, I64, I65, I66, I67, I68, I69 |  | 427.31, 427.32, 410, 411, 412, 413, 414, 430, 431, 432, 433, 434, 435, 436, 437, 438 | UKB variable 'Has a doctor ever told you that you have the following conditions 6150?'  1) Heart attack 2) angina,  3) stroke | 1471 atrial fibrillation  1483 atrial flutter  1074 angina  1075 heart attack/myocardial infarction  1081 stroke  1082 transient ischaemic attack1083 subdural haemorrhage/haematoma  1086 subarachnoid haemorrhage |  | 437094/2396 |
| haemorrhagic stroke | I60 I61 |  | I48, I20, I21, I22, I23, I24, I25, G45, I60, I61, I62, I63, I64, I65, I66, I67, I68, I69 |  | 427.31, 427.32, 410, 411, 412, 413, 414, 430, 431, 432, 433, 434, 435, 436, 437, 438 | UKB variable 'Has a doctor ever told you that you have the following conditions 6150?'  1) Heart attack2) angina  3) stroke | 1471 atrial fibrillation  1483 atrial flutter  1074 angina  1075 heart attack/myocardial infarction  1081 stroke  1082 transient ischaemic attack 1083 subdural haemorrhage/haematoma  1086 subarachnoid haemorrhage |  | 437094/961 |
| Venous thromboembolism | I26, I80, 181, I82 |  | I26, I80, 181, I82 |  | 415.1, 451, 452, 453 |  | 1068 venous thromboembolic disease  1094 deep venous thrombosis |  | 464439/3839 |
| Varicose veins of lower extremities | I83 | L84 L85 L86 L87 L88 | I83 | L84 L85 L86 L87 L88 | 454 |  | 1494 varicose veins | 1479 varicose vein surgery | 452437/2670 |
| Haemorrhoids | I84 | H51, H52, H53 | I84 | H51, H52, H53 | 455 |  | 1505 haemorrhoids / piles | 1483 haemorrhoidectomy / piles surgery/ banding of piles | 455013/8448 |
| Respiratory disease |  |  |  |  |  |  |  |  |  |
| pneumonia | J18 |  | J18 |  | 480, 481, 482, 483, 484, 485, 486 |  | 1398 pneumonia |  | 466395/6563 |
| Digestive disease |  |  |  |  |  |  |  |  |  |
| Gastro-oesophageal reflux disease (GERD) | K21 |  | K21 |  | 530.11, 530.81 |  | 1138 gastro-oesophageal reflux (GERD) |  | 446551/7153 |
| Gastritis and duodenitis | K29 |  | K29 |  | 535 |  |  |  | 457833/11609 |
| Inguinal hernia | K40 | T19, T20, T21 | K40 | T19, T20, T21 | 550 |  | 1513 inguinal hernia | 1403 inguinal/femoral hernia repair  1563 inguinal hernia repair  1402 hernia surgery | 447643/8146 |
| Non-infective enteritis and colitis | K50, K51, K52 |  | K50, K51, K52 |  | 555, 556, 558 |  | 1459 colitis/not crohns or ulcerative colitis  1462 crohns disease  1463 ulcerative colitis |  | 460610/6366 |
| Diverticular disease of intestine | K57 |  | K57 |  | 562 |  | 1458 diverticular disease/diverticulitis |  | 462292/13023 |
| Colon polyps | D12, K63.5 | H20.1, H20.2, H20.3, H20.4, H20.5, H20.6, H22.1, H23.1,  H25.1, H28.1 | D12, K63.5 | H20.1, H20.2, H20.3, H20.4, H20.5, H20.6, H22.1, H23.1,  H25.1, H28.1 | 211.3, 211.4 |  | 1460 rectal or colon adenoma/polyps | 1463 rectal or colon polypectomy | 452873/38019 |
| gallbladder disease | K80, K81 | J18 | K80, K81 | J18 | 574, 575.0, 575.1 |  | 1161 gall bladder disease | 1455 cholecystectomy/  gall bladder removal | 455324/9090 |
| Joint disorder |  |  |  |  |  |  |  |  |  |
| Osteoarthritis | M15, M16, M17, M18, M19, M47 |  | M15, M16, M17, M18, M19, M47 |  | 715, 721 |  | 1465 osteoarthritis |  | 423418/18369 |
| Genitourinary disease |  |  |  |  |  |  |  |  |  |
| Kidney stones | N20, N23 |  | N20, N23 |  | 592, 788.0 |  | 1197 kidney stone/ureter stone/bladder stone | 1197percutaneous/open kidney stone surgery/lithotripsy | 467673/2499 |
| Urinary tract infection | N39.0 |  | N39.0 |  | 599.0 |  | 1196 urinary tract infection/kidney infection |  | 467055/5340 |
| Hyperplasia of prostate | N40 |  | N40 |  | 600 |  | 1516 benign prostatic hypertrophy  1396 enlarged prostate |  | 209137/3613 |
| Female genital prolapse | N81 | M51, M52, M53, P22, P23, P24 | N81 | M51, M52, M53, P22, P23, P24 | 618 |  | 1353 vaginal prolapse/uterine prolapse | 1361 vaginal prolapse/  colposuspension | 243028/6145 |
| Other diseases |  |  |  |  |  |  |  |  |  |
| Uterine fibroids | D25 | Q10, Q18, Q074, Q089, Q171, Q092, Q093, Q161, T43 | D25 | Q10, Q18, Q074, Q089, Q171, Q092, Q093, Q161, T43 | 218 | UKB variable 'Ever had hysterectomy 3591'  1)yes  UKB variable 'Ever had menopause 2724'  2) don't know had hysterectomy | 1351 uterine fibroids | 1509 myomectomy/fibroids removed  1357 hysterectomy 1358 hysterectomy with oophorectomy 1359 hysterectomy with cervical sparing | 184246/9411 |
| Iron deficiency anaemia (IDA) | D50 |  | D50 |  | 280 |  | 1330 iron deficiency anaemia |  | 469192/4833 |
| Diabetes mellitus | E10, E11, E12, E13, E14 |  | E10, E11, E12, E13, E14 |  | 250 | UKB variable 'Dr. diagnosed diabetes 2443' 1)yes    UKB variable 'Taking medications 6177'  3) insulin | 1220 diabetes  1221 gestational diabetes  1222 type 1 diabetes  1223 type 2 diabetes |  | 449259/9890 |
| Carpal tunnel syndrome | G56.0 | A65.1 | G56.0 | A65.1 | 354.0 |  | 1541 carpal tunnel syndrome | 1501 carpal tunnel surgery | 465539/5167 |
| Cataract | H25, H26, Q120 | C71, C72, C73, C74, C75 | H25, H26, Q120, E10, E11, E13, E14 | C71, C72, C73, C74, C75 | 366, 250 | UKB variable 'Have eye problems 6148'  4) Cataract  UKB variable 'Dr. diagnosed diabetes 2443' 1)yes  UKB variable 'Taking medications 6177'  3) insulin | 1278 Cataract 1220 diabetes, 1221 gestational diabetes  1222 type 1 diabetes  1223 type 2 diabetes | 1435 cataract extraction/lens implant | 432180/16432 |
| Cellulitis | L03 |  | L03 |  | 681, 682 |  | 1625 cellulitis |  | 470763/3486 |
| ^a^Based on ICD-10 definition and UKB variable 20002 | | | | | | | | |  |
| ^b^Based on OPSC-4 definition and UKB variable 20004  ^c^ From a maximal analysis sample of n=474 985 | | | | | | | | |  |

| Additional file 1: Table 2. Baseline characteristics of participants by total meat intake in UK Biobank (n= 467 384^a^). | | | | |
| --- | --- | --- | --- | --- |
| Characteristic | <3 times/week | 3-4 times/week | 5-6 times/week | > 7times/week |
| Mean (SD) or n (%) | N=57 956 | N=119 279 | N=153 677 | N=136 472 |
| **Sociodemographic** |  |  |  |  |
| Sex, n (%) |  |  |  |  |
| Women | 39 574 (68.3) | 72 015 (60.4) | 83 791 (54.5) | 56 867 (41.7) |
| Men | 18 382 (31.7) | 47 264 (39.6) | 69 886 (45.5) | 79 605 (58.3) |
| Age (years), mean (SD) | 55.5 (8.1) | 57.2 (7.9) | 56.5 (8.0) | 55.7 (8.2) |
| Age (years), n (%) |  |  |  |  |
| 37-44 | 7 252 (12.5) | 10 470 (8.8) | 15 445 (10.1) | 16 651 (12.2) |
| 45-49 | 8 316 (14.3) | 14 022 (11.8) | 20 165 (13.1) | 20 647 (15.1) |
| 50-54 | 9 433 (16.3) | 17 199 (14.4) | 23 729 (15.4) | 21 813 (16.0) |
| 55-59 | 11 132 (19.2) | 22 167 (18.6) | 28 017 (18.2) | 23 657 (17.3) |
| 60-64 | 12 829 (22.1) | 30 865 (25.9) | 37 434 (24.4) | 30 427 (22.3) |
| 65- 73 | 8 994 (15.5) | 24 556 (20.6) | 28 887 (18.8) | 23 277 (17.1) |
| Race, n (%) |  |  |  |  |
| White | 51 849 (89.5) | 113 618 (95.3) | 147 349 (95.9) | 128 388 (94.1) |
| Asian or Asian British | 3 750 (6.5) | 2 086 (1.7) | 2 053 (1.3) | 2 612 (1.9) |
| Black or Black British | 907 (1.6) | 1 583 (1.3) | 1 976 (1.3) | 2 740 (2.0) |
| Mixed race/Other | 1 163 (2.0) | 1 643 (1.4) | 1 846 (1.2) | 2 235 (1.6) |
| Unknown | 287 (0.5) | 349 (0.3) | 453 (0.3) | 497 (0.4) |
| Townsend deprivation, n (%) |  |  |  |  |
| Most affluent (mean -4.7) | 9 319 (16.1) | 24 761 (20.8) | 32 850 (21.4) | 27 393 (20.1) |
| 2 (mean -3.3) | 9 955 (17.2) | 24 471 (20.5) | 32 198 (21.0) | 26 995 (19.8) |
| 3 (mean -2.1) | 11 110 (19.2) | 24 203 (20.3) | 31 440 (20.5) | 26 942 (19.7) |
| 4 (mean -0.1) | 13 288 (22.9) | 23 531 (19.7) | 29 648 (19.3) | 26 863 (19.7) |
| Most deprived (mean 3.8) | 14 214 (24.5) | 22 171 (18.6) | 27 349 (17.8) | 28 101 (20.6) |
| Unknown | 70 (0.1) | 142 (0.1) | 192 (0.1) | 178 (0.1) |
| Qualification, n (%) |  |  |  |  |
| College/university degree/NVQ | 37 607 (64.9) | 70 076 (58.7) | 90 312 (58.8) | 81 269 (59.5) |
| National examination at ages 17‑18 | 3 153 (5.4) | 6 225 (5.2) | 8 514 (5.5) | 7 717 (5.7) |
| National examination at age 16 | 8 167 (14.1) | 19 862 (16.7) | 26 981 (17.6) | 22 751 (16.7) |
| Other/unknown | 9 029 (15.6) | 23 116 (19.4) | 27 870 (18.1) | 24 735 (18.1) |
| Employment, n (%) |  |  |  |  |
| In paid employment | 35 697 (61.6) | 66 455 (55.7) | 88 950 (57.9) | 82 131 (60.2) |
| Pension | 14 504 (25.0) | 39 207 (32.9) | 47 218 (30.7) | 37 278 (27.3) |
| Not in paid employment | 7 020 (12.1) | 12 584 (10.6) | 16 304 (10.6) | 15 832 (11.6) |
| Unknown | 735 (1.3) | 1 033 (0.9) | 1 205 (0.8) | 1 231 (0.9) |
| **Physical measurements** |  |  |  |  |
| BMI (kg/m^2^), mean (SD) | 26.0 (4.6) | 27.0 (4.6) | 27.6 (4.7) | 28.2 (4.9) |
| BMI (kg/m^2^), fifths, n (%) |  |  |  |  |
| Q1 (mean W: 21 kg/m^2^, M: 23 kg/m^2^) | 17 938 (31.0) | 26 259 (22.0) | 29 161 (19.0) | 23 274 (17.1) |
| Q2 (mean W: 24 kg/m^2^, M: 26 kg/m^2^) | 12 316 (21.3) | 24 859 (20.8) | 29 904 (19.5) | 24 179 (17.7) |
| Q3 (mean W: 26 kg/m^2^, M: 27 kg/m^2^) | 10 893 (18.8) | 24 871 (20.9) | 32 382 (21.1) | 26 978 (19.8) |
| Q4 (mean W: 29 kg/m^2^, M: 29 kg/m^2^) | 8 897 (15.4) | 22 530 (18.9) | 30 528 (19.9) | 28 294 (20.7) |
| Q5 (mean W: 35 kg/m^2^, M: 34 kg/m^2^) | 7 555 (13.0) | 20 225 (17.0) | 31 047 (20.2) | 33 078 (24.2) |
| Unknown | 357 (0.6) | 535 (0.4) | 655 (0.4) | 669 (0.5) |
| **Lifestyle** |  |  |  |  |
| Smoking, n (%) |  |  |  |  |
| Never | 33 528 (57.9) | 66 282 (55.6) | 84 640 (55.1) | 71 542 (52.4) |
| Former | 18 841 (32.5) | 40 968 (34.3) | 53 377 (34.7) | 47 271 (34.6) |
| Current <15 cigarettes/day | 1 803 (3.1) | 3 550 (3.0) | 4 393 (2.9) | 4 437 (3.3) |
| Current ≥15 cigarettes/day | 1 684 (2.9) | 4 237 (3.6) | 5 974 (3.9) | 7 558 (5.5) |
| Current, amount unknown | 1 857 (3.2) | 3 812 (3.2) | 4 819 (3.1) | 5 223 (3.8) |
| Unknown | 243 (0.4) | 430 (0.4) | 474 (0.3) | 441 (0.3) |
| Physical activity level, n (%) |  |  |  |  |
| Low <10 excess METs | 16 979 (29.3) | 37 697 (31.6) | 48 730 (31.7) | 43 733 (32.0) |
| Moderate 10-<50 excess METs | 29 462 (50.8) | 58 785 (49.3) | 75 725 (49.3) | 64 544 (47.3) |
| High ≥ 50 excess METs | 9 533 (16.4) | 18 476 (15.5) | 23 774 (15.5) | 22 995 (16.8) |
| Unknown | 1 982 (3.4) | 4 321 (3.6) | 5 448 (3.5) | 5 200 (3.8) |
| Alcohol intake, n (%) |  |  |  |  |
| Non-drinkers | 8 166 (14.1) | 9 527 (8.0) | 9 882 (6.4) | 9 200 (6.7) |
| <1g/d | 8 580 (14.8) | 14 177 (11.9) | 16 375 (10.7) | 12 789 (9.4) |
| 1-<10g/d | 20 008 (34.5) | 40 727 (34.1) | 49 509 (32.2) | 36 434 (26.7) |
| 10-<20g/d | 11 270 (19.4) | 26 702 (22.4) | 34 931 (22.7) | 28 544 (20.9) |
| 20+g/d | 9 598 (16.6) | 27 546 (23.1) | 42 325 (27.5) | 48 871 (35.8) |
| Unknown | 334 (0.6) | 600 (0.5) | 655 (0.4) | 634 (0.5) |
| **Diet** |  |  |  |  |
| Fruit & vegetable intake (s/day), mean (SD) | 5.34 (3.07) | 4.71 (2.49) | 4.64 (2.47) | 4.46 (2.57) |
| Fruit & vegetable intake categories, n (%) |  |  |  |  |
| <3 servings/day | 8 520 (14.7) | 21 856 (18.3) | 29 475 (19.2) | 31 281 (22.9) |
| 3-<4 servings/day | 8 422 (14.5) | 21 833 (18.3) | 28 819 (18.8) | 26 781 (19.6) |
| 4-<6 servings/day | 19 438 (33.5) | 42 463 (35.6) | 54 977 (35.8) | 45 180 (33.1) |
| ≥6 servings/day | 20 303 (35.0) | 30 966 (26.0) | 37 968 (24.7) | 30 689 (22.5) |
| Unknown | 1 273 (2.2) | 2 161 (1.8) | 2 438 (1.6) | 2 541 (1.9) |
| Cereal fibre intake (g/day), mean (SD) | 4.64 (3.07) | 4.52 (2.90) | 4.52 (2.89) | 4.44 (2.96) |
| Cereal/Bread fibre sex-spec Quintiles, n (%) |  |  |  |  |
| Q1 (mean W: 1.1 g/day, M: 1.3 g/day) | 10 703 (18.5) | 22 408 (18.8) | 28 854 (18.8) | 29 608 (21.7) |
| Q2 (mean W: 2.5 g/day, M: 2.8 g/day) | 10 646 (18.4) | 23 500 (19.7) | 30 808 (20.0) | 27 467 (20.1) |
| Q3 (mean W: 3.7 g/day, M: 4.3 g/day) | 10 941 (18.9) | 23 566 (19.8) | 30 952 (20.1) | 26 487 (19.4) |
| Q4 (mean W: 5.3 g/day, M: 6.2 g/day) | 11 769 (20.3) | 23 720 (19.9) | 30 392 (19.8) | 24 854 (18.2) |
| Q5 (mean W: 8.4 g/day, M: 9.9 g/day) | 12 726 (22.0) | 23 901 (20.0) | 29 894 (19.5) | 25 190 (18.5) |
| Unknown | 1 171 (2.0) | 2 184 (1.8) | 2 777 (1.8) | 2 866 (2.1) |
| Oily fish, n (%) |  |  |  |  |
| 0 times/week | 13 755 (23.7) | 9 379 (7.9) | 12 967 (8.4) | 15 140 (11.1) |
| <1 times/week | 14 581 (25.2) | 39 271 (32.9) | 52 082 (33.9) | 48 996 (35.9) |
| 1 time/week | 16 276 (28.1) | 48 097 (40.3) | 61 127 (39.8) | 50 195 (36.8) |
| >2 times/week | 13 090 (22.6) | 22 077 (18.5) | 26 935 (17.5) | 21 488 (15.7) |
| Unknown | 254 (0.4) | 455 (0.4) | 566 (0.4) | 653 (0.5) |
| Non-oily fish, n (%) |  |  |  |  |
| <1 time/week | 27 289 (47.1) | 39 787 (33.4) | 47 472 (30.9) | 43 181 (31.6) |
| 1 times/week | 20 192 (34.8) | 61 857 (51.9) | 80 833 (52.6) | 68 784 (50.4) |
| >2 times/week | 10 207 (17.6) | 17 298 (14.5) | 24 945 (16.2) | 24 017 (17.6) |
| Unknown | 268 (0.5) | 337 (0.3) | 427 (0.3) | 490 (0.4) |
| Unprocessed red meat, n (%) |  |  |  |  |
| <1 times/week | 32 914 (56.8) | 11 075 (9.3) | 2 704 (1.8) | 846 (0.6) |
| 1 times/week | 24 719 (42.7) | 58 492 (49.0) | 68 743 (44.7) | 32 302 (23.7) |
| 2-3 times/week | 323 (0.6) | 49 581 (41.6) | 72 251 (47.0) | 51 458 (37.7) |
| >4 times/week | 0 (0.0) | 131 (0.1) | 9 979 (6.5) | 51 866 (38.0) |
| Processed meat, n (%) |  |  |  |  |
| 0 times/week | 29 598 (51.1) | 9 252 (7.8) | 3 313 (2.2) | 1 518 (1.1) |
| <1 times/week | 24 432 (42.2) | 55 459 (46.5) | 49 108 (32.0) | 13 155 (9.6) |
| 1 times/week | 3 926 (6.8) | 51 485 (43.2) | 58 968 (38.4) | 21 970 (16.1) |
| >2 times/week | 0 (0.0) | 3 083 (2.6) | 42 288 (27.5) | 99 829 (73.1) |
| Poultry meat, n (%) |  |  |  |  |
| 0-1 times/week | 42 695 (73.7) | 15 014 (12.6) | 11 132 (7.2) | 5 242 (3.8) |
| 2 times/week | 15 261 (26.3) | 83 987 (70.4) | 47 031 (30.6) | 20 963 (15.4) |
| >3 times/week | 0 (0.0) | 20 278 (17.0) | 95 514 (62.2) | 110 267 (80.8) |
| **Women factors** |  |  |  |  |
| Menopausal status, n (%) |  |  |  |  |
| Premenopausal | 10 420 (26.3) | 14 862 (20.6) | 19 701 (23.5) | 15 146 (26.6) |
| Postmenopausal | 27 046 (68.3) | 53 651 (74.5) | 59 389 (70.9) | 38 040 (66.9) |
| Unknown | 2 108 (5.3) | 3 502 (4.9) | 4 701 (5.6) | 3 681 (6.5) |
| Parity, n (%) |  |  |  |  |
| 0 births | 10 803 (27.3) | 13 777 (19.1) | 13 677 (16.3) | 8 840 (15.5) |
| 1 - 2 births | 20 358 (51.4) | 41 238 (57.3) | 49 381 (58.9) | 33 109 (58.2) |
| ≥3 births | 8 350 (21.1) | 16 935 (23.5) | 20 677 (24.7) | 14 855 (26.1) |
| Unknown | 63 (0.2) | 65 (0.1) | 56 (0.1) | 63 (0.1) |
| Hormone replacement therapy use (HRT), n (%) |  |  |  |  |
| Never | 26 413 (66.7) | 43 236 (60.0) | 50 904 (60.8) | 35 686 (62.8) |
| Past | 10 716 (27.1) | 24 118 (33.5) | 27 378 (32.7) | 17 421 (30.6) |
| Current | 2 268 (5.7) | 4 441 (6.2) | 5 276 (6.3) | 3 490 (6.1) |
| Unknown | 177 (0.4) | 220 (0.3) | 233 (0.3) | 270 (0.5) |
| Oral contraceptive pill use (OCT), n (%) |  |  |  |  |
| Never | 8 013 (20.2) | 13 890 (19.3) | 14 590 (17.4) | 9 991 (17.6) |
| Past | 30 592 (77.3) | 56 729 (78.8) | 67 420 (80.5) | 45 478 (80.0) |
| Current | 795 (2.0) | 1 237 (1.7) | 1 588 (1.9) | 1 188 (2.1) |
| Unknown | 174 (0.4) | 159 (0.2) | 193 (0.2) | 210 (0.4) |
| The x^2^ test was used to compare the distribution between meat intakes for all categorical variables. Analysis of variance (ANOVA) was used to compare the means between meat intakes. The *P*-heterogeneity between meat intakes was <0.001 for all variables. All dietary data come from the touchscreen questionnaire. **^a^**See Additional file 1: Fig. 1 BMI: Body mass index HRT: hormone replacement therapy, OCP: oral contraceptive pill use, NVQ: national vocational qualification, s/day: servings/day. | | | | |

| Additional file 1:Table 3. Baseline characteristics of participants by unprocessed red meat intake in UK Biobank (n=468 328^a^). | | | | |
| --- | --- | --- | --- | --- |
| Characteristic | <1 time/week | 1 time/week | 2-3 times/week | > 4times/week |
| Mean (SD) or n (%) | N=47 718 | N=184 612 | N=173 879 | N=62 119 |
| **Sociodemographic** |  |  |  |  |
| Sex, n (%) |  |  |  |  |
| Women | 31 959 (67.0) | 103 127 (55.9) | 88 309 (50.8) | 29 329 (47.2) |
| Men | 15 759 (33.0) | 81 485 (44.1) | 85 570 (49.2) | 32 790 (52.8) |
| Age (years), mean (SD) | 54.7 (8.2) | 56.1 (8.1) | 56.8 (8.0) | 56.7 (8.1) |
| Age (years), n (%) |  |  |  |  |
| 37-44 | 7 157 (15.0) | 20 104 (10.9) | 16 533 (9.5) | 6 127 (9.9) |
| 45-49 | 7 787 (16.3) | 25 719 (13.9) | 21 794 (12.5) | 7 983 (12.9) |
| 50-54 | 7 976 (16.7) | 29 324 (15.9) | 25 738 (14.8) | 9 283 (14.9) |
| 55-59 | 8 612 (18.0) | 33 864 (18.3) | 31 706 (18.2) | 10 928 (17.6) |
| 60-64 | 9 681 (20.3) | 43 087 (23.3) | 43 600 (25.1) | 15 412 (24.8) |
| 65- 73 | 6 505 (13.6) | 32 514 (17.6) | 34 508 (19.8) | 12 386 (19.9) |
| Race, n (%) |  |  |  |  |
| White | 42 270 (88.6) | 175 499 (95.1) | 166 553 (95.8) | 57 560 (92.7) |
| Asian or Asian British | 3 516 (7.4) | 3 378 (1.8) | 2 366 (1.4) | 1 357 (2.2) |
| Black or Black British | 798 (1.7) | 2 672 (1.4) | 2 154 (1.2) | 1 667 (2.7) |
| Mixed Race/Other | 915 (1.9) | 2 476 (1.3) | 2 269 (1.3) | 1 278 (2.1) |
| Unknown | 219 (0.5) | 587 (0.3) | 537 (0.3) | 257 (0.4) |
| Townsend deprivation, n (%) |  |  |  |  |
| Most affluent (mean -4.7) | 7 520 (15.8) | 36 902 (20.0) | 37 345 (21.5) | 12 628 (20.3) |
| 2 (mean -3.3) | 8 050 (16.9) | 37 187 (20.1) | 36 367 (20.9) | 12 111 (19.5) |
| 3 (mean -2.1) | 9 102 (19.1) | 37 438 (20.3) | 35 175 (20.2) | 12 101 (19.5) |
| 4 (mean -0.1) | 10 968 (23.0) | 36 955 (20.0) | 33 724 (19.4) | 11 899 (19.2) |
| Most deprived (mean 3.8) | 12 007 (25.2) | 35 900 (19.4) | 31 067 (17.9) | 13 299 (21.4) |
| Unknown | 71 (0.1) | 230 (0.1) | 201 (0.1) | 81 (0.1) |
| Qualification, n (%) |  |  |  |  |
| College/university degree/NVQ | 29 966 (62.8) | 110 523 (59.9) | 102 705 (59.1) | 36 375 (58.6) |
| National examination at ages 17‑18 | 2 673 (5.6) | 10 112 (5.5) | 9 345 (5.4) | 3 504 (5.6) |
| National examination at age 16 | 7 296 (15.3) | 32 161 (17.4) | 28 742 (16.5) | 9 712 (15.6) |
| Other/unknown | 7 783 (16.3) | 31 816 (17.2) | 33 087 (19.0) | 12 528 (20.2) |
| Employment, n (%) |  |  |  |  |
| In paid employment | 30 053 (63.0) | 111 151 (60.2) | 98 554 (56.7) | 33 842 (54.5) |
| Pension | 10 846 (22.7) | 52 387 (28.4) | 55 394 (31.9) | 19 855 (32.0) |
| Not in paid employment | 6 210 (13.0) | 19 486 (10.6) | 18 511 (10.6) | 7 787 (12.5) |
| Unknown | 609 (1.3) | 1 588 (0.9) | 1 420 (0.8) | 635 (1.0) |
| **Physical measurements** |  |  |  |  |
| BMI (kg/m^2^), mean (SD) | 26.2 (4.8) | 27.3 (4.7) | 27.7 (4.7) | 28.1 (4.9) |
| BMI (kg/m^2^), fifths, n (%) |  |  |  |  |
| Q1 (mean W: 21 kg/m^2^, M: 23 kg/m^2^) | 14 589 (30.6) | 39 294 (21.3) | 32 330 (18.6) | 10 590 (17.0) |
| Q2 (mean W: 24 kg/m^2^, M: 26 kg/m^2^) | 9 857 (20.7) | 37 275 (20.2) | 33 284 (19.1) | 10 979 (17.7) |
| Q3 (mean W: 26 kg/m^2^, M: 27 kg/m^2^) | 8 859 (18.6) | 37 707 (20.4) | 36 374 (20.9) | 12 374 (19.9) |
| Q4 (mean W: 29 kg/m^2^, M: 29 kg/m^2^) | 7 417 (15.5) | 35 056 (19.0) | 34 953 (20.1) | 13 005 (20.9) |
| Q5 (mean W: 35 kg/m^2^, M: 34 kg/m^2^) | 6 687 (14.0) | 34 489 (18.7) | 36 145 (20.8) | 14 832 (23.9) |
| Unknown | 309 (0.6) | 791 (0.4) | 793 (0.5) | 339 (0.5) |
| **Lifestyle** |  |  |  |  |
| Smoking, n (%) |  |  |  |  |
| Never | 28 307 (59.3) | 102 383 (55.5) | 93 739 (53.9) | 32 099 (51.7) |
| Former | 15 055 (31.5) | 63 155 (34.2) | 60 965 (35.1) | 21 513 (34.6) |
| Current <15 cigarettes/day | 1 440 (3.0) | 5 595 (3.0) | 5 056 (2.9) | 2 136 (3.4) |
| Current ≥15 cigarettes/day | 1 375 (2.9) | 6 900 (3.7) | 7 553 (4.3) | 3 692 (5.9) |
| Current, amount unknown | 1 353 (2.8) | 5 987 (3.2) | 5 988 (3.4) | 2 424 (3.9) |
| Unknown | 188 (0.4) | 592 (0.3) | 578 (0.3) | 255 (0.4) |
| Physical activity level, n (%) |  |  |  |  |
| Low <10 excess METs | 13 788 (28.9) | 58 482 (31.7) | 55 205 (31.7) | 20 033 (32.2) |
| Moderate 10-<50 excess METs | 23 907 (50.1) | 90 807 (49.2) | 85 002 (48.9) | 29 093 (46.8) |
| High ≥ 50 excess METs | 8 349 (17.5) | 28 734 (15.6) | 27 350 (15.7) | 10 420 (16.8) |
| Unknown | 1 674 (3.5) | 6 589 (3.6) | 6 322 (3.6) | 2 573 (4.1) |
| Alcohol intake, n (%) |  |  |  |  |
| Non-drinkers | 7 795 (16.3) | 13 817 (7.5) | 10 971 (6.3) | 4 427 (7.1) |
| <1g/d | 7 273 (15.2) | 22 273 (12.1) | 16 572 (9.5) | 6 008 (9.7) |
| 1-<10g/d | 15 695 (32.9) | 62 535 (33.9) | 52 016 (29.9) | 16 660 (26.8) |
| 10-<20g/d | 8 618 (18.1) | 40 428 (21.9) | 39 594 (22.8) | 12 903 (20.8) |
| 20+g/d | 8 062 (16.9) | 44 602 (24.2) | 54 008 (31.1) | 21 799 (35.1) |
| Unknown | 275 (0.6) | 957 (0.5) | 718 (0.4) | 322 (0.5) |
| **Diet** |  |  |  |  |
| Fruit & vegetable intake (s/day), mean (SD) | 5.35 (3.16) | 4.68 (2.55) | 4.58 (2.45) | 4.54 (2.61) |
| Fruit & vegetable intake categories, n (%) |  |  |  |  |
| <3 servings/day | 7 335 (15.4) | 36 111 (19.6) | 34 381 (19.8) | 13 498 (21.7) |
| 3-<4 servings/day | 6 863 (14.4) | 33 216 (18.0) | 33 761 (19.4) | 12 119 (19.5) |
| 4-<6 servings/day | 15 625 (32.7) | 64 178 (34.8) | 61 671 (35.5) | 20 759 (33.4) |
| ≥6 servings/day | 16 835 (35.3) | 47 604 (25.8) | 41 201 (23.7) | 14 466 (23.3) |
| Unknown | 1 060 (2.2) | 3 503 (1.9) | 2 865 (1.6) | 1 277 (2.1) |
| Cereal fibre intake (g/day), mean (SD) | 4.69 (3.13) | 4.58 (2.94) | 4.48 (2.87) | 4.27 (2.92) |
| Cereal/Bread fibre sex-spec Quintiles, n (%) |  |  |  |  |
| Q1 (mean W: 1.1 g/day, M: 1.3 g/day) | 8 815 (18.5) | 34 270 (18.6) | 34 277 (19.7) | 14 469 (23.3) |
| Q2 (mean W: 2.5 g/day, M: 2.8 g/day) | 8 576 (18.0) | 36 497 (19.8) | 34 796 (20.0) | 12 691 (20.4) |
| Q3 (mean W: 3.7 g/day, M: 4.3 g/day) | 8 956 (18.8) | 36 439 (19.7) | 34 787 (20.0) | 11 889 (19.1) |
| Q4 (mean W: 5.3 g/day, M: 6.2 g/day) | 9 581 (20.1) | 36 633 (19.8) | 33 799 (19.4) | 10 820 (17.4) |
| Q5 (mean W: 8.4 g/day, M: 9.9 g/day) | 10 749 (22.5) | 37 299 (20.2) | 33 000 (19.0) | 10 758 (17.3) |
| Unknown | 1 041 (2.2) | 3 474 (1.9) | 3 220 (1.9) | 1 492 (2.4) |
| Oily fish, n (%) |  |  |  |  |
| 0 times/week | 14 082 (29.5) | 16 868 (9.1) | 14 137 (8.1) | 6 334 (10.2) |
| <1 times/week | 9 907 (20.8) | 63 735 (34.5) | 58 601 (33.7) | 22 920 (36.9) |
| 1 time/week | 12 284 (25.7) | 68 398 (37.0) | 72 503 (41.7) | 22 766 (36.6) |
| >2 times/week | 11 198 (23.5) | 34 830 (18.9) | 27 951 (16.1) | 9 742 (15.7) |
| Unknown | 247 (0.5) | 781 (0.4) | 687 (0.4) | 357 (0.6) |
| Non-oily fish, n (%) |  |  |  |  |
| <1 time/week | 21 488 (45.0) | 63 252 (34.3) | 52 348 (30.1) | 20 971 (33.8) |
| 1 times/week | 15 977 (33.5) | 90 337 (48.9) | 95 075 (54.7) | 30 610 (49.3) |
| >2 times/week | 10 010 (21.0) | 30 394 (16.5) | 25 940 (14.9) | 10 237 (16.5) |
| Unknown | 243 (0.5) | 629 (0.3) | 516 (0.3) | 301 (0.5) |
| Processed meat, n (%) |  |  |  |  |
| 0 times/week | 27 539 (57.7) | 9 655 (5.2) | 4 917 (2.8) | 1 613 (2.6) |
| <1 times/week | 10 547 (22.1) | 69 708 (37.8) | 47 383 (27.3) | 14 594 (23.5) |
| 1 times/week | 5 169 (10.8) | 53 125 (28.8) | 59 962 (34.5) | 18 210 (29.3) |
| >2 times/week | 4 358 (9.1) | 51 905 (28.1) | 61 452 (35.3) | 27 604 (44.4) |
| Unknown | 105 (0.2) | 219 (0.1) | 165 (0.1) | 98 (0.2) |
| Poultry meat, n (%) |  |  |  |  |
| 0-1 times/week | 26 990 (56.6) | 28 809 (15.6) | 12 790 (7.4) | 5 617 (9.0) |
| 2 times/week | 8 114 (17.0) | 67 608 (36.6) | 72 906 (41.9) | 18 818 (30.3) |
| >3 times/week | 12 519 (26.2) | 88 048 (47.7) | 88 069 (50.6) | 37 630 (60.6) |
| Unknown | 95 (0.2) | 147 (0.1) | 114 (0.1) | 54 (0.1) |
| **Women factors** |  |  |  |  |
| Menopausal status, n (%) |  |  |  |  |
| Premenopausal | 9 585 (30.0) | 24 862 (24.1) | 19 337 (21.9) | 6 437 (21.9) |
| Postmenopausal | 20 536 (64.3) | 72 369 (70.2) | 64 312 (72.8) | 21 245 (72.4) |
| Unknown | 1 838 (5.8) | 5 896 (5.7) | 4 660 (5.3) | 1 647 (5.6) |
| Parity, n (%) |  |  |  |  |
| 0 births | 8 414 (26.3) | 20 625 (20.0) | 13 677 (15.5) | 4 462 (15.2) |
| 1 - 2 births | 16 737 (52.4) | 58 384 (56.6) | 52 298 (59.2) | 16 902 (57.6) |
| ≥3 births | 6 752 (21.1) | 24 022 (23.3) | 22 264 (25.2) | 7 929 (27.0) |
| Unknown | 56 (0.2) | 96 (0.1) | 70 (0.1) | 36 (0.1) |
| Hormone replacement therapy use (HRT), n (%) |  |  |  |  |
| Never | 22 097 (69.1) | 64 168 (62.2) | 52 758 (59.7) | 17 500 (59.7) |
| Past | 7 936 (24.8) | 32 321 (31.3) | 29 707 (33.6) | 9 826 (33.5) |
| Current | 1 769 (5.5) | 6 308 (6.1) | 5 562 (6.3) | 1 850 (6.3) |
| Unknown | 157 (0.5) | 330 (0.3) | 282 (0.3) | 153 (0.5) |
| Oral contraceptive pill use (OCT), n (%) |  |  |  |  |
| Never | 6 634 (20.8) | 18 496 (17.9) | 15 948 (18.1) | 5 563 (19.0) |
| Past | 24 415 (76.4) | 82 384 (79.9) | 70 570 (79.9) | 23 143 (78.9) |
| Current | 746 (2.3) | 1 994 (1.9) | 1 582 (1.8) | 490 (1.7) |
| Unknown | 164 (0.5) | 253 (0.2) | 209 (0.2) | 133 (0.5) |
| The x^2^ test was used to compare the distribution between meat intakes for all categorical variables. Analysis of variance (ANOVA) was used to compare the means between meat intakes. The *P*-heterogeneity between meat intakes was <0.001 for all variables. All dietary data come from the touchscreen questionnaire. **^a^**Additional file 1: Fig. 1 BMI: Body mass index HRT: hormone replacement therapy, OCP: oral contraceptive pill use, NVQ: national vocational qualification, s/day: servings/day. | | | | |

| Additional file 1: Table 4. Baseline characteristics of participants by processed meat intake in UK Biobank (n=472 844^a^). | | | | |
| --- | --- | --- | --- | --- |
| Characteristic | 0 times/week | <1 time/week | 1 time/week | >2 times/week |
| Mean (SD) or n (%) | N=44 036 | N=143 514 | N=138 078 | N=147 216 |
| **Sociodemographic** |  |  |  |  |
| Sex, n (%) |  |  |  |  |
| Women | 32 191 (73.1) | 96 947 (67.6) | 73 134 (53.0) | 52 616 (35.7) |
| Men | 11 845 (26.9) | 46 567 (32.4) | 64 944 (47.0) | 94 600 (64.3) |
| Age (years), mean (SD) | 55.7 (8.1) | 56.7 (7.9) | 56.5 (8.1) | 56.0 (8.3) |
| Age (years), n (%) |  |  |  |  |
| 37-44 | 5 296 (12.0) | 13 231 (9.2) | 14 388 (10.4) | 17 517 (11.9) |
| 45-49 | 6 238 (14.2) | 18 136 (12.6) | 18 217 (13.2) | 21 335 (14.5) |
| 50-54 | 7 193 (16.3) | 22 146 (15.4) | 20 964 (15.2) | 22 658 (15.4) |
| 55-59 | 8 468 (19.2) | 27 378 (19.1) | 24 740 (17.9) | 25 246 (17.1) |
| 60-64 | 9 911 (22.5) | 36 215 (25.2) | 33 119 (24.0) | 33 455 (22.7) |
| 65- 73 | 6 930 (15.7) | 26 408 (18.4) | 26 650 (19.3) | 27 005 (18.3) |
| Race, n (%) |  |  |  |  |
| White | 38 207 (86.8) | 134 161 (93.5) | 132 037 (95.6) | 141 080 (95.8) |
| Asian or Asian British | 3 262 (7.4) | 3 381 (2.4) | 2 139 (1.5) | 2 160 (1.5) |
| Black or Black British | 1 153 (2.6) | 2 933 (2.0) | 1 772 (1.3) | 1 763 (1.2) |
| Mixed race/Other | 1 178 (2.7) | 2 566 (1.8) | 1 675 (1.2) | 1 726 (1.2) |
| Unknown | 236 (0.5) | 473 (0.3) | 455 (0.3) | 487 (0.3) |
| Townsend deprivation, n (%) |  |  |  |  |
| Most affluent (mean -4.7) | 7 325 (16.6) | 29 721 (20.7) | 29 264 (21.2) | 28 556 (19.4) |
| 2 (mean -3.3) | 7 570 (17.2) | 29 409 (20.5) | 28 618 (20.7) | 28 657 (19.5) |
| 3 (mean -2.1) | 8 313 (18.9) | 28 886 (20.1) | 28 255 (20.5) | 29 055 (19.7) |
| 4 (mean -0.1) | 9 903 (22.5) | 28 470 (19.8) | 26 816 (19.4) | 29 356 (19.9) |
| Most deprived (mean 3.8) | 10 870 (24.7) | 26 854 (18.7) | 24 937 (18.1) | 31 419 (21.3) |
| Unknown | 55 (0.1) | 174 (0.1) | 188 (0.1) | 173 (0.1) |
| Qualification, n (%) |  |  |  |  |
| College/university degree/NVQ | 28 394 (64.5) | 86 829 (60.5) | 80 169 (58.1) | 85 692 (58.2) |
| National examination at ages 17‑18 | 2 486 (5.6) | 7 999 (5.6) | 7 412 (5.4) | 7 871 (5.3) |
| National examination at age 16 | 6 007 (13.6) | 24 635 (17.2) | 23 144 (16.8) | 24 753 (16.8) |
| Other/unknown | 7 149 (16.2) | 24 051 (16.8) | 27 353 (19.8) | 28 900 (19.6) |
| Employment, n (%) |  |  |  |  |
| In paid employment | 26 487 (60.1) | 82 246 (57.3) | 79 689 (57.7) | 87 204 (59.2) |
| Pension | 11 404 (25.9) | 44 697 (31.1) | 42 509 (30.8) | 41 251 (28.0) |
| Not in paid employment | 5 520 (12.5) | 15 261 (10.6) | 14 705 (10.6) | 17 427 (11.8) |
| Unknown | 625 (1.4) | 1 310 (0.9) | 1 175 (0.9) | 1 334 (0.9) |
| **Physical measurements** |  |  |  |  |
| BMI (kg/m^2^), mean (SD) | 26.0 (4.6) | 27.1 (4.7) | 27.6 (4.7) | 28.1 (4.9) |
| BMI (kg/m^2^), fifths, n (%) |  |  |  |  |
| Q1 (mean W: 21 kg/m^2^, M: 23 kg/m^2^) | 13 687 (31.1) | 30 563 (21.3) | 26 335 (19.1) | 26 985 (18.3) |
| Q2 (mean W: 24 kg/m^2^, M: 26 kg/m^2^) | 9 260 (21.0) | 29 588 (20.6) | 26 887 (19.5) | 26 394 (17.9) |
| Q3 (mean W: 26 kg/m^2^, M: 27 kg/m^2^) | 8 258 (18.8) | 30 122 (21.0) | 28 779 (20.8) | 28 955 (19.7) |
| Q4 (mean W: 29 kg/m^2^, M: 29 kg/m^2^) | 6 830 (15.5) | 27 305 (19.0) | 27 684 (20.0) | 29 555 (20.1) |
| Q5 (mean W: 35 kg/m^2^, M: 34 kg/m^2^) | 5 695 (12.9) | 25 308 (17.6) | 27 806 (20.1) | 34 555 (23.5) |
| Unknown | 306 (0.7) | 628 (0.4) | 587 (0.4) | 772 (0.5) |
| **Lifestyle** |  |  |  |  |
| Smoking, n (%) |  |  |  |  |
| Never | 26 093 (59.3) | 81 556 (56.8) | 75 675 (54.8) | 75 486 (51.3) |
| Former | 14 197 (32.2) | 48 847 (34.0) | 47 708 (34.6) | 51 275 (34.8) |
| Current <15 cigarettes/day | 1 291 (2.9) | 4 043 (2.8) | 4 140 (3.0) | 4 945 (3.4) |
| Current ≥15 cigarettes/day | 1 026 (2.3) | 4 353 (3.0) | 5 316 (3.8) | 9 186 (6.2) |
| Current, amount unknown | 1 232 (2.8) | 4 242 (3.0) | 4 672 (3.4) | 5 836 (4.0) |
| Unknown | 197 (0.4) | 473 (0.3) | 567 (0.4) | 488 (0.3) |
| Physical activity level, n (%) |  |  |  |  |
| Low <10 excess METs | 12 161 (27.6) | 44 583 (31.1) | 44 324 (32.1) | 48 113 (32.7) |
| Moderate 10-<50 excess METs | 22 352 (50.8) | 71 630 (49.9) | 67 254 (48.7) | 69 084 (46.9) |
| High ≥ 50 excess METs | 8 017 (18.2) | 22 265 (15.5) | 21 065 (15.3) | 24 042 (16.3) |
| Unknown | 1 506 (3.4) | 5 036 (3.5) | 5 435 (3.9) | 5 977 (4.1) |
| Alcohol intake, n (%) |  |  |  |  |
| Non-drinkers | 7 672 (17.4) | 11 249 (7.8) | 9 198 (6.7) | 9 628 (6.5) |
| <1g/d | 6 622 (15.0) | 18 280 (12.7) | 14 409 (10.4) | 13 710 (9.3) |
| 1-<10g/d | 14 607 (33.2) | 51 359 (35.8) | 43 445 (31.5) | 38 747 (26.3) |
| 10-<20g/d | 8 196 (18.6) | 32 108 (22.4) | 31 803 (23.0) | 30 091 (20.4) |
| 20+g/d | 6 713 (15.2) | 29 773 (20.7) | 38 557 (27.9) | 54 283 (36.9) |
| Unknown | 226 (0.5) | 745 (0.5) | 666 (0.5) | 757 (0.5) |
| **Diet** |  |  |  |  |
| Fruit & vegetable intake (s/day), mean (SD) | 5.82 (3.28) | 4.99 (2.55) | 4.53 (2.42) | 4.20 (2.45) |
| Fruit & vegetable intake categories, n (%) |  |  |  |  |
| <3 servings/day | 4 577 (10.4) | 21 213 (14.8) | 27 796 (20.1) | 38 779 (26.3) |
| 3-<4 servings/day | 5 541 (12.6) | 23 960 (16.7) | 27 087 (19.6) | 29 943 (20.3) |
| 4-<6 servings/day | 14 636 (33.2) | 52 829 (36.8) | 48 874 (35.4) | 46 949 (31.9) |
| ≥6 servings/day | 18 426 (41.8) | 42 933 (29.9) | 31 653 (22.9) | 27 987 (19.0) |
| Unknown | 856 (1.9) | 2 579 (1.8) | 2 668 (1.9) | 3 558 (2.4) |
| Cereal fibre intake (g/day), mean (SD) | 4.56 (3.08) | 4.43 (2.87) | 4.52 (2.88) | 4.54 (3.00) |
| Cereal/Bread fibre sex-spec Quintiles, n (%) |  |  |  |  |
| Q1 (mean W: 1.1 g/day, M: 1.3 g/day) | 8 343 (18.9) | 27 287 (19.0) | 26 375 (19.1) | 30 920 (21.0) |
| Q2 (mean W: 2.5 g/day, M: 2.8 g/day) | 8 256 (18.7) | 28 530 (19.9) | 27 193 (19.7) | 29 430 (20.0) |
| Q3 (mean W: 3.7 g/day, M: 4.3 g/day) | 8 194 (18.6) | 28 373 (19.8) | 27 816 (20.1) | 28 431 (19.3) |
| Q4 (mean W: 5.3 g/day, M: 6.2 g/day) | 8 722 (19.8) | 28 444 (19.8) | 27 182 (19.7) | 27 043 (18.4) |
| Q5 (mean W: 8.4 g/day, M: 9.9 g/day) | 9 630 (21.9) | 28 095 (19.6) | 26 613 (19.3) | 28 012 (19.0) |
| Unknown | 891 (2.0) | 2 785 (1.9) | 2 899 (2.1) | 3 380 (2.3) |
| Oily fish, n (%) |  |  |  |  |
| 0 times/week | 11 758 (26.7) | 10 529 (7.3) | 12 005 (8.7) | 17 780 (12.1) |
| <1 times/week | 7 773 (17.7) | 48 392 (33.7) | 46 247 (33.5) | 53 924 (36.6) |
| 1 time/week | 12 431 (28.2) | 55 197 (38.5) | 56 533 (40.9) | 53 225 (36.2) |
| >2 times/week | 11 896 (27.0) | 28 741 (20.0) | 22 513 (16.3) | 21 324 (14.5) |
| Unknown | 178 (0.4) | 655 (0.5) | 780 (0.6) | 963 (0.7) |
| Non-oily fish, n (%) |  |  |  |  |
| <1 time/week | 19 470 (44.2) | 51 756 (36.1) | 41 342 (29.9) | 46 943 (31.9) |
| 1 times/week | 15 168 (34.4) | 67 714 (47.2) | 75 684 (54.8) | 75 395 (51.2) |
| >2 times/week | 9 167 (20.8) | 23 426 (16.3) | 20 487 (14.8) | 24 141 (16.4) |
| Unknown | 231 (0.5) | 618 (0.4) | 565 (0.4) | 737 (0.5) |
| Unprocessed red meat, n (%) |  |  |  |  |
| <1 times/week | 27 539 (62.5) | 10 547 (7.3) | 5 169 (3.7) | 4 358 (3.0) |
| 1 times/week | 9 655 (21.9) | 69 708 (48.6) | 53 125 (38.5) | 51 905 (35.3) |
| 2-3 times/week | 4 917 (11.2) | 47 383 (33.0) | 59 962 (43.4) | 61 452 (41.7) |
| >4 times/week | 1 613 (3.7) | 14 594 (10.2) | 18 210 (13.2) | 27 604 (18.8) |
| Unknown | 312 (0.7) | 1 282 (0.9) | 1 612 (1.2) | 1 897 (1.3) |
| Poultry meat, n (%) |  |  |  |  |
| 0-1 times/week | 24 778 (56.3) | 21 748 (15.2) | 12 657 (9.2) | 15 650 (10.6) |
| 2 times/week | 7 762 (17.6) | 54 281 (37.8) | 55 748 (40.4) | 51 356 (34.9) |
| >3 times/week | 11 430 (26.0) | 67 327 (46.9) | 69 459 (50.3) | 79 960 (54.3) |
| Unknown | 66 (0.1) | 158 (0.1) | 214 (0.2) | 250 (0.2) |
| **Women factors** |  |  |  |  |
| Menopausal status, n (%) |  |  |  |  |
| Premenopausal | 7 975 (24.8) | 21 066 (21.7) | 17 599 (24.1) | 14 011 (26.6) |
| Postmenopausal | 22 551 (70.1) | 70 660 (72.9) | 51 469 (70.4) | 35 383 (67.2) |
| Unknown | 1 665 (5.2) | 5 221 (5.4) | 4 066 (5.6) | 3 222 (6.1) |
| Parity, n (%) |  |  |  |  |
| 0 births | 8 050 (25.0) | 18 760 (19.4) | 11 834 (16.2) | 8 886 (16.9) |
| 1 - 2 births | 16 759 (52.1) | 55 312 (57.1) | 43 099 (58.9) | 30 270 (57.5) |
| ≥3 births | 7 323 (22.7) | 22 784 (23.5) | 18 141 (24.8) | 13 402 (25.5) |
| Unknown | 59 (0.2) | 91 (0.1) | 60 (0.1) | 58 (0.1) |
| Hormone replacement therapy use (HRT), n (%) |  |  |  |  |
| Never | 21 050 (65.4) | 58 430 (60.3) | 45 142 (61.7) | 33 215 (63.1) |
| Past | 9 045 (28.1) | 32 022 (33.0) | 23 352 (31.9) | 16 077 (30.6) |
| Current | 1 922 (6.0) | 6 204 (6.4) | 4 402 (6.0) | 3 062 (5.8) |
| Unknown | 174 (0.5) | 291 (0.3) | 238 (0.3) | 262 (0.5) |
| Oral contraceptive pill use (OCT), n (%) |  |  |  |  |
| Never | 6 984 (21.7) | 17 217 (17.8) | 13 323 (18.2) | 9 800 (18.6) |
| Past | 24 446 (75.9) | 77 775 (80.2) | 58 257 (79.7) | 41 430 (78.7) |
| Current | 596 (1.9) | 1 709 (1.8) | 1 369 (1.9) | 1 170 (2.2) |
| Unknown | 165 (0.5) | 246 (0.3) | 185 (0.3) | 216 (0.4) |
| The x^2^ test was used to compare the distribution between meat intakes for all categorical variables. Analysis of variance (ANOVA) was used to compare the means between meat intakes. The *P*-heterogeneity between meat intakes was <0.001 for all variables. All dietary data come from the touchscreen questionnaire. **^a^**Additional file 1: Fig. 1 BMI: Body mass index HRT: hormone replacement therapy, OCP: oral contraceptive pill use, NVQ: national vocational qualification, s/day: servings/day. | | | | |

| Additional file 1: Table 5. Baseline characteristics of participants by poultry meat intake in UK Biobank (n=473 011^a^). | | | |
| --- | --- | --- | --- |
| Characteristic | 0-1 times/week | 2 times/week | >3 times/week |
| Mean (SD) or n (%) | N=75 001 | N=169 471 | N=228 539 |
| **Sociodemographic** |  |  |  |
| Sex, n (%) |  |  |  |
| Women | 41 873 (55.8) | 88 922 (52.5) | 124 248 (54.4) |
| Men | 33 128 (44.2) | 80 549 (47.5) | 104 291 (45.6) |
| Age (years), mean (SD) | 56.6 (8.1) | 57.2 (7.9) | 55.5 (8.1) |
| Age (years), n (%) |  |  |  |
| 37-44 | 7 911 (10.5) | 14 808 (8.7) | 27 780 (12.2) |
| 45-49 | 9 375 (12.5) | 19 664 (11.6) | 34 935 (15.3) |
| 50-54 | 11 125 (14.8) | 24 136 (14.2) | 37 760 (16.5) |
| 55-59 | 13 868 (18.5) | 31 286 (18.5) | 40 704 (17.8) |
| 60-64 | 17 823 (23.8) | 44 002 (26.0) | 50 872 (22.3) |
| 65- 73 | 14 899 (19.9) | 35 575 (21.0) | 36 488 (16.0) |
| Race, n (%) |  |  |  |
| White | 69 345 (92.5) | 161 947 (95.6) | 214 119 (93.7) |
| Asian or Asian British | 3 216 (4.3) | 3 004 (1.8) | 4 822 (2.1) |
| Black or Black British | 903 (1.2) | 1 872 (1.1) | 4 937 (2.2) |
| Mixed race/Other | 1 161 (1.5) | 2 108 (1.2) | 3 913 (1.7) |
| Unknown | 376 (0.5) | 540 (0.3) | 748 (0.3) |
| Townsend deprivation, n (%) |  |  |  |
| Most affluent (mean -4.7) | 11 931 (15.9) | 35 279 (20.8) | 47 659 (20.9) |
| 2 (mean -3.3) | 13 030 (17.4) | 34 600 (20.4) | 46 633 (20.4) |
| 3 (mean -2.1) | 14 541 (19.4) | 34 074 (20.1) | 45 904 (20.1) |
| 4 (mean -0.1) | 16 874 (22.5) | 33 134 (19.6) | 44 577 (19.5) |
| Most deprived (mean 3.8) | 18 539 (24.7) | 32 214 (19.0) | 43 431 (19.0) |
| Unknown | 86 (0.1) | 170 (0.1) | 335 (0.1) |
| Qualification, n (%) |  |  |  |
| College/university degree/NVQ | 46 745 (62.3) | 98 309 (58.0) | 136 117 (59.6) |
| National examination at ages 17‑18 | 3 845 (5.1) | 8 752 (5.2) | 13 188 (5.8) |
| National examination at age 16 | 10 302 (13.7) | 27 991 (16.5) | 40 289 (17.6) |
| Other/unknown | 14 109 (18.8) | 34 419 (20.3) | 38 945 (17.0) |
| Employment, n (%) |  |  |  |
| In paid employment | 42 586 (56.8) | 93 617 (55.2) | 139 529 (61.1) |
| Pension | 21 966 (29.3) | 55 867 (33.0) | 62 015 (27.1) |
| Not in paid employment | 9 564 (12.8) | 18 407 (10.9) | 24 991 (10.9) |
| Unknown | 885 (1.2) | 1 580 (0.9) | 2 004 (0.9) |
| **Physical measurements** |  |  |  |
| BMI (kg/m^2^), mean (SD) | 26.6 (4.8) | 27.3 (4.6) | 27.8 (4.9) |
| BMI (kg/m^2^), fifths, n (%) |  |  |  |
| Q1 (mean W: 21 kg/m^2^, M: 23 kg/m^2^) | 20 962 (27.9) | 35 778 (21.1) | 40 818 (17.9) |
| Q2 (mean W: 24 kg/m^2^, M: 26 kg/m^2^) | 15 170 (20.2) | 34 053 (20.1) | 42 924 (18.8) |
| Q3 (mean W: 26 kg/m^2^, M: 27 kg/m^2^) | 14 193 (18.9) | 35 150 (20.7) | 46 822 (20.5) |
| Q4 (mean W: 29 kg/m^2^, M: 29 kg/m^2^) | 12 272 (16.4) | 32 634 (19.3) | 46 509 (20.4) |
| Q5 (mean W: 35 kg/m^2^, M: 34 kg/m^2^) | 11 943 (15.9) | 31 026 (18.3) | 50 458 (22.1) |
| Unknown | 461 (0.6) | 830 (0.5) | 1 008 (0.4) |
| **Lifestyle** |  |  |  |
| Smoking, n (%) |  |  |  |
| Never | 39 769 (53.0) | 91 570 (54.0) | 127 684 (55.9) |
| Former | 25 812 (34.4) | 58 456 (34.5) | 77 743 (34.0) |
| Current <15 cigarettes/day | 2 585 (3.4) | 5 268 (3.1) | 6 576 (2.9) |
| Current ≥15 cigarettes/day | 3 713 (5.0) | 7 630 (4.5) | 8 500 (3.7) |
| Current, amount unknown | 2 807 (3.7) | 5 880 (3.5) | 7 278 (3.2) |
| Unknown | 315 (0.4) | 667 (0.4) | 758 (0.3) |
| Physical activity level, n (%) |  |  |  |
| Low <10 excess METs | 23 397 (31.2) | 54 428 (32.1) | 71 413 (31.2) |
| Moderate 10-<50 excess METs | 36 614 (48.8) | 82 368 (48.6) | 111 391 (48.7) |
| High ≥ 50 excess METs | 12 013 (16.0) | 26 068 (15.4) | 37 327 (16.3) |
| Unknown | 2 977 (4.0) | 6 607 (3.9) | 8 408 (3.7) |
| Alcohol intake, n (%) |  |  |  |
| Non-drinkers | 8 852 (11.8) | 12 094 (7.1) | 16 938 (7.4) |
| <1g/d | 10 017 (13.4) | 18 201 (10.7) | 24 810 (10.9) |
| 1-<10g/d | 23 625 (31.5) | 53 598 (31.6) | 70 974 (31.1) |
| 10-<20g/d | 14 672 (19.6) | 37 461 (22.1) | 50 046 (21.9) |
| 20+g/d | 17 363 (23.2) | 47 266 (27.9) | 64 679 (28.3) |
| Unknown | 472 (0.6) | 851 (0.5) | 1 092 (0.5) |
| **Diet** |  |  |  |
| Fruit & vegetable intake (s/day), mean (SD) | 4.83 (2.97) | 4.50 (2.43) | 4.78 (2.60) |
| Fruit & vegetable intake categories, n (%) |  |  |  |
| <3 servings/day | 15 542 (20.7) | 35 581 (21.0) | 41 217 (18.0) |
| 3-<4 servings/day | 12 371 (16.5) | 32 865 (19.4) | 41 313 (18.1) |
| 4-<6 servings/day | 23 838 (31.8) | 58 974 (34.8) | 80 526 (35.2) |
| ≥6 servings/day | 21 158 (28.2) | 38 366 (22.6) | 61 577 (26.9) |
| Unknown | 2 092 (2.8) | 3 685 (2.2) | 3 906 (1.7) |
| Cereal fibre intake (g/day), mean (SD) | 4.64 (3.10) | 4.51 (2.91) | 4.46 (2.90) |
| Cereal/Bread fibre sex-spec Quintiles, n (%) |  |  |  |
| Q1 (mean W: 1.1 g/day, M: 1.3 g/day) | 14 679 (19.6) | 33 303 (19.7) | 45 002 (19.7) |
| Q2 (mean W: 2.5 g/day, M: 2.8 g/day) | 13 761 (18.3) | 33 731 (19.9) | 45 931 (20.1) |
| Q3 (mean W: 3.7 g/day, M: 4.3 g/day) | 14 013 (18.7) | 33 293 (19.6) | 45 550 (19.9) |
| Q4 (mean W: 5.3 g/day, M: 6.2 g/day) | 14 841 (19.8) | 32 634 (19.3) | 43 905 (19.2) |
| Q5 (mean W: 8.4 g/day, M: 9.9 g/day) | 15 912 (21.2) | 32 990 (19.5) | 43 469 (19.0) |
| Unknown | 1 795 (2.4) | 3 520 (2.1) | 4 682 (2.0) |
| Oily fish, n (%) |  |  |  |
| 0 times/week | 16 673 (22.2) | 14 255 (8.4) | 21 125 (9.2) |
| <1 times/week | 23 808 (31.7) | 58 093 (34.3) | 74 457 (32.6) |
| 1 time/week | 20 725 (27.6) | 69 167 (40.8) | 87 560 (38.3) |
| >2 times/week | 13 318 (17.8) | 26 970 (15.9) | 44 253 (19.4) |
| Unknown | 477 (0.6) | 986 (0.6) | 1 144 (0.5) |
| Non-oily fish, n (%) |  |  |  |
| <1 time/week | 36 374 (48.5) | 54 812 (32.3) | 68 327 (29.9) |
| 1 times/week | 27 120 (36.2) | 91 733 (54.1) | 115 161 (50.4) |
| >2 times/week | 11 035 (14.7) | 22 182 (13.1) | 44 042 (19.3) |
| Unknown | 472 (0.6) | 744 (0.4) | 1 009 (0.4) |
| Unprocessed red meat, n (%) |  |  |  |
| <1 times/week | 26 990 (36.0) | 8 114 (4.8) | 12 519 (5.5) |
| 1 times/week | 28 809 (38.4) | 67 608 (39.9) | 88 048 (38.5) |
| 2-3 times/week | 12 790 (17.1) | 72 906 (43.0) | 88 069 (38.5) |
| >4 times/week | 5 617 (7.5) | 18 818 (11.1) | 37 630 (16.5) |
| unknown/missing | 795 (1.1) | 2 025 (1.2) | 2 273 (1.0) |
| Processed meat, n (%) |  |  |  |
| 0 times/week | 24 778 (33.0) | 7 762 (4.6) | 11 430 (5.0) |
| <1 times/week | 21 748 (29.0) | 54 281 (32.0) | 67 327 (29.5) |
| 1 times/week | 12 657 (16.9) | 55 748 (32.9) | 69 459 (30.4) |
| >2 times/week | 15 650 (20.9) | 51 356 (30.3) | 79 960 (35.0) |
| Unknown | 168 (0.2) | 324 (0.2) | 363 (0.2) |
| **Women factors** |  |  |  |
| Menopausal status, n (%) |  |  |  |
| Premenopausal | 10 057 (24.0) | 18 215 (20.5) | 32 442 (26.1) |
| Postmenopausal | 29 711 (71.0) | 66 398 (74.7) | 84 027 (67.6) |
| Unknown | 2 105 (5.0) | 4 309 (4.8) | 7 779 (6.3) |
| Parity, n (%) |  |  |  |
| 0 births | 11 583 (27.7) | 16 043 (18.0) | 19 910 (16.0) |
| 1 - 2 births | 21 254 (50.8) | 51 258 (57.6) | 72 991 (58.7) |
| ≥3 births | 8 970 (21.4) | 21 529 (24.2) | 31 236 (25.1) |
| Unknown | 66 (0.2) | 92 (0.1) | 111 (0.1) |
| Hormone replacement therapy use (HRT), n (%) |  |  |  |
| Never | 27 195 (64.9) | 53 288 (59.9) | 77 466 (62.3) |
| Past | 12 070 (28.8) | 29 958 (33.7) | 38 493 (31.0) |
| Current | 2 400 (5.7) | 5 362 (6.0) | 7 835 (6.3) |
| Unknown | 208 (0.5) | 314 (0.4) | 454 (0.4) |
| Oral contraceptive pill use (OCT), n (%) |  |  |  |
| Never | 8 870 (21.2) | 17 361 (19.5) | 21 152 (17.0) |
| Past | 32 001 (76.4) | 69 795 (78.5) | 100 198 (80.6) |
| Current | 791 (1.9) | 1 535 (1.7) | 2 521 (2.0) |
| Unknown | 211 (0.5) | 231 (0.3) | 377 (0.3) |
| The x^2^ test was used to compare the distribution between meat intakes for all categorical variables. Analysis of variance (ANOVA) was used to compare the means between meat intakes. The *P*-heterogeneity between meat intakes was <0.001 for all variables. All dietary data come from the touchscreen questionnaire. **^a^**Additional file 1: Fig 1 BMI: Body mass index HRT: hormone replacement therapy, OCP: oral contraceptive pill use, NVQ: national vocational qualification, s/day: servings/day. | | | |

| Additional file 1: Table 6. Risk of 25 common conditions by total meat intake in UK Biobank. | | | | | | | | | | | | | | | | | | | | |
| --- | --- | --- | --- | --- | --- | --- | --- | --- | --- | --- | --- | --- | --- | --- | --- | --- | --- | --- | --- | --- |
|  | | | Total meat intake (times/week) | | | | | | | | | | | | |  | | | | |
| Disease | | | | Model | | | <3 | | | 3 - 4 | | 5 - 6 | | ≥7 | | | | per 100g/day increase | | p-value |
| Ischemic heart disease | | | | Mean grams/day | | | 33.43 (43.85) | | | 84.61 (47.04) | | 99.05 (48.56) | | 116.02 (53.01) | | | |  | |  |
|  | | | | **Total n/cases** | | | 54185/1239 | | | 110065/3158 | | 141542/4308 | | 124612/4401 | | | | 430404/13106 | |  |
|  | | | | HR (95%-CI), Model 0 | | | 1.00 (Ref) | | | 1.04 (0.97-1.11) | | 1.08 (1.01-1.15) | | 1.17 (1.10-1.25) | | | | 1.23 (1.14-1.33) | | <0.0001 |
|  | | | | HR (95%-CI), Model 1 | | | 1.00 (Ref) | | | 1.10 (1.03-1.18) | | 1.15 (1.08-1.23) | | 1.23 (1.15-1.31) | | | | 1.29 (1.20-1.40) | | <0.0001 |
|  | | | | HR (95%-CI), Model 2 | | | 1.00 (Ref) | | | 1.11 (1.04-1.19) | | 1.16 (1.09-1.24) | | 1.23 (1.15-1.32) | | | | 1.30 (1.20-1.40) | | <0.0001 |
|  | | | | HR (95%-CI), Model 3 | | | 1.00 (Ref) | | | 1.07 (1.00-1.14) | | 1.09 (1.02-1.17) | | 1.13 (1.06-1.21) | | | | 1.17 (1.08-1.26) | | 0.0001 |
|  | | | |  | | |  | | |  | |  | |  | | | |  | |  |
| Atrial Fibrillation and flutter | | | | Mean grams/day | | | 33.41 (43.84) | | | 84.64 (47.04) | | 99.07 (48.63) | | 116.03 (52.99) | | | |  | |  |
|  | | | | **Total n/cases** | | | 54185/495 | | | 110065/1179 | | 141542/1556 | | 124612/1507 | | | | 430404/4737 | |  |
|  | | | | HR (95%-CI), Model 0 | | | 1.00 (Ref) | | | 0.97 (0.87-1.08) | | 1.00 (0.91-1.11) | | 1.07 (0.97-1.19) | | | | 1.11 (0.98-1.25) | | 0.1104 |
|  | | | | HR (95%-CI), Model 1 | | | 1.00 (Ref) | | | 0.95 (0.85-1.05) | | 0.97 (0.87-1.07) | | 1.03 (0.92-1.14) | | | | 1.04 (0.92-1.18) | | 0.4941 |
|  | | | | HR (95%-CI), Model 2 | | | 1.00 (Ref) | | | 0.97 (0.87-1.08) | | 0.99 (0.89-1.10) | | 1.05 (0.94-1.16) | | | | 1.07 (0.94-1.21) | | 0.2993 |
|  | | | | HR (95%-CI), Model 3 | | | 1.00 (Ref) | | | 0.93 (0.83-1.03) | | 0.93 (0.83-1.03) | | 0.95 (0.86-1.06) | | | | 0.95 (0.84-1.08) | | 0.4166 |
|  | | | |  | | |  | | |  | |  | |  | | | |  | |  |
| Cerebrovascular | | | | Mean grams/day | | | 33.44 (43.85) | | | 84.65 (47.03) | | 99.11 (48.61) | | 115.99 (52.99) | | | |  | |  |
|  | | | | **Total n/cases** | | | 54185/534 | | | 110065/1328 | | 141542/1806 | | 124612/1656 | | | | 430404/5324 | |  |
|  | | | | HR (95%-CI), Model 0 | | | 1.00 (Ref) | | | 1.04 (0.94-1.15) | | 1.12 (1.02-1.23) | | 1.17 (1.06-1.29) | | | | 1.24 (1.10-1.39) | | 0.0004 |
|  | | | | HR (95%-CI), Model 1 | | | 1.00 (Ref) | | | 1.05 (0.95-1.17) | | 1.14 (1.03-1.25) | | 1.15 (1.04-1.28) | | | | 1.21 (1.08-1.37) | | 0.0014 |
|  | | | | HR (95%-CI), Model 2 | | | 1.00 (Ref) | | | 1.07 (0.97-1.19) | | 1.16 (1.05-1.28) | | 1.17 (1.05-1.29) | | | | 1.23 (1.09-1.38) | | 0.0008 |
|  | | | | HR (95%-CI), Model 3 | | | 1.00 (Ref) | | | 1.06 (0.95-1.17) | | 1.13 (1.02-1.25) | | 1.13 (1.02-1.25) | | | | 1.18 (1.04-1.33) | | 0.0083 |
|  | | | |  | | |  | | |  | |  | |  | | | |  | |  |
| Ischaemic stroke | | | | Mean grams/day | | | 33.43 (43.84) | | | 84.64 (47.03) | | 99.09 (48.60) | | 116.00 (52.98) | | | |  | |  |
|  | | | | **Total n/cases** | | | 54185/222 | | | 110065/570 | | 141542/787 | | 124612/757 | | | | 430404/2336 | |  |
|  | | | | HR (95%-CI), Model 0 | | | 1.00 (Ref) | | | 1.03 (0.88-1.20) | | 1.12 (0.96-1.30) | | 1.21 (1.04-1.41) | | | | 1.30 (1.08-1.56) | | 0.0048 |
|  | | | | HR (95%-CI), Model 1 | | | 1.00 (Ref) | | | 1.06 (0.91-1.24) | | 1.16 (1.00-1.35) | | 1.21 (1.04-1.41) | | | | 1.29 (1.07-1.55) | | 0.0062 |
|  | | | | HR (95%-CI), Model 2 | | | 1.00 (Ref) | | | 1.10 (0.94-1.29) | | 1.20 (1.03-1.40) | | 1.24 (1.06-1.45) | | | | 1.33 (1.10-1.59) | | 0.0025 |
|  | | | | HR (95%-CI), Model 3 | | | 1.00 (Ref) | | | 1.07 (0.91-1.25) | | 1.15 (0.98-1.34) | | 1.16 (1.00-1.36) | | | | 1.22 (1.02-1.47) | | 0.0321 |
|  | | | |  | | |  | | |  | |  | |  | | | |  | |  |
| Haemorrhagic stroke | | | | Mean grams/day | | | 33.43 (43.84) | | | 84.62 (47.02) | | 99.08 (48.61) | | 116.00 (52.99) | | | |  | |  |
|  | | | | **Total n/cases** | | | 54185/113 | | | 110065/237 | | 141542/322 | | 124612/267 | | | | 430404/939 | |  |
|  | | | | HR (95%-CI), Model 0 | | | 1.00 (Ref) | | | 0.94 (0.75-1.18) | | 1.04 (0.84-1.29) | | 1.02 (0.82-1.28) | | | | 1.06 (0.81-1.38) | | 0.6827 |
|  | | | | HR (95%-CI), Model 1 | | | 1.00 (Ref) | | | 0.96 (0.76-1.20) | | 1.06 (0.85-1.32) | | 1.01 (0.81-1.27) | | | | 1.05 (0.80-1.38) | | 0.7087 |
|  | | | | HR (95%-CI), Model 2 | | | 1.00 (Ref) | | | 0.97 (0.77-1.21) | | 1.06 (0.85-1.33) | | 1.01 (0.81-1.27) | | | | 1.05 (0.80-1.38) | | 0.7211 |
|  | | | | HR (95%-CI), Model 3 | | | 1.00 (Ref) | | | 0.98 (0.78-1.23) | | 1.09 (0.87-1.36) | | 1.04 (0.83-1.31) | | | | 1.09 (0.83-1.43) | | 0.5472 |
|  | | | |  | | |  | | |  | |  | |  | | | |  | |  |
| Venous thromboembolism | | | | Mean grams/day | | | 33.81 (44.02) | | | 84.73 (47.00) | | 99.15 (48.71) | | 116.07 (52.98) | | | |  | |  |
|  | | | | **Total n/cases** | | | 56786/350 | | | 116628/966 | | 150259/1284 | | 133382/1147 | | | | 457055/3747 | |  |
|  | | | | HR (95%-CI), Model 0 | | | 1.00 (Ref) | | | 1.18 (1.05-1.34) | | 1.22 (1.09-1.38) | | 1.22 (1.08-1.38) | | | | 1.27 (1.10-1.46) | | 0.0009 |
|  | | | | HR (95%-CI), Model 1 | | | 1.00 (Ref) | | | 1.19 (1.05-1.35) | | 1.24 (1.10-1.40) | | 1.23 (1.09-1.39) | | | | 1.28 (1.11-1.47) | | 0.0008 |
|  | | | | HR (95%-CI), Model 2 | | | 1.00 (Ref) | | | 1.21 (1.07-1.37) | | 1.26 (1.11-1.42) | | 1.24 (1.10-1.41) | | | | 1.29 (1.12-1.49) | | 0.0005 |
|  | | | | HR (95%-CI), Model 3 | | | 1.00 (Ref) | | | 1.14 (1.01-1.29) | | 1.15 (1.02-1.30) | | 1.10 (0.97-1.25) | | | | 1.11 (0.96-1.28) | | 0.1478 |
|  | | | |  | | |  | | |  | |  | |  | | | |  | |  |
| Varicose veins | | | | Mean grams/day | | | 33.62 (43.86) | | | 84.95 (47.03) | | 99.37 (48.75) | | 116.22 (53.12) | | | |  | |  |
|  | | | | **Total n/cases** | | | 55246/325 | | | 112957/679 | | 146292/864 | | 130670/765 | | | | 445165/2633 | |  |
|  | | | | HR (95%-CI), Model 0 | | | 1.00 (Ref) | | | 1.02 (0.89-1.16) | | 1.01 (0.88-1.14) | | 1.03 (0.90-1.18) | | | | 1.03 (0.88-1.20) | | 0.7037 |
|  | | | | HR (95%-CI), Model 1 | | | 1.00 (Ref) | | | 1.01 (0.88-1.15) | | 0.99 (0.87-1.13) | | 1.01 (0.89-1.16) | | | | 1.01 (0.86-1.18) | | 0.9173 |
|  | | | | HR (95%-CI), Model 2 | | | 1.00 (Ref) | | | 1.02 (0.89-1.17) | | 1.01 (0.88-1.15) | | 1.03 (0.90-1.18) | | | | 1.03 (0.88-1.21) | | 0.6954 |
|  | | | | HR (95%-CI), Model 3 | | | 1.00 (Ref) | | | 1.00 (0.87-1.15) | | 0.97 (0.85-1.11) | | 0.98 (0.86-1.13) | | | | 0.97 (0.83-1.14) | | 0.7313 |
|  | | | |  | | |  | | |  | |  | |  | | | |  | |  |
| Haemorrhoids | | | | Mean grams/day | | | 33.68 (43.93) | | | 84.62 (46.89) | | 99.19 (48.74) | | 116.14 (53.10) | | | |  | |  |
|  | | | | **Total n/cases** | | | 55675/995 | | | 114393/2012 | | 147269/2762 | | 130445/2518 | | | | 447782/8287 | |  |
|  | | | | HR (95%-CI), Model 0 | | | 1.00 (Ref) | | | 0.97 (0.90-1.04) | | 1.02 (0.95-1.10) | | 1.03 (0.96-1.11) | | | | 1.06 (0.97-1.15) | | 0.2307 |
|  | | | | HR (95%-CI), Model 1 | | | 1.00 (Ref) | | | 0.98 (0.91-1.06) | | 1.03 (0.96-1.11) | | 1.05 (0.97-1.13) | | | | 1.07 (0.98-1.17) | | 0.1308 |
|  | | | | HR (95%-CI), Model 2 | | | 1.00 (Ref) | | | 0.99 (0.91-1.07) | | 1.04 (0.97-1.12) | | 1.05 (0.98-1.14) | | | | 1.08 (0.99-1.19) | | 0.0871 |
|  | | | | HR (95%-CI), Model 3 | | | 1.00 (Ref) | | | 0.99 (0.91-1.07) | | 1.04 (0.97-1.13) | | 1.06 (0.98-1.14) | | | | 1.09 (0.99-1.19) | | 0.0800 |
|  | | | |  | | |  | | |  | |  | |  | | | |  | |  |
| Pneumonia | | | | Mean grams/day | | | 33.71 (44.00) | | | 84.81 (46.92) | | 99.22 (48.63) | | 116.02 (53.01) | | | |  | |  |
|  | | | | **Total n/cases** | | | 56982/669 | | | 117178/1539 | | 150902/2030 | | 133895/2096 | | | | 458957/6334 | |  |
|  | | | | HR (95%-CI), Model 0 | | | 1.00 (Ref) | | | 0.97 (0.89-1.06) | | 1.01 (0.93-1.11) | | 1.19 (1.09-1.30) | | | | 1.26 (1.13-1.41) | | <0.0001 |
|  | | | | HR (95%-CI), Model 1 | | | 1.00 (Ref) | | | 1.02 (0.93-1.11) | | 1.07 (0.98-1.17) | | 1.20 (1.09-1.31) | | | | 1.27 (1.14-1.41) | | <0.0001 |
|  | | | | HR (95%-CI), Model 2 | | | 1.00 (Ref) | | | 1.04 (0.94-1.14) | | 1.09 (1.00-1.19) | | 1.21 (1.11-1.33) | | | | 1.29 (1.15-1.43) | | <0.0001 |
|  | | | | HR (95%-CI), Model 3 | | | 1.00 (Ref) | | | 1.03 (0.94-1.13) | | 1.08 (0.98-1.18) | | 1.19 (1.08-1.30) | | | | 1.25 (1.12-1.39) | | 0.0001 |
|  | | | |  | | |  | | |  | |  | |  | | | |  | |  |
| Gastro-oesophageal reflux disease | | | | Mean grams/day | | | 33.82 (44.15) | | | 84.64 (46.92) | | 99.13 (48.66) | | 116.13 (52.99) | | | |  | |  |
|  | | | | **Total n/cases** | | | 55029/841 | | | 112249/1776 | | 144261/2328 | | 127988/2058 | | | | 439527/7003 | |  |
|  | | | | HR (95%-CI), Model 0 | | | 1.00 (Ref) | | | 0.99 (0.92-1.08) | | 1.03 (0.95-1.12) | | 1.07 (0.98-1.16) | | | | 1.09 (0.99-1.20) | | 0.0886 |
|  | | | | HR (95%-CI), Model 1 | | | 1.00 (Ref) | | | 1.00 (0.92-1.09) | | 1.05 (0.97-1.13) | | 1.08 (0.99-1.17) | | | | 1.10 (1.00-1.22) | | 0.0503 |
|  | | | | HR (95%-CI), Model 2 | | | 1.00 (Ref) | | | 1.03 (0.94-1.12) | | 1.07 (0.99-1.16) | | 1.10 (1.01-1.19) | | | | 1.13 (1.02-1.25) | | 0.0182 |
|  | | | | HR (95%-CI), Model 3 | | | 1.00 (Ref) | | | 1.01 (0.93-1.10) | | 1.04 (0.96-1.13) | | 1.06 (0.98-1.16) | | | | 1.08 (0.98-1.20) | | 0.1134 |
|  | | | |  | | |  | | |  | |  | |  | | | |  | |  |
| Gastritis and duodenitis | | | | Mean grams/day | | | 33.66 (43.94) | | | 84.73 (47.03) | | 99.25 (48.73) | | 116.05 (53.05) | | | |  | |  |
|  | | | | **Total n/cases** | | | 55990/1431 | | | 115129/2934 | | 148158/3626 | | 131386/3354 | | | | 450663/11345 | |  |
|  | | | | HR (95%-CI), Model 0 | | | 1.00 (Ref) | | | 0.97 (0.91-1.03) | | 0.95 (0.89-1.01) | | 1.03 (0.97-1.10) | | | | 1.02 (0.95-1.10) | | 0.5859 |
|  | | | | HR (95%-CI), Model 1 | | | 1.00 (Ref) | | | 1.01 (0.94-1.07) | | 0.99 (0.93-1.06) | | 1.06 (0.99-1.13) | | | | 1.06 (0.98-1.14) | | 0.1450 |
|  | | | | HR (95%-CI), Model 2 | | | 1.00 (Ref) | | | 1.02 (0.96-1.09) | | 1.01 (0.95-1.08) | | 1.07 (1.00-1.14) | | | | 1.07 (0.99-1.16) | | 0.0684 |
|  | | | | HR (95%-CI), Model 3 | | | 1.00 (Ref) | | | 1.02 (0.95-1.09) | | 1.00 (0.94-1.07) | | 1.06 (0.99-1.13) | | | | 1.06 (0.98-1.14) | | 0.1613 |
|  | | | |  | | |  | | |  | |  | |  | | | |  | |  |
| Inguinal hernia | | | | Mean grams/day | | | 33.66 (44.00) | | | 84.63 (46.84) | | 99.06 (48.57) | | 116.26 (53.10) | | | |  | |  |
|  | | | | **Total n/cases** | | | 55414/765 | | | 112796/1918 | | 144844/2655 | | 127373/2677 | | | | 440427/8015 | |  |
|  | | | | HR (95%-CI), Model 0 | | | 1.00 (Ref) | | | 0.94 (0.86-1.02) | | 0.92 (0.85-1.00) | | 0.87 (0.81-0.95) | | | | 0.85 (0.77-0.93) | | 0.0006 |
|  | | | | HR (95%-CI), Model 1 | | | 1.00 (Ref) | | | 0.93 (0.85-1.01) | | 0.91 (0.84-0.99) | | 0.88 (0.81-0.95) | | | | 0.85 (0.77-0.94) | | 0.0011 |
|  | | | | HR (95%-CI), Model 2 | | | 1.00 (Ref) | | | 0.93 (0.86-1.02) | | 0.92 (0.84-1.00) | | 0.89 (0.82-0.96) | | | | 0.86 (0.78-0.95) | | 0.0031 |
|  | | | | HR (95%-CI), Model 3 | | | 1.00 (Ref) | | | 0.99 (0.91-1.08) | | 1.00 (0.92-1.09) | | 1.00 (0.92-1.09) | | | | 1.00 (0.91-1.11) | | 0.9387 |
|  | | | |  | | |  | | |  | |  | |  | | | |  | |  |
| Noninfective enteritis and colitis | | | | Mean grams/day | | | 33.64 (43.94) | | | 84.81 (47.01) | | 99.14 (48.64) | | 116.25 (53.07) | | | |  | |  |
|  | | | | **Total n/cases** | | | 56242/804 | | | 115728/1568 | | 148973/2008 | | 132337/1851 | | | | 453280/6231 | |  |
|  | | | | HR (95%-CI), Model 0 | | | 1.00 (Ref) | | | 0.92 (0.85-1.01) | | 0.94 (0.87-1.02) | | 1.03 (0.95-1.12) | | | | 1.04 (0.94-1.15) | | 0.4799 |
|  | | | | HR (95%-CI), Model 1 | | | 1.00 (Ref) | | | 0.94 (0.86-1.02) | | 0.96 (0.88-1.05) | | 1.04 (0.95-1.13) | | | | 1.04 (0.94-1.16) | | 0.4095 |
|  | | | | HR (95%-CI), Model 2 | | | 1.00 (Ref) | | | 0.96 (0.88-1.05) | | 0.98 (0.90-1.07) | | 1.05 (0.96-1.15) | | | | 1.06 (0.96-1.18) | | 0.2430 |
|  | | | | HR (95%-CI), Model 3 | | | 1.00 (Ref) | | | 0.95 (0.87-1.04) | | 0.97 (0.89-1.06) | | 1.03 (0.95-1.13) | | | | 1.04 (0.94-1.15) | | 0.4690 |
|  | | | |  | | |  | | |  | |  | |  | | | |  | |  |
| Diverticular disease | | | | Mean grams/day | | | 33.60 (43.85) | | | 84.67 (47.05) | | 99.12 (48.75) | | 116.25 (53.03) | | | |  | |  |
|  | | | | **Total n/cases** | | | 56724/1312 | | | 116036/3286 | | 149455/4312 | | 132720/3902 | | | | 454935/12812 | |  |
|  | | | | HR (95%-CI), Model 0 | | | 1.00 (Ref) | | | 1.14 (1.07-1.21) | | 1.20 (1.12-1.27) | | 1.28 (1.20-1.37) | | | | 1.36 (1.26-1.46) | | <0.0001 |
|  | | | | HR (95%-CI), Model 1 | | | 1.00 (Ref) | | | 1.11 (1.04-1.18) | | 1.16 (1.09-1.23) | | 1.23 (1.15-1.31) | | | | 1.29 (1.20-1.40) | | <0.0001 |
|  | | | | HR (95%-CI), Model 2 | | | 1.00 (Ref) | | | 1.11 (1.04-1.19) | | 1.16 (1.09-1.24) | | 1.23 (1.15-1.31) | | | | 1.29 (1.20-1.39) | | <0.0001 |
|  | | | | HR (95%-CI), Model 3 | | | 1.00 (Ref) | | | 1.07 (1.01-1.15) | | 1.10 (1.03-1.18) | | 1.15 (1.08-1.22) | | | | 1.18 (1.10-1.28) | | <0.0001 |
|  | | | |  | | |  | | |  | |  | |  | | | |  | |  |
| Colon polyps | | | | Mean grams/day | | | 33.50 (43.92) | | | 84.71 (47.11) | | 99.26 (48.73) | | 116.27 (53.05) | | | |  | |  |
|  | | | | **Total n/cases** | | | 55459/4242 | | | 113821/9487 | | 146551/12226 | | 129887/11412 | | | | 445718/37367 | |  |
|  | | | | HR (95%-CI), Model 0 | | | 1.00 (Ref) | | | 1.03 (0.99-1.06) | | 1.04 (1.01-1.08) | | 1.14 (1.10-1.18) | | | | 1.17 (1.12-1.22) | | <0.0001 |
|  | | | | HR (95%-CI), Model 1 | | | 1.00 (Ref) | | | 1.02 (0.99-1.06) | | 1.04 (1.00-1.08) | | 1.12 (1.08-1.16) | | | | 1.14 (1.09-1.19) | | <0.0001 |
|  | | | | HR (95%-CI), Model 2 | | | 1.00 (Ref) | | | 1.03 (0.99-1.07) | | 1.05 (1.01-1.09) | | 1.12 (1.08-1.16) | | | | 1.15 (1.10-1.20) | | <0.0001 |
|  | | | | HR (95%-CI), Model 3 | | | 1.00 (Ref) | | | 1.01 (0.98-1.05) | | 1.02 (0.98-1.06) | | 1.08 (1.04-1.12) | | | | 1.10 (1.05-1.15) | | <0.0001 |
|  | | | |  | | |  | | |  | |  | |  | | | |  | |  |
| Gallbladder disease | | | | Mean grams/day | | | 33.49 (43.76) | | | 84.68 (47.10) | | 99.16 (48.73) | | 116.16 (53.03) | | | |  | |  |
|  | | | | **Total n/cases** | | | 55802/1002 | | | 114055/2239 | | 146959/3021 | | 131228/2645 | | | | 448044/8907 | |  |
|  | | | | HR (95%-CI), Model 0 | | | 1.00 (Ref) | | | 1.09 (1.01-1.18) | | 1.19 (1.11-1.28) | | 1.28 (1.19-1.38) | | | | 1.37 (1.25-1.49) | | <0.0001 |
|  | | | | HR (95%-CI), Model 1 | | | 1.00 (Ref) | | | 1.10 (1.02-1.19) | | 1.21 (1.12-1.30) | | 1.30 (1.21-1.41) | | | | 1.40 (1.28-1.53) | | <0.0001 |
|  | | | | HR (95%-CI), Model 2 | | | 1.00 (Ref) | | | 1.13 (1.05-1.22) | | 1.24 (1.15-1.33) | | 1.32 (1.23-1.43) | | | | 1.43 (1.31-1.56) | | <0.0001 |
|  | | | | HR (95%-CI), Model 3 | | | 1.00 (Ref) | | | 1.03 (0.95-1.11) | | 1.07 (0.99-1.15) | | 1.09 (1.01-1.18) | | | | 1.12 (1.03-1.23) | | 0.0117 |
|  | | | |  | | |  | | |  | |  | |  | | | |  | |  |
| Osteoarthritis | | | | Mean grams/day | | | 33.19 (43.73) | | | 84.65 (47.06) | | 99.12 (48.78) | | 116.06 (52.99) | | | |  | |  |
|  | | | | **Total n/cases** | | | 52411/1950 | | | 106121/4783 | | 136830/5994 | | 121524/5336 | | | | 416886/18063 | |  |
|  | | | | HR (95%-CI), Model 0 | | | 1.00 (Ref) | | | 1.10 (1.04-1.16) | | 1.12 (1.07-1.18) | | 1.22 (1.15-1.28) | | | | 1.26 (1.18-1.34) | | <0.0001 |
|  | | | | HR (95%-CI), Model 1 | | | 1.00 (Ref) | | | 1.08 (1.03-1.14) | | 1.10 (1.04-1.16) | | 1.18 (1.12-1.24) | | | | 1.21 (1.14-1.29) | | <0.0001 |
|  | | | | HR (95%-CI), Model 2 | | | 1.00 (Ref) | | | 1.10 (1.05-1.16) | | 1.12 (1.06-1.18) | | 1.20 (1.14-1.27) | | | | 1.24 (1.16-1.32) | | <0.0001 |
|  | | | | HR (95%-CI), Model 3 | | | 1.00 (Ref) | | | 1.02 (0.97-1.08) | | 0.99 (0.94-1.05) | | 1.02 (0.97-1.08) | | | | 1.02 (0.95-1.08) | | 0.6017 |
|  | | | |  | | |  | | |  | |  | |  | | | |  | |  |
| Kidney stones | | | | Mean grams/day | | | 33.66 (43.89) | | | 84.75 (46.97) | | 99.10 (48.67) | | 115.97 (52.92) | | | |  | |  |
|  | | | | **Total n/cases** | | | 57245/258 | | | 117599/611 | | 151279/758 | | 134086/812 | | | | 460209/2439 | |  |
|  | | | | HR (95%-CI), Model 0 | | | 1.00 (Ref) | | | 1.08 (0.93-1.25) | | 0.99 (0.86-1.14) | | 1.09 (0.94-1.25) | | | | 1.07 (0.91-1.27) | | 0.4110 |
|  | | | | HR (95%-CI), Model 1 | | | 1.00 (Ref) | | | 1.13 (0.98-1.31) | | 1.07 (0.92-1.23) | | 1.18 (1.02-1.36) | | | | 1.19 (1.00-1.40) | | 0.0502 |
|  | | | | HR (95%-CI), Model 2 | | | 1.00 (Ref) | | | 1.13 (0.97-1.31) | | 1.06 (0.91-1.22) | | 1.15 (1.00-1.33) | | | | 1.16 (0.97-1.37) | | 0.1000 |
|  | | | | HR (95%-CI), Model 3 | | | 1.00 (Ref) | | | 1.07 (0.92-1.25) | | 0.98 (0.85-1.14) | | 1.05 (0.91-1.21) | | | | 1.03 (0.86-1.22) | | 0.7550 |
|  | | | |  | | |  | | |  | |  | |  | | | |  | |  |
| Urinary tract infection | | | | Mean grams/day | | | 33.86 (43.98) | | | 84.81 (46.99) | | 99.17 (48.75) | | 116.13 (53.03) | | | |  | |  |
|  | | | | **Total n/cases** | | | 56857/609 | | | 117287/1325 | | 151151/1701 | | 134345/1560 | | | | 459640/5195 | |  |
|  | | | | HR (95%-CI), Model 0 | | | 1.00 (Ref) | | | 0.94 (0.86-1.04) | | 0.96 (0.88-1.06) | | 1.03 (0.94-1.13) | | | | 1.04 (0.93-1.17) | | 0.5049 |
|  | | | | HR (95%-CI), Model 1 | | | 1.00 (Ref) | | | 0.99 (0.89-1.09) | | 1.02 (0.93-1.12) | | 1.07 (0.97-1.18) | | | | 1.09 (0.97-1.22) | | 0.1408 |
|  | | | | HR (95%-CI), Model 2 | | | 1.00 (Ref) | | | 1.01 (0.91-1.11) | | 1.03 (0.94-1.14) | | 1.08 (0.98-1.19) | | | | 1.10 (0.98-1.24) | | 0.0999 |
|  | | | | HR (95%-CI), Model 3 | | | 1.00 (Ref) | | | 0.98 (0.88-1.08) | | 0.99 (0.90-1.09) | | 1.01 (0.91-1.11) | | | | 1.02 (0.90-1.14) | | 0.8009 |
|  | | | |  | | |  | | |  | |  | |  | | | |  | |  |
| Enlarged prostate | | | | Mean grams/day | | | 35.40 (46.61) | | | 89.41 (49.98) | | 105.29 (51.90) | | 122.25 (55.44) | | | |  | |  |
|  | | | | **Total n/cases** | | | 17550/308 | | | 44943/813 | | 66541/1192 | | 76369/1222 | | | | 205403/3535 | |  |
|  | | | | HR (95%-CI), Model 0 | | | 1.00 (Ref) | | | 0.91 (0.80-1.04) | | 0.94 (0.83-1.07) | | 0.90 (0.79-1.02) | | | | 0.89 (0.77-1.03) | | 0.1318 |
|  | | | | HR (95%-CI), Model 1 | | | 1.00 (Ref) | | | 0.96 (0.84-1.10) | | 1.02 (0.90-1.16) | | 0.98 (0.86-1.12) | | | | 1.00 (0.86-1.16) | | 0.9988 |
|  | | | | HR (95%-CI), Model 2 | | | 1.00 (Ref) | | | 0.97 (0.85-1.11) | | 1.03 (0.91-1.18) | | 1.00 (0.88-1.13) | | | | 1.02 (0.87-1.18) | | 0.8350 |
|  | | | | HR (95%-CI), Model 3 | | | 1.00 (Ref) | | | 0.97 (0.85-1.11) | | 1.04 (0.91-1.18) | | 1.00 (0.88-1.14) | | | | 1.02 (0.88-1.19) | | 0.7629 |
|  | | | |  | | |  | | |  | |  | |  | | | |  | |  |
| Female genital prolapse | | | | Mean grams/day | | | 32.94 (42.82) | | | 81.75 (44.84) | | 94.47 (45.67) | | 108.47 (48.71) | | | |  | |  |
|  | | | | **Total n/cases** | | | 37988/816 | | | 68348/1688 | | 79381/2090 | | 53796/1464 | | | | 239513/6058 | |  |
|  | | | | HR (95%-CI), Model 0 | | | 1.00 (Ref) | | | 1.07 (0.99-1.17) | | 1.17 (1.07-1.26) | | 1.24 (1.14-1.35) | | | | 1.30 (1.18-1.44) | | <0.0001 |
|  | | | | HR (95%-CI), Model 1 | | | 1.00 (Ref) | | | 0.99 (0.91-1.07) | | 1.04 (0.96-1.13) | | 1.10 (1.01-1.20) | | | | 1.12 (1.01-1.24) | | 0.0277 |
|  | | | | HR (95%-CI), Model 2 | | | 1.00 (Ref) | | | 1.01 (0.93-1.10) | | 1.07 (0.98-1.16) | | 1.13 (1.04-1.24) | | | | 1.16 (1.05-1.29) | | 0.0046 |
|  | | | | HR (95%-CI), Model 3 | | | 1.00 (Ref) | | | 0.98 (0.90-1.06) | | 1.01 (0.93-1.10) | | 1.06 (0.97-1.16) | | | | 1.08 (0.97-1.20) | | 0.1608 |
|  | | | |  | | |  | | |  | |  | |  | | | |  | |  |
| Uterine fibroids | | | | Mean grams/day | | | 31.60 (41.73) | | | 81.20 (45.18) | | 93.90 (45.43) | | 108.59 (48.64) | | | |  | |  |
|  | | | | **Total n/cases** | | | 29637/1485 | | | 51538/2464 | | 59888/3103 | | 40612/2198 | | | | 181675/9250 | |  |
|  | | | | HR (95%-CI), Model 0 | | | 1.00 (Ref) | | | 1.00 (0.94-1.07) | | 1.06 (0.99-1.12) | | 1.08 (1.01-1.15) | | | | 1.10 (1.02-1.18) | | 0.0173 |
|  | | | | HR (95%-CI), Model 1 | | | 1.00 (Ref) | | | 1.01 (0.95-1.08) | | 1.08 (1.01-1.15) | | 1.08 (1.01-1.15) | | | | 1.11 (1.02-1.20) | | 0.0099 |
|  | | | | HR (95%-CI), Model 2 | | | 1.00 (Ref) | | | 1.02 (0.95-1.09) | | 1.08 (1.02-1.15) | | 1.08 (1.01-1.16) | | | | 1.12 (1.03-1.21) | | 0.0064 |
|  | | | | HR (95%-CI), Model 3 | | | 1.00 (Ref) | | | 0.98 (0.92-1.05) | | 1.01 (0.95-1.08) | | 0.99 (0.92-1.06) | | | | 1.00 (0.92-1.08) | | 0.9277 |
|  | | | |  | | |  | | |  | |  | |  | | | |  | |  |
| Iron deficiency anaemia | | | | Mean grams/day | | | 33.89 (44.00) | | | 84.81 (46.96) | | 99.22 (48.74) | | 116.25 (53.07) | | | |  | |  |
|  | | | | **Total n/cases** | | | 56879/709 | | | 117843/1256 | | 151931/1483 | | 135100/1260 | | | | 461753/4708 | |  |
|  | | | | HR (95%-CI), Model 0 | | | 1.00 (Ref) | | | 0.80 (0.73-0.88) | | 0.76 (0.69-0.83) | | 0.76 (0.70-0.84) | | | | 0.71 (0.63-0.79) | | <0.0001 |
|  | | | | HR (95%-CI), Model 1 | | | 1.00 (Ref) | | | 0.90 (0.82-0.98) | | 0.87 (0.79-0.95) | | 0.87 (0.79-0.95) | | | | 0.84 (0.75-0.93) | | 0.0017 |
|  | | | | HR (95%-CI), Model 2 | | | 1.00 (Ref) | | | 0.94 (0.85-1.03) | | 0.90 (0.82-0.99) | | 0.90 (0.82-0.99) | | | | 0.87 (0.78-0.98) | | 0.0184 |
|  | | | | HR (95%-CI), Model 3 | | | 1.00 (Ref) | | | 0.92 (0.84-1.01) | | 0.87 (0.80-0.96) | | 0.86 (0.78-0.94) | | | | 0.82 (0.73-0.92) | | 0.0008 |
|  | | | |  | | |  | | |  | |  | |  | | | |  | |  |
| Diabetes | | | | Mean grams/day | | | 33.60 (43.85) | | | 84.52 (47.00) | | 98.90 (48.57) | | 115.65 (52.56) | | | |  | |  |
|  | | | | **Total n/cases** | | | 55408/911 | | | 113640/2161 | | 145662/3122 | | 127719/3362 | | | | 442429/9556 | |  |
|  | | | | HR (95%-CI), Model 0 | | | 1.00 (Ref) | | | 1.03 (0.95-1.11) | | 1.16 (1.08-1.25) | | 1.41 (1.31-1.52) | | | | 1.62 (1.47-1.77) | | <0.0001 |
|  | | | | HR (95%-CI), Model 1 | | | 1.00 (Ref) | | | 1.18 (1.09-1.27) | | 1.37 (1.27-1.48) | | 1.59 (1.48-1.72) | | | | 1.85 (1.69-2.03) | | <0.0001 |
|  | | | | HR (95%-CI), Model 2 | | | 1.00 (Ref) | | | 1.20 (1.11-1.30) | | 1.39 (1.29-1.50) | | 1.61 (1.49-1.73) | | | | 1.86 (1.70-2.04) | | <0.0001 |
|  | | | | HR (95%-CI), Model 3 | | | 1.00 (Ref) | | | 1.06 (0.98-1.15) | | 1.14 (1.06-1.23) | | 1.23 (1.14-1.33) | | | | 1.33 (1.21-1.46) | | <0.0001 |
|  | | | |  | | |  | | |  | |  | |  | | | |  | |  |
| Carpal tunnel syndrome | | | | Mean grams/day | | | 33.62 (43.78) | | | 84.69 (47.01) | | 99.21 (48.72) | | 116.09 (52.95) | | | |  | |  |
|  | | | | **Total n/cases** | | | 56857/558 | | | 116801/1308 | | 150601/1730 | | 133876/1448 | | | | 458135/5044 | |  |
|  | | | | HR (95%-CI), Model 0 | | | 1.00 (Ref) | | | 1.14 (1.03-1.26) | | 1.21 (1.10-1.33) | | 1.24 (1.13-1.37) | | | | 1.31 (1.17-1.48) | | <0.0001 |
|  | | | | HR (95%-CI), Model 1 | | | 1.00 (Ref) | | | 1.16 (1.05-1.28) | | 1.23 (1.12-1.36) | | 1.24 (1.12-1.37) | | | | 1.31 (1.16-1.47) | | <0.0001 |
|  | | | | HR (95%-CI), Model 2 | | | 1.00 (Ref) | | | 1.18 (1.07-1.31) | | 1.26 (1.14-1.39) | | 1.26 (1.14-1.40) | | | | 1.34 (1.19-1.50) | | <0.0001 |
|  | | | | HR (95%-CI), Model 3 | | | 1.00 (Ref) | | | 1.10 (0.99-1.22) | | 1.12 (1.02-1.24) | | 1.08 (0.98-1.20) | | | | 1.10 (0.98-1.24) | | 0.1015 |
|  | | | |  | | |  | | |  | |  | |  | | | |  | |  |
| Cataracts | | | | Mean grams/day | | | 33.53 (43.89) | | | 84.52 (47.08) | | 98.99 (48.61) | | 115.87 (52.60) | | | |  | |  |
|  | | | | **Total n/cases** | | | 53185/2028 | | | 109124/4466 | | 140142/5259 | | 123232/4345 | | | | 425683/16098 | |  |
|  | | | | HR (95%-CI), Model 0 | | | 1.00 (Ref) | | | 0.91 (0.86-0.96) | | 0.90 (0.85-0.94) | | 0.93 (0.88-0.98) | | | | 0.91 (0.85-0.97) | | 0.0026 |
|  | | | | HR (95%-CI), Model 1 | | | 1.00 (Ref) | | | 0.96 (0.91-1.01) | | 0.95 (0.90-1.00) | | 0.97 (0.91-1.02) | | | | 0.95 (0.90-1.02) | | 0.1607 |
|  | | | | HR (95%-CI), Model 2 | | | 1.00 (Ref) | | | 0.97 (0.92-1.02) | | 0.96 (0.91-1.01) | | 0.97 (0.92-1.03) | | | | 0.96 (0.90-1.03) | | 0.2396 |
|  | | | | HR (95%-CI), Model 3 | | | 1.00 (Ref) | | | 0.96 (0.91-1.01) | | 0.95 (0.90-1.00) | | 0.95 (0.90-1.01) | | | | 0.94 (0.88-1.00) | | 0.0603 |
|  | | | |  | | |  | | |  | |  | |  | | | |  | |  |
| Cellulitis | | | | Mean grams/day | | | 33.78 (43.99) | | | 84.71 (46.95) | | 99.17 (48.69) | | 116.04 (53.03) | | | |  | |  |
|  | | | | **Total n/cases** | | | 57543/368 | | | 118312/789 | | 152318/1140 | | 135086/1104 | | | | 463259/3401 | |  |
|  | | | | HR (95%-CI), Model 0 | | | 1.00 (Ref) | | | 0.96 (0.85-1.09) | | 1.07 (0.95-1.20) | | 1.12 (1.00-1.27) | | | | 1.20 (1.03-1.38) | | 0.0157 |
|  | | | | HR (95%-CI), Model 1 | | | 1.00 (Ref) | | | 1.00 (0.88-1.14) | | 1.13 (1.00-1.27) | | 1.16 (1.03-1.31) | | | | 1.24 (1.07-1.43) | | 0.0040 |
|  | | | | HR (95%-CI), Model 2 | | | 1.00 (Ref) | | | 1.03 (0.91-1.17) | | 1.16 (1.02-1.31) | | 1.18 (1.05-1.34) | | | | 1.27 (1.10-1.47) | | 0.0015 |
|  | | | | HR (95%-CI), Model 3 | | | 1.00 (Ref) | | | 0.95 (0.83-1.07) | | 1.02 (0.90-1.15) | | 0.99 (0.88-1.12) | | | | 1.02 (0.88-1.18) | | 0.8347 |
| **Model 0:** Stratified for sex, age group and region and adjusted for age (underlying time variable). **Model 1:** Model 0 additionally adjusted for race (4 groups where possible: White, Asian or Asian British, Black or Black British, Mixed race or other, unknown), deprivation (Townsend index quintiles, unknown), qualification (College or university degree/vocational qualification, National examination at ages 17-18,National examination at age 16, unknown), employment (in paid employment, receiving pension, not in paid employment, unknown), smoking (never, former, current <15 cigarettes/day, current >15 cigarettes/ day, current unknown amount of cigarettes/day, unknown), physical activity (<10 excess METs, 10-<50 excess METs, ≥ 50 excess METs, unknown), alcohol intake (none, < 1 g/day, 1-<10 g, 10-<20 g, ≥20 g/day, unknown), menopausal status (pre-, postmenopausal unknown), hormone-replacement therapy (HRT) (never, past, current, unknown) and oral contraceptive pill intake (OCP) (never, past, current, unknown), and parity (nulliparous, 1-2, 3 or more, unknown). **Model 2:** Model 1 additionally adjusted for total fruit and vegetable intake (<3 servings/day, 3-<4 servings/day, 4-<5 servings/day, ≥6 servings/day, unknown), cereal fibre intake (sex-specific quintiles, unknown), oily fish intake (0 times/week, <1 time/week, 1 time/week, > 2times/week, unknown) and non-oily fish intake (<1 time/week, 1 times/week, > 2 times/week, unknown). **Model 3:** Model 2 additionally adjusted for BMI (sex-specific quintiles, unknown). | | | | | | | | | | | | | | | | | | | | |
| Additional file 1: Table 7. Risk of 25 common conditions by unprocessed red and processed meat intake in UK Biobank. | | | | | | | | | | | | | | | | | | | |  |
|  | | |  | | | Unprocessed red and processed meat intake (times/week) | | | | | | | | | | |  | |  |  |
| Disease | | | Model | | | ≤1 | | | 2 | | 3 - 4 | | ≥5 | | | | per 70g/day increase | | p-value |  |
| Ischemic heart disease | | | Mean grams/day | | | 10.36 (23.38) | | | 48.59 (39.06) | | 64.49 (42.58) | | 78.98 (47.41) | | | |  | |  |  |
|  | | | Total n/cases | | | 41231/890 | | | 148286/3845 | | 129352/4156 | | 111845/4229 | | | | 430714/13120 | |  |  |
|  | | | HR (95%-CI), Model 0 | | | 1.00 (Ref) | | | 0.99 (0.92-1.07) | | 1.10 (1.02-1.18) | | 1.15 (1.07-1.24) | | | | 1.23 (1.15-1.32) | | <0.0001 |  |
|  | | | HR (95%-CI), Model 1 | | | 1.00 (Ref) | | | 1.08 (1.00-1.17) | | 1.18 (1.10-1.27) | | 1.22 (1.13-1.31) | | | | 1.26 (1.17-1.35) | | <0.0001 |  |
|  | | | HR (95%-CI), Model 2 | | | 1.00 (Ref) | | | 1.10 (1.02-1.19) | | 1.20 (1.11-1.29) | | 1.23 (1.14-1.33) | | | | 1.26 (1.17-1.35) | | <0.0001 |  |
|  | | | HR (95%-CI), Model 3 | | | 1.00 (Ref) | | | 1.04 (0.97-1.13) | | 1.11 (1.03-1.20) | | 1.12 (1.04-1.21) | | | | 1.15 (1.07-1.23) | | 0.0001 |  |
|  | | |  | | |  | | |  | |  | |  | | | |  | |  |  |
| Atrial Fibrillation and flutter | | | Mean grams/day | | | 10.38 (23.46) | | | 48.58 (39.06) | | 64.56 (42.63) | | 78.96 (47.45) | | | |  | |  |  |
|  | | | Total n/cases | | | 41231/369 | | | 148286/1487 | | 129352/1493 | | 111845/1392 | | | | 430714/4741 | |  |  |
|  | | | HR (95%-CI), Model 0 | | | 1.00 (Ref) | | | 0.93 (0.83-1.04) | | 0.98 (0.88-1.10) | | 0.97 (0.86-1.09) | | | | 1.01 (0.90-1.13) | | 0.8658 |  |
|  | | | HR (95%-CI), Model 1 | | | 1.00 (Ref) | | | 0.90 (0.80-1.01) | | 0.94 (0.83-1.05) | | 0.91 (0.81-1.03) | | | | 0.95 (0.85-1.06) | | 0.3539 |  |
|  | | | HR (95%-CI), Model 2 | | | 1.00 (Ref) | | | 0.93 (0.83-1.05) | | 0.98 (0.87-1.10) | | 0.95 (0.84-1.07) | | | | 0.99 (0.88-1.11) | | 0.8155 |  |
|  | | | HR (95%-CI), Model 3 | | | 1.00 (Ref) | | | 0.88 (0.78-0.99) | | 0.90 (0.80-1.02) | | 0.86 (0.76-0.97) | | | | 0.89 (0.79-0.99) | | 0.0396 |  |
|  | | |  | | |  | | |  | |  | |  | | | |  | |  |  |
| Cerebrovascular | | | Mean grams/day | | | 10.39 (23.46) | | | 48.58 (39.05) | | 64.53 (42.62) | | 78.95 (47.42) | | | |  | |  |  |
|  | | | Total n/cases | | | 41231/377 | | | 148286/1687 | | 129352/1625 | | 111845/1641 | | | | 430714/5330 | |  |  |
|  | | | HR (95%-CI), Model 0 | | | 1.00 (Ref) | | | 1.06 (0.95-1.19) | | 1.11 (1.00-1.25) | | 1.23 (1.09-1.37) | | | | 1.26 (1.13-1.40) | | <0.0001 |  |
|  | | | HR (95%-CI), Model 1 | | | 1.00 (Ref) | | | 1.09 (0.98-1.22) | | 1.12 (1.00-1.26) | | 1.20 (1.07-1.35) | | | | 1.20 (1.08-1.34) | | 0.0007 |  |
|  | | | HR (95%-CI), Model 2 | | | 1.00 (Ref) | | | 1.11 (0.99-1.25) | | 1.14 (1.02-1.29) | | 1.21 (1.08-1.36) | | | | 1.21 (1.09-1.35) | | 0.0006 |  |
|  | | | HR (95%-CI), Model 3 | | | 1.00 (Ref) | | | 1.09 (0.97-1.23) | | 1.11 (0.99-1.25) | | 1.17 (1.04-1.32) | | | | 1.17 (1.04-1.30) | | 0.0063 |  |
|  | | |  | | |  | | |  | |  | |  | | | |  | |  |  |
| Ischaemic stroke | | | Mean grams/day | | | 10.38 (23.45) | | | 48.57 (39.04) | | 64.53 (42.62) | | 78.96 (47.42) | | | |  | |  |  |
|  | | | Total n/cases | | | 41231/153 | | | 148286/695 | | 129352/720 | | 111845/770 | | | | 430714/2338 | |  |  |
|  | | | HR (95%-CI), Model 0 | | | 1.00 (Ref) | | | 1.04 (0.87-1.24) | | 1.14 (0.96-1.36) | | 1.31 (1.10-1.56) | | | | 1.41 (1.19-1.66) | | 0.0001 |  |
|  | | | HR (95%-CI), Model 1 | | | 1.00 (Ref) | | | 1.09 (0.92-1.30) | | 1.17 (0.98-1.40) | | 1.29 (1.08-1.54) | | | | 1.34 (1.13-1.58) | | 0.0006 |  |
|  | | | HR (95%-CI), Model 2 | | | 1.00 (Ref) | | | 1.13 (0.95-1.36) | | 1.21 (1.01-1.45) | | 1.32 (1.10-1.58) | | | | 1.35 (1.14-1.60) | | 0.0005 |  |
|  | | | HR (95%-CI), Model 3 | | | 1.00 (Ref) | | | 1.09 (0.91-1.31) | | 1.15 (0.96-1.38) | | 1.23 (1.02-1.48) | | | | 1.26 (1.06-1.49) | | 0.0082 |  |
|  | | |  | | |  | | |  | |  | |  | | | |  | |  |  |
| Haemorrhagic stroke | | | Mean grams/day | | | 10.38 (23.45) | | | 48.57 (39.04) | | 64.54 (42.62) | | 78.95 (47.43) | | | |  | |  |  |
|  | | | Total n/cases | | | 41231/80 | | | 148286/320 | | 129352/279 | | 111845/260 | | | | 430714/939 | |  |  |
|  | | | HR (95%-CI), Model 0 | | | 1.00 (Ref) | | | 1.02 (0.80-1.31) | | 1.03 (0.80-1.32) | | 1.11 (0.86-1.43) | | | | 1.11 (0.87-1.42) | | 0.4006 |  |
|  | | | HR (95%-CI), Model 1 | | | 1.00 (Ref) | | | 1.04 (0.81-1.34) | | 1.03 (0.80-1.33) | | 1.09 (0.84-1.41) | | | | 1.08 (0.84-1.38) | | 0.5568 |  |
|  | | | HR (95%-CI), Model 2 | | | 1.00 (Ref) | | | 1.07 (0.83-1.37) | | 1.05 (0.81-1.37) | | 1.10 (0.85-1.44) | | | | 1.09 (0.85-1.40) | | 0.5081 |  |
|  | | | HR (95%-CI), Model 3 | | | 1.00 (Ref) | | | 1.09 (0.84-1.40) | | 1.08 (0.83-1.40) | | 1.13 (0.87-1.48) | | | | 1.12 (0.87-1.44) | | 0.3778 |  |
|  | | |  | | |  | | |  | |  | |  | | | |  | |  |  |
| Venous thromboembolism | | | Mean grams/day | | | 10.51 (23.57) | | | 48.65 (39.05) | | 64.95 (42.82) | | 79.21 (47.58) | | | |  | |  |  |
|  | | | Total n/cases | | | 43170/255 | | | 156616/1211 | | 137523/1186 | | 120091/1099 | | | | 457400/3751 | |  |  |
|  | | | HR (95%-CI), Model 0 | | | 1.00 (Ref) | | | 1.16 (1.01-1.33) | | 1.23 (1.07-1.41) | | 1.24 (1.08-1.42) | | | | 1.23 (1.08-1.39) | | 0.0016 |  |
|  | | | HR (95%-CI), Model 1 | | | 1.00 (Ref) | | | 1.17 (1.02-1.34) | | 1.23 (1.08-1.42) | | 1.23 (1.07-1.41) | | | | 1.21 (1.07-1.38) | | 0.0031 |  |
|  | | | HR (95%-CI), Model 2 | | | 1.00 (Ref) | | | 1.19 (1.04-1.37) | | 1.26 (1.09-1.44) | | 1.24 (1.08-1.44) | | | | 1.22 (1.07-1.38) | | 0.0030 |  |
|  | | | HR (95%-CI), Model 3 | | | 1.00 (Ref) | | | 1.11 (0.97-1.28) | | 1.13 (0.99-1.31) | | 1.10 (0.95-1.27) | | | | 1.07 (0.94-1.22) | | 0.3031 |  |
|  | | |  | | |  | | |  | |  | |  | | | |  | |  |  |
| Varicose veins | | | Mean grams/day | | | 10.50 (23.54) | | | 48.82 (39.17) | | 64.98 (42.78) | | 79.41 (47.60) | | | |  | |  |  |
|  | | | Total n/cases | | | 42006/251 | | | 151715/929 | | 134036/794 | | 117747/659 | | | | 445504/2633 | |  |  |
|  | | | HR (95%-CI), Model 0 | | | 1.00 (Ref) | | | 1.03 (0.89-1.18) | | 1.02 (0.88-1.17) | | 0.97 (0.84-1.13) | | | | 0.97 (0.84-1.11) | | 0.6401 |  |
|  | | | HR (95%-CI), Model 1 | | | 1.00 (Ref) | | | 1.03 (0.89-1.18) | | 1.01 (0.87-1.17) | | 0.96 (0.83-1.12) | | | | 0.95 (0.83-1.10) | | 0.4996 |  |
|  | | | HR (95%-CI), Model 2 | | | 1.00 (Ref) | | | 1.05 (0.91-1.21) | | 1.04 (0.90-1.21) | | 1.00 (0.86-1.16) | | | | 0.99 (0.85-1.14) | | 0.8623 |  |
|  | | | HR (95%-CI), Model 3 | | | 1.00 (Ref) | | | 1.02 (0.88-1.18) | | 1.00 (0.86-1.16) | | 0.95 (0.81-1.11) | | | | 0.94 (0.81-1.09) | | 0.3907 |  |
|  | | |  | | |  | | |  | |  | |  | | | |  | |  |  |
| Haemorrhoids | | | Mean grams/day | | | 10.48 (23.51) | | | 48.71 (39.06) | | 64.81 (42.69) | | 79.17 (47.56) | | | |  | |  |  |
|  | | | Total n/cases | | | 42327/761 | | | 153619/2801 | | 134780/2471 | | 117394/2263 | | | | 448120/8296 | |  |  |
|  | | | HR (95%-CI), Model 0 | | | 1.00 (Ref) | | | 1.00 (0.93-1.09) | | 1.00 (0.92-1.08) | | 1.04 (0.95-1.13) | | | | 1.04 (0.96-1.12) | | 0.4014 |  |
|  | | | HR (95%-CI), Model 1 | | | 1.00 (Ref) | | | 1.03 (0.95-1.11) | | 1.02 (0.94-1.11) | | 1.06 (0.98-1.16) | | | | 1.06 (0.98-1.15) | | 0.1580 |  |
|  | | | HR (95%-CI), Model 2 | | | 1.00 (Ref) | | | 1.04 (0.95-1.13) | | 1.03 (0.95-1.13) | | 1.08 (0.99-1.17) | | | | 1.07 (0.99-1.16) | | 0.1062 |  |
|  | | | HR (95%-CI), Model 3 | | | 1.00 (Ref) | | | 1.04 (0.95-1.13) | | 1.03 (0.95-1.13) | | 1.08 (0.99-1.18) | | | | 1.07 (0.99-1.17) | | 0.0983 |  |
|  | | |  | | |  | | |  | |  | |  | | | |  | |  |  |
| Pneumonia | | | Mean grams/day | | | 10.52 (23.60) | | | 48.78 (39.05) | | 64.81 (42.67) | | 79.19 (47.65) | | | |  | |  |  |
|  | | | Total n/cases | | | 43323/462 | | | 157345/1885 | | 138154/1935 | | 120487/2068 | | | | 459309/6350 | |  |  |
|  | | | HR (95%-CI), Model 0 | | | 1.00 (Ref) | | | 0.99 (0.89-1.09) | | 1.11 (1.00-1.23) | | 1.30 (1.17-1.44) | | | | 1.43 (1.29-1.58) | | <0.0001 |  |
|  | | | HR (95%-CI), Model 1 | | | 1.00 (Ref) | | | 1.06 (0.95-1.17) | | 1.15 (1.03-1.27) | | 1.28 (1.15-1.42) | | | | 1.34 (1.22-1.48) | | <0.0001 |  |
|  | | | HR (95%-CI), Model 2 | | | 1.00 (Ref) | | | 1.09 (0.98-1.21) | | 1.17 (1.05-1.30) | | 1.30 (1.17-1.44) | | | | 1.35 (1.22-1.49) | | <0.0001 |  |
|  | | | HR (95%-CI), Model 3 | | | 1.00 (Ref) | | | 1.09 (0.98-1.21) | | 1.16 (1.04-1.29) | | 1.27 (1.14-1.41) | | | | 1.31 (1.18-1.44) | | <0.0001 |  |
|  | | |  | | |  | | |  | |  | |  | | | |  | |  |  |
| Gastro-oesophageal reflux disease | | | Mean grams/day | | | 10.40 (23.48) | | | 48.61 (39.10) | | 64.82 (42.71) | | 79.20 (47.37) | | | |  | |  |  |
|  | | | Total n/cases | | | 41762/661 | | | 150750/2426 | | 132266/2109 | | 115078/1814 | | | | 439856/7010 | |  |  |
|  | | | HR (95%-CI), Model 0 | | | 1.00 (Ref) | | | 0.98 (0.90-1.07) | | 0.99 (0.90-1.08) | | 0.98 (0.90-1.08) | | | | 0.99 (0.91-1.08) | | 0.7988 |  |
|  | | | HR (95%-CI), Model 1 | | | 1.00 (Ref) | | | 1.00 (0.92-1.09) | | 1.00 (0.92-1.10) | | 1.00 (0.91-1.09) | | | | 1.00 (0.92-1.09) | | 0.9970 |  |
|  | | | HR (95%-CI), Model 2 | | | 1.00 (Ref) | | | 1.02 (0.93-1.12) | | 1.02 (0.93-1.12) | | 1.01 (0.92-1.11) | | | | 1.01 (0.92-1.10) | | 0.8705 |  |
|  | | | HR (95%-CI), Model 3 | | | 1.00 (Ref) | | | 1.00 (0.91-1.09) | | 0.99 (0.90-1.09) | | 0.98 (0.89-1.08) | | | | 0.97 (0.89-1.07) | | 0.5656 |  |
|  | | |  | | |  | | |  | |  | |  | | | |  | |  |  |
| Gastritis and duodenitis | | | Mean grams/day | | | 10.42 (23.43) | | | 48.75 (39.15) | | 64.83 (42.76) | | 79.20 (47.57) | | | |  | |  |  |
|  | | | Total n/cases | | | 42476/1151 | | | 154550/3889 | | 135671/3352 | | 118300/2973 | | | | 450997/11365 | |  |  |
|  | | | HR (95%-CI), Model 0 | | | 1.00 (Ref) | | | 0.91 (0.85-0.97) | | 0.92 (0.86-0.99) | | 0.95 (0.88-1.02) | | | | 0.97 (0.90-1.03) | | 0.3197 |  |
|  | | | HR (95%-CI), Model 1 | | | 1.00 (Ref) | | | 0.97 (0.91-1.04) | | 0.97 (0.91-1.04) | | 0.99 (0.92-1.06) | | | | 0.99 (0.93-1.06) | | 0.8521 |  |
|  | | | HR (95%-CI), Model 2 | | | 1.00 (Ref) | | | 0.99 (0.93-1.06) | | 0.99 (0.93-1.06) | | 1.00 (0.93-1.08) | | | | 1.00 (0.94-1.08) | | 0.9380 |  |
|  | | | HR (95%-CI), Model 3 | | | 1.00 (Ref) | | | 0.98 (0.92-1.05) | | 0.98 (0.92-1.05) | | 0.99 (0.92-1.06) | | | | 0.99 (0.92-1.06) | | 0.7341 |  |
|  | | |  | | |  | | |  | |  | |  | | | |  | |  |  |
| Inguinal hernia | | | Mean grams/day | | | 10.56 (23.66) | | | 48.71 (38.99) | | 64.71 (42.64) | | 79.35 (47.71) | | | |  | |  |  |
|  | | | Total n/cases | | | 42328/521 | | | 152026/2388 | | 132074/2531 | | 114332/2579 | | | | 440760/8019 | |  |  |
|  | | | HR (95%-CI), Model 0 | | | 1.00 (Ref) | | | 0.97 (0.88-1.07) | | 0.91 (0.83-1.01) | | 0.87 (0.79-0.95) | | | | 0.84 (0.77-0.91) | | <0.0001 |  |
|  | | | HR (95%-CI), Model 1 | | | 1.00 (Ref) | | | 0.95 (0.86-1.04) | | 0.90 (0.82-0.99) | | 0.87 (0.79-0.95) | | | | 0.85 (0.78-0.93) | | 0.0002 |  |
|  | | | HR (95%-CI), Model 2 | | | 1.00 (Ref) | | | 0.95 (0.86-1.05) | | 0.91 (0.82-1.00) | | 0.88 (0.80-0.97) | | | | 0.86 (0.79-0.94) | | 0.0010 |  |
|  | | | HR (95%-CI), Model 3 | | | 1.00 (Ref) | | | 1.03 (0.93-1.14) | | 1.01 (0.92-1.12) | | 1.01 (0.91-1.11) | | | | 0.99 (0.90-1.08) | | 0.7619 |  |
|  | | |  | | |  | | |  | |  | |  | | | |  | |  |  |
| Noninfective enteritis and colitis | | | Mean grams/day | | | 10.42 (23.50) | | | 48.70 (39.13) | | 64.86 (42.77) | | 79.43 (47.74) | | | |  | |  |  |
|  | | | Total n/cases | | | 42589/599 | | | 155326/2108 | | 136439/1865 | | 119262/1663 | | | | 453616/6235 | |  |  |
|  | | | HR (95%-CI), Model 0 | | | 1.00 (Ref) | | | 0.95 (0.86-1.04) | | 0.98 (0.89-1.08) | | 1.03 (0.94-1.14) | | | | 1.06 (0.97-1.16) | | 0.2096 |  |
|  | | | HR (95%-CI), Model 1 | | | 1.00 (Ref) | | | 0.97 (0.89-1.06) | | 1.00 (0.91-1.10) | | 1.04 (0.94-1.14) | | | | 1.05 (0.96-1.16) | | 0.2661 |  |
|  | | | HR (95%-CI), Model 2 | | | 1.00 (Ref) | | | 1.00 (0.91-1.10) | | 1.03 (0.93-1.13) | | 1.06 (0.96-1.17) | | | | 1.07 (0.98-1.18) | | 0.1449 |  |
|  | | | HR (95%-CI), Model 3 | | | 1.00 (Ref) | | | 0.99 (0.90-1.09) | | 1.01 (0.92-1.12) | | 1.04 (0.94-1.15) | | | | 1.05 (0.96-1.16) | | 0.3036 |  |
|  | | |  | | |  | | |  | |  | |  | | | |  | |  |  |
| Diverticular disease | | | Mean grams/day | | | 10.44 (23.56) | | | 48.71 (39.17) | | 64.76 (42.76) | | 79.22 (47.71) | | | |  | |  |  |
|  | | | Total n/cases | | | 43123/943 | | | 155929/4246 | | 136813/3998 | | 119416/3629 | | | | 455281/12816 | |  |  |
|  | | | HR (95%-CI), Model 0 | | | 1.00 (Ref) | | | 1.17 (1.09-1.25) | | 1.28 (1.19-1.38) | | 1.33 (1.24-1.43) | | | | 1.36 (1.27-1.45) | | <0.0001 |  |
|  | | | HR (95%-CI), Model 1 | | | 1.00 (Ref) | | | 1.13 (1.05-1.22) | | 1.23 (1.15-1.32) | | 1.27 (1.18-1.37) | | | | 1.29 (1.21-1.38) | | <0.0001 |  |
|  | | | HR (95%-CI), Model 2 | | | 1.00 (Ref) | | | 1.14 (1.06-1.23) | | 1.24 (1.15-1.33) | | 1.27 (1.18-1.37) | | | | 1.28 (1.20-1.37) | | <0.0001 |  |
|  | | | HR (95%-CI), Model 3 | | | 1.00 (Ref) | | | 1.09 (1.02-1.18) | | 1.17 (1.08-1.25) | | 1.18 (1.09-1.27) | | | | 1.19 (1.11-1.28) | | <0.0001 |  |
|  | | |  | | |  | | |  | |  | |  | | | |  | |  |  |
| Colon polyps | | | Mean grams/day | | | 10.46 (23.59) | | | 48.71 (39.14) | | 64.93 (42.83) | | 79.40 (47.58) | | | |  | |  |  |
|  | | | Total n/cases | | | 42076/3108 | | | 152858/12436 | | 134197/11349 | | 116917/10507 | | | | 446048/37400 | |  |  |
|  | | | HR (95%-CI), Model 0 | | | 1.00 (Ref) | | | 1.04 (1.00-1.08) | | 1.09 (1.04-1.13) | | 1.15 (1.11-1.20) | | | | 1.17 (1.13-1.22) | | <0.0001 |  |
|  | | | HR (95%-CI), Model 1 | | | 1.00 (Ref) | | | 1.04 (1.00-1.08) | | 1.07 (1.03-1.12) | | 1.13 (1.08-1.17) | | | | 1.14 (1.10-1.18) | | <0.0001 |  |
|  | | | HR (95%-CI), Model 2 | | | 1.00 (Ref) | | | 1.05 (1.01-1.09) | | 1.08 (1.04-1.13) | | 1.13 (1.09-1.18) | | | | 1.14 (1.10-1.19) | | <0.0001 |  |
|  | | | HR (95%-CI), Model 3 | | | 1.00 (Ref) | | | 1.03 (0.99-1.07) | | 1.05 (1.01-1.10) | | 1.09 (1.05-1.14) | | | | 1.10 (1.06-1.15) | | <0.0001 |  |
|  | | |  | | |  | | |  | |  | |  | | | |  | |  |  |
| Gallbladder disease | | | Mean grams/day | | | 10.40 (23.52) | | | 48.62 (39.08) | | 64.80 (42.80) | | 79.32 (47.73) | | | |  | |  |  |
|  | | | Total n/cases | | | 42304/767 | | | 152826/3125 | | 134818/2695 | | 118434/2327 | | | | 448382/8914 | |  |  |
|  | | | HR (95%-CI), Model 0 | | | 1.00 (Ref) | | | 1.14 (1.05-1.23) | | 1.19 (1.09-1.29) | | 1.24 (1.14-1.35) | | | | 1.24 (1.15-1.34) | | <0.0001 |  |
|  | | | HR (95%-CI), Model 1 | | | 1.00 (Ref) | | | 1.15 (1.06-1.25) | | 1.21 (1.11-1.31) | | 1.27 (1.17-1.39) | | | | 1.27 (1.17-1.37) | | <0.0001 |  |
|  | | | HR (95%-CI), Model 2 | | | 1.00 (Ref) | | | 1.18 (1.09-1.28) | | 1.23 (1.13-1.33) | | 1.29 (1.18-1.41) | | | | 1.27 (1.17-1.38) | | <0.0001 |  |
|  | | | HR (95%-CI), Model 3 | | | 1.00 (Ref) | | | 1.06 (0.97-1.15) | | 1.04 (0.96-1.13) | | 1.06 (0.97-1.15) | | | | 1.04 (0.96-1.13) | | 0.3259 |  |
|  | | |  | | |  | | |  | |  | |  | | | |  | |  |  |
| Osteoarthritis | | | Mean grams/day | | | 10.31 (23.69) | | | 48.69 (39.16) | | 64.59 (42.59) | | 79.11 (47.60) | | | |  | |  |  |
|  | | | Total n/cases | | | 39814/1451 | | | 142735/6220 | | 125452/5576 | | 109175/4833 | | | | 417176/18080 | |  |  |
|  | | | HR (95%-CI), Model 0 | | | 1.00 (Ref) | | | 1.09 (1.03-1.15) | | 1.14 (1.07-1.21) | | 1.15 (1.09-1.22) | | | | 1.16 (1.09-1.22) | | <0.0001 |  |
|  | | | HR (95%-CI), Model 1 | | | 1.00 (Ref) | | | 1.08 (1.02-1.14) | | 1.11 (1.05-1.18) | | 1.12 (1.05-1.19) | | | | 1.12 (1.06-1.18) | | 0.0001 |  |
|  | | | HR (95%-CI), Model 2 | | | 1.00 (Ref) | | | 1.10 (1.04-1.17) | | 1.15 (1.08-1.22) | | 1.15 (1.08-1.23) | | | | 1.15 (1.09-1.22) | | <0.0001 |  |
|  | | | HR (95%-CI), Model 3 | | | 1.00 (Ref) | | | 1.00 (0.95-1.06) | | 1.00 (0.94-1.06) | | 0.97 (0.92-1.04) | | | | 0.97 (0.92-1.03) | | 0.3110 |  |
|  | | |  | | |  | | |  | |  | |  | | | |  | |  |  |
| Kidney stones | | | Mean grams/day | | | 10.49 (23.56) | | | 48.70 (39.08) | | 64.76 (42.70) | | 79.18 (47.54) | | | |  | |  |  |
|  | | | Total n/cases | | | 43491/190 | | | 157886/765 | | 138388/772 | | 120794/717 | | | | 460559/2444 | |  |  |
|  | | | HR (95%-CI), Model 0 | | | 1.00 (Ref) | | | 1.04 (0.89-1.22) | | 1.09 (0.93-1.28) | | 1.06 (0.90-1.25) | | | | 1.07 (0.92-1.24) | | 0.4096 |  |
|  | | | HR (95%-CI), Model 1 | | | 1.00 (Ref) | | | 1.12 (0.95-1.31) | | 1.19 (1.01-1.39) | | 1.17 (0.99-1.38) | | | | 1.17 (1.00-1.36) | | 0.0455 |  |
|  | | | HR (95%-CI), Model 2 | | | 1.00 (Ref) | | | 1.11 (0.94-1.31) | | 1.16 (0.99-1.37) | | 1.13 (0.96-1.34) | | | | 1.13 (0.96-1.32) | | 0.1324 |  |
|  | | | HR (95%-CI), Model 3 | | | 1.00 (Ref) | | | 1.05 (0.89-1.23) | | 1.07 (0.91-1.27) | | 1.03 (0.87-1.22) | | | | 1.02 (0.87-1.19) | | 0.7971 |  |
|  | | |  | | |  | | |  | |  | |  | | | |  | |  |  |
| Urinary tract infection | | | Mean grams/day | | | 10.55 (23.66) | | | 48.80 (39.13) | | 64.82 (42.71) | | 79.30 (47.70) | | | |  | |  |  |
|  | | | Total n/cases | | | 43172/465 | | | 157448/1647 | | 138319/1582 | | 121049/1511 | | | | 459988/5205 | |  |  |
|  | | | HR (95%-CI), Model 0 | | | 1.00 (Ref) | | | 0.88 (0.80-0.98) | | 0.96 (0.86-1.06) | | 1.02 (0.92-1.13) | | | | 1.09 (0.98-1.21) | | 0.1040 |  |
|  | | | HR (95%-CI), Model 1 | | | 1.00 (Ref) | | | 0.95 (0.85-1.05) | | 1.02 (0.92-1.13) | | 1.08 (0.97-1.20) | | | | 1.13 (1.02-1.26) | | 0.0215 |  |
|  | | | HR (95%-CI), Model 2 | | | 1.00 (Ref) | | | 0.98 (0.88-1.09) | | 1.05 (0.94-1.17) | | 1.10 (0.99-1.23) | | | | 1.15 (1.03-1.28) | | 0.0106 |  |
|  | | | HR (95%-CI), Model 3 | | | 1.00 (Ref) | | | 0.95 (0.85-1.05) | | 1.00 (0.89-1.11) | | 1.03 (0.92-1.15) | | | | 1.07 (0.96-1.19) | | 0.2189 |  |
|  | | |  | | |  | | |  | |  | |  | | | |  | |  |  |
| Enlarged prostate | | | Mean grams/day | | | 10.80 (26.01) | | | 53.46 (42.54) | | 70.13 (44.95) | | 85.35 (50.06) | | | |  | |  |  |
|  | | | Total n/cases | | | 12148/201 | | | 55481/972 | | 65868/1172 | | 72093/1194 | | | | 205590/3539 | |  |  |
|  | | | HR (95%-CI), Model 0 | | | 1.00 (Ref) | | | 0.94 (0.80-1.09) | | 0.96 (0.83-1.12) | | 0.88 (0.76-1.02) | | | | 0.89 (0.78-1.01) | | 0.0708 |  |
|  | | | HR (95%-CI), Model 1 | | | 1.00 (Ref) | | | 1.00 (0.86-1.17) | | 1.05 (0.90-1.23) | | 0.98 (0.84-1.15) | | | | 0.99 (0.86-1.13) | | 0.8517 |  |
|  | | | HR (95%-CI), Model 2 | | | 1.00 (Ref) | | | 1.02 (0.87-1.19) | | 1.07 (0.92-1.25) | | 1.00 (0.86-1.17) | | | | 1.00 (0.87-1.15) | | 0.9732 |  |
|  | | | HR (95%-CI), Model 3 | | | 1.00 (Ref) | | | 1.02 (0.87-1.19) | | 1.08 (0.92-1.26) | | 1.01 (0.86-1.18) | | | | 1.01 (0.88-1.16) | | 0.8927 |  |
|  | | |  | | |  | | |  | |  | |  | | | |  | |  |  |
| Female genital prolapse | | | Mean grams/day | | | 10.37 (22.63) | | | 46.03 (36.86) | | 60.08 (40.13) | | 70.41 (42.55) | | | |  | |  |  |
|  | | | Total n/cases | | | 30054/616 | | | 96685/2457 | | 67921/1747 | | 44994/1242 | | | | 239654/6062 | |  |  |
|  | | | HR (95%-CI), Model 0 | | | 1.00 (Ref) | | | 1.17 (1.07-1.27) | | 1.17 (1.07-1.29) | | 1.26 (1.14-1.39) | | | | 1.23 (1.12-1.35) | | <0.0001 |  |
|  | | | HR (95%-CI), Model 1 | | | 1.00 (Ref) | | | 1.08 (0.99-1.18) | | 1.07 (0.97-1.17) | | 1.14 (1.04-1.26) | | | | 1.12 (1.02-1.23) | | 0.0190 |  |
|  | | | HR (95%-CI), Model 2 | | | 1.00 (Ref) | | | 1.11 (1.02-1.22) | | 1.10 (1.00-1.21) | | 1.19 (1.07-1.31) | | | | 1.16 (1.05-1.27) | | 0.0023 |  |
|  | | | HR (95%-CI), Model 3 | | | 1.00 (Ref) | | | 1.07 (0.98-1.17) | | 1.05 (0.95-1.15) | | 1.12 (1.01-1.23) | | | | 1.09 (0.99-1.20) | | 0.0742 |  |
|  | | |  | | |  | | |  | |  | |  | | | |  | |  |  |
| Uterine fibroids | | | Mean grams/day | | | 9.97 (22.19) | | | 45.58 (36.74) | | 60.02 (40.04) | | 70.23 (42.28) | | | |  | |  |  |
|  | | | Total n/cases | | | 23395/1191 | | | 73089/3709 | | 51367/2595 | | 33935/1763 | | | | 181786/9258 | |  |  |
|  | | | HR (95%-CI), Model 0 | | | 1.00 (Ref) | | | 1.05 (0.98-1.12) | | 1.04 (0.97-1.11) | | 1.07 (0.99-1.15) | | | | 1.06 (0.98-1.13) | | 0.1257 |  |
|  | | | HR (95%-CI), Model 1 | | | 1.00 (Ref) | | | 1.07 (1.00-1.14) | | 1.06 (0.99-1.14) | | 1.09 (1.01-1.17) | | | | 1.08 (1.00-1.15) | | 0.0416 |  |
|  | | | HR (95%-CI), Model 2 | | | 1.00 (Ref) | | | 1.08 (1.01-1.16) | | 1.07 (1.00-1.15) | | 1.10 (1.02-1.19) | | | | 1.08 (1.01-1.16) | | 0.0299 |  |
|  | | | HR (95%-CI), Model 3 | | | 1.00 (Ref) | | | 1.03 (0.96-1.11) | | 1.00 (0.93-1.07) | | 1.00 (0.93-1.09) | | | | 0.99 (0.92-1.06) | | 0.7762 |  |
|  | | |  | | |  | | |  | |  | |  | | | |  | |  |  |
| Iron deficiency anaemia | | | Mean grams/day | | | 10.47 (23.43) | | | 48.75 (39.14) | | 64.87 (42.72) | | 79.38 (47.69) | | | |  | |  |  |
|  | | | Total n/cases | | | 43107/583 | | | 158115/1560 | | 139150/1418 | | 121730/1158 | | | | 462102/4719 | |  |  |
|  | | | HR (95%-CI), Model 0 | | | 1.00 (Ref) | | | 0.69 (0.63-0.76) | | 0.73 (0.66-0.81) | | 0.70 (0.63-0.77) | | | | 0.74 (0.66-0.81) | | <0.0001 |  |
|  | | | HR (95%-CI), Model 1 | | | 1.00 (Ref) | | | 0.80 (0.73-0.89) | | 0.86 (0.78-0.95) | | 0.82 (0.74-0.91) | | | | 0.86 (0.78-0.95) | | 0.0044 |  |
|  | | | HR (95%-CI), Model 2 | | | 1.00 (Ref) | | | 0.85 (0.77-0.94) | | 0.90 (0.81-1.00) | | 0.86 (0.77-0.96) | | | | 0.90 (0.81-0.99) | | 0.0384 |  |
|  | | | HR (95%-CI), Model 3 | | | 1.00 (Ref) | | | 0.83 (0.75-0.91) | | 0.87 (0.78-0.96) | | 0.82 (0.73-0.91) | | | | 0.85 (0.77-0.94) | | 0.0026 |  |
|  | | |  | | |  | | |  | |  | |  | | | |  | |  |  |
| Diabetes | | | Mean grams/day | | | 10.48 (23.57) | | | 48.47 (38.99) | | 64.52 (42.60) | | 78.65 (47.20) | | | |  | |  |  |
|  | | | Total n/cases | | | 42147/699 | | | 152899/2729 | | 132951/2972 | | 114743/3171 | | | | 442740/9571 | |  |  |
|  | | | HR (95%-CI), Model 0 | | | 1.00 (Ref) | | | 0.96 (0.89-1.05) | | 1.15 (1.06-1.25) | | 1.35 (1.25-1.47) | | | | 1.56 (1.43-1.69) | | <0.0001 |  |
|  | | | HR (95%-CI), Model 1 | | | 1.00 (Ref) | | | 1.17 (1.08-1.27) | | 1.39 (1.28-1.52) | | 1.60 (1.46-1.74) | | | | 1.73 (1.59-1.87) | | <0.0001 |  |
|  | | | HR (95%-CI), Model 2 | | | 1.00 (Ref) | | | 1.21 (1.11-1.31) | | 1.42 (1.30-1.55) | | 1.62 (1.48-1.76) | | | | 1.73 (1.59-1.88) | | <0.0001 |  |
|  | | | HR (95%-CI), Model 3 | | | 1.00 (Ref) | | | 1.05 (0.96-1.14) | | 1.15 (1.05-1.26) | | 1.23 (1.13-1.35) | | | | 1.30 (1.20-1.42) | | <0.0001 |  |
|  | | |  | | |  | | |  | |  | |  | | | |  | |  |  |
| Carpal tunnel syndrome | | | Mean grams/day | | | 10.42 (23.50) | | | 48.73 (39.11) | | 64.81 (42.65) | | 79.27 (47.62) | | | |  | |  |  |
|  | | | Total n/cases | | | 43183/439 | | | 156637/1752 | | 137949/1575 | | 120720/1282 | | | | 458489/5048 | |  |  |
|  | | | HR (95%-CI), Model 0 | | | 1.00 (Ref) | | | 1.10 (0.99-1.23) | | 1.20 (1.08-1.33) | | 1.19 (1.06-1.32) | | | | 1.21 (1.09-1.34) | | 0.0004 |  |
|  | | | HR (95%-CI), Model 1 | | | 1.00 (Ref) | | | 1.13 (1.02-1.26) | | 1.22 (1.10-1.36) | | 1.19 (1.06-1.33) | | | | 1.20 (1.08-1.33) | | 0.0007 |  |
|  | | | HR (95%-CI), Model 2 | | | 1.00 (Ref) | | | 1.16 (1.04-1.29) | | 1.26 (1.13-1.40) | | 1.22 (1.09-1.37) | | | | 1.23 (1.11-1.37) | | 0.0001 |  |
|  | | | HR (95%-CI), Model 3 | | | 1.00 (Ref) | | | 1.06 (0.95-1.18) | | 1.11 (0.99-1.24) | | 1.05 (0.93-1.17) | | | | 1.05 (0.95-1.17) | | 0.3492 |  |
|  | | |  | | |  | | |  | |  | |  | | | |  | |  |  |
| Cataracts | | | Mean grams/day | | | 10.46 (23.61) | | | 48.42 (38.96) | | 64.61 (42.65) | | 78.74 (47.24) | | | |  | |  |  |
|  | | | Total n/cases | | | 40484/1520 | | | 146968/5644 | | 128122/4764 | | 110397/4185 | | | | 425971/16113 | |  |  |
|  | | | HR (95%-CI), Model 0 | | | 1.00 (Ref) | | | 0.88 (0.83-0.93) | | 0.86 (0.81-0.92) | | 0.88 (0.83-0.94) | | | | 0.90 (0.85-0.95) | | 0.0002 |  |
|  | | | HR (95%-CI), Model 1 | | | 1.00 (Ref) | | | 0.94 (0.89-1.00) | | 0.93 (0.88-0.99) | | 0.94 (0.88-1.00) | | | | 0.94 (0.89-1.00) | | 0.0485 |  |
|  | | | HR (95%-CI), Model 2 | | | 1.00 (Ref) | | | 0.96 (0.90-1.01) | | 0.94 (0.88-1.00) | | 0.95 (0.89-1.01) | | | | 0.95 (0.89-1.01) | | 0.0818 |  |
|  | | | HR (95%-CI), Model 3 | | | 1.00 (Ref) | | | 0.95 (0.89-1.00) | | 0.93 (0.87-0.98) | | 0.93 (0.87-0.99) | | | | 0.93 (0.87-0.99) | | 0.0154 |  |
|  | | |  | | |  | | |  | |  | |  | | | |  | |  |  |
| Cellulitis | | | Mean grams/day | | | 10.53 (23.61) | | | 48.72 (39.11) | | 64.81 (42.66) | | 79.21 (47.66) | | | |  | |  |  |
|  | | | Total n/cases | | | 43698/287 | | | 158859/996 | | 139404/1096 | | 121648/1030 | | | | 463609/3409 | |  |  |
|  | | | HR (95%-CI), Model 0 | | | 1.00 (Ref) | | | 0.90 (0.78-1.02) | | 1.06 (0.93-1.21) | | 1.08 (0.95-1.24) | | | | 1.20 (1.06-1.37) | | 0.0057 |  |
|  | | | HR (95%-CI), Model 1 | | | 1.00 (Ref) | | | 0.95 (0.83-1.09) | | 1.11 (0.97-1.27) | | 1.11 (0.97-1.27) | | | | 1.20 (1.05-1.37) | | 0.0072 |  |
|  | | | HR (95%-CI), Model 2 | | | 1.00 (Ref) | | | 0.98 (0.86-1.12) | | 1.15 (1.01-1.32) | | 1.14 (1.00-1.31) | | | | 1.23 (1.08-1.41) | | 0.0020 |  |
|  | | | HR (95%-CI), Model 3 | | | 1.00 (Ref) | | | 0.89 (0.77-1.02) | | 1.00 (0.87-1.14) | | 0.95 (0.83-1.09) | | | | 1.02 (0.89-1.17) | | 0.7839 |  |
| **Model 0:** Stratified for sex, age group and region and adjusted for age (underlying time variable). **Model 1:** Model 0 additionally adjusted for race (4 groups where possible: White, Asian or Asian British, Black or Black British, Mixed race or other, unknown), deprivation (Townsend index quintiles, unknown), qualification (College or university degree/vocational qualification, National examination at ages 17-18,National examination at age 16, unknown), employment (in paid employment, receiving pension, not in paid employment, unknown), smoking (never, former, current <15 cigarettes/day, current >15 cigarettes/ day, current unknown amount of cigarettes/day, unknown), physical activity (<10 excess METs, 10-<50 excess METs, ≥ 50 excess METs, unknown), alcohol intake (none, < 1 g/day, 1-<10 g, 10-<20 g, ≥20 g/day, unknown), menopausal status (pre-, postmenopausal unknown), hormone-replacement therapy (HRT) (never, past, current, unknown) and oral contraceptive pill intake (OCP) (never, past, current, unknown), and parity (nulliparous, 1-2, 3 or more, unknown). **Model 2:** Model 1 additionally adjusted for total fruit and vegetable intake (<3 servings/day, 3-<4 servings/day, 4-<5 servings/day, ≥6 servings/day, unknown), cereal fibre intake (sex-specific quintiles, unknown), oily fish intake (0 times/week,<1 time/week,1 time/week,>2 times/week, unknown) and non-oily fish intake (<1 time/week, 1 time/week, >2 times/week, unknown). **Model 3:** Model 2 additionally adjusted for BMI (sex-specific quintiles, unknown). | | | | | | | | | | | | | | | | | | |  |  |
| Additional file 1: Table 8. Risk of 25 common conditions by unprocessed red meat intake in UK Biobank**.** | | | | | | | | | | | | | | | | | | |  |  |
|  | | |  | | | Unprocessed red meat intake (times/week) | | | | | | | | | |  | |  |  |  |
| Disease | | | Model | | | <1 | | 1 | | 2- 3 | | ≥4 | | | | per 50g/day increase | | p-value |  |  |
| Ischemic heart disease | | | Mean grams/day | | | 6.32 (17.97) | | 32.51 (33.60) | | 44.82 (37.43) | | 54.93 (41.54) | | | |  | |  |  |  |
|  | | | Total n/cases | | | 44617/1021 | | 170451/4794 | | 159659/5219 | | 56502/2100 | | | | 431229/13134 | |  |  |  |
|  | | | HR (95%-CI), Model 0 | | | 1.00 (Ref) | | 1.00 (0.94-1.07) | | 1.06 (0.99-1.14) | | 1.16 (1.08-1.26) | | | | 1.19 (1.10-1.27) | | <0.0001 |  |  |
|  | | | HR (95%-CI), Model 1 | | | 1.00 (Ref) | | 1.08 (1.01-1.16) | | 1.16 (1.08-1.24) | | 1.23 (1.14-1.32) | | | | 1.25 (1.16-1.34) | | <0.0001 |  |  |
|  | | | HR (95%-CI), Model 2 | | | 1.00 (Ref) | | 1.10 (1.03-1.18) | | 1.18 (1.10-1.26) | | 1.24 (1.15-1.34) | | | | 1.26 (1.17-1.35) | | <0.0001 |  |  |
|  | | | HR (95%-CI), Model 3 | | | 1.00 (Ref) | | 1.05 (0.98-1.13) | | 1.10 (1.02-1.18) | | 1.15 (1.06-1.24) | | | | 1.16 (1.08-1.25) | | 0.0001 |  |  |
|  | | |  | | |  | |  | |  | |  | | | |  | |  |  |  |
| Atrial Fibrillation and flutter | | | Mean grams/day | | | 6.35 (18.08) | | 32.52 (33.60) | | 44.82 (37.45) | | 55.00 (41.61) | | | |  | |  |  |  |
|  | | | Total n/cases | | | 44617/411 | | 170451/1798 | | 159659/1848 | | 56502/688 | | | | 431229/4745 | |  |  |  |
|  | | | HR (95%-CI), Model 0 | | | 1.00 (Ref) | | 0.94 (0.85-1.05) | | 0.94 (0.85-1.05) | | 0.97 (0.85-1.09) | | | | 0.97 (0.86-1.09) | | 0.5826 |  |  |
|  | | | HR (95%-CI), Model 1 | | | 1.00 (Ref) | | 0.92 (0.82-1.02) | | 0.90 (0.81-1.01) | | 0.92 (0.81-1.04) | | | | 0.92 (0.82-1.03) | | 0.1495 |  |  |
|  | | | HR (95%-CI), Model 2 | | | 1.00 (Ref) | | 0.94 (0.84-1.05) | | 0.93 (0.83-1.04) | | 0.95 (0.83-1.07) | | | | 0.94 (0.84-1.06) | | 0.3464 |  |  |
|  | | | HR (95%-CI), Model 3 | | | 1.00 (Ref) | | 0.89 (0.80-1.00) | | 0.86 (0.77-0.97) | | 0.86 (0.76-0.98) | | | | 0.86 (0.76-0.97) | | 0.0125 |  |  |
|  | | |  | | |  | |  | |  | |  | | | |  | |  |  |  |
| Cerebrovascular | | | Mean grams/day | | | 6.36 (18.09) | | 32.52 (33.60) | | 44.81 (37.44) | | 54.97 (41.61) | | | |  | |  |  |  |
|  | | | Total n/cases | | | 44617/452 | | 170451/1995 | | 159659/2078 | | 56502/816 | | | | 431229/5341 | |  |  |  |
|  | | | HR (95%-CI), Model 0 | | | 1.00 (Ref) | | 0.99 (0.89-1.10) | | 1.02 (0.92-1.13) | | 1.10 (0.98-1.23) | | | | 1.10 (0.99-1.23) | | 0.0873 |  |  |
|  | | | HR (95%-CI), Model 1 | | | 1.00 (Ref) | | 1.01 (0.91-1.12) | | 1.04 (0.93-1.15) | | 1.08 (0.96-1.21) | | | | 1.08 (0.97-1.21) | | 0.1589 |  |  |
|  | | | HR (95%-CI), Model 2 | | | 1.00 (Ref) | | 1.03 (0.92-1.14) | | 1.05 (0.95-1.17) | | 1.09 (0.96-1.22) | | | | 1.09 (0.98-1.22) | | 0.1211 |  |  |
|  | | | HR (95%-CI), Model 3 | | | 1.00 (Ref) | | 1.01 (0.91-1.12) | | 1.03 (0.92-1.14) | | 1.05 (0.93-1.18) | | | | 1.06 (0.94-1.18) | | 0.3475 |  |  |
|  | | |  | | |  | |  | |  | |  | | | |  | |  |  |  |
| Ischaemic stroke | | | Mean grams/day | | | 6.35 (18.08) | | 32.51 (33.59) | | 44.81 (37.43) | | 54.97 (41.60) | | | |  | |  |  |  |
|  | | | Total n/cases | | | 44617/197 | | 170451/858 | | 159659/907 | | 56502/382 | | | | 431229/2344 | |  |  |  |
|  | | | HR (95%-CI), Model 0 | | | 1.00 (Ref) | | 0.94 (0.80-1.09) | | 0.96 (0.82-1.12) | | 1.12 (0.94-1.33) | | | | 1.12 (0.94-1.32) | | 0.1963 |  |  |
|  | | | HR (95%-CI), Model 1 | | | 1.00 (Ref) | | 0.98 (0.83-1.14) | | 1.00 (0.85-1.17) | | 1.11 (0.93-1.32) | | | | 1.10 (0.93-1.31) | | 0.2446 |  |  |
|  | | | HR (95%-CI), Model 2 | | | 1.00 (Ref) | | 1.00 (0.85-1.17) | | 1.03 (0.87-1.21) | | 1.12 (0.94-1.34) | | | | 1.13 (0.95-1.33) | | 0.1690 |  |  |
|  | | | HR (95%-CI), Model 3 | | | 1.00 (Ref) | | 0.96 (0.82-1.13) | | 0.97 (0.83-1.14) | | 1.05 (0.88-1.26) | | | | 1.05 (0.89-1.25) | | 0.5549 |  |  |
|  | | |  | | |  | |  | |  | |  | | | |  | |  |  |  |
| Haemorrhagic stroke | | | Mean grams/day | | | 6.35 (18.08) | | 32.51 (33.59) | | 44.82 (37.44) | | 54.97 (41.61) | | | |  | |  |  |  |
|  | | | Total n/cases | | | 44617/93 | | 170451/352 | | 159659/360 | | 56502/136 | | | | 431229/941 | |  |  |  |
|  | | | HR (95%-CI), Model 0 | | | 1.00 (Ref) | | 0.92 (0.73-1.16) | | 0.97 (0.77-1.22) | | 1.03 (0.79-1.35) | | | | 1.05 (0.81-1.35) | | 0.7105 |  |  |
|  | | | HR (95%-CI), Model 1 | | | 1.00 (Ref) | | 0.93 (0.74-1.18) | | 0.98 (0.77-1.23) | | 1.02 (0.78-1.33) | | | | 1.04 (0.80-1.34) | | 0.7908 |  |  |
|  | | | HR (95%-CI), Model 2 | | | 1.00 (Ref) | | 0.95 (0.75-1.20) | | 1.00 (0.78-1.26) | | 1.03 (0.79-1.36) | | | | 1.06 (0.81-1.37) | | 0.6775 |  |  |
|  | | | HR (95%-CI), Model 3 | | | 1.00 (Ref) | | 0.96 (0.76-1.22) | | 1.02 (0.80-1.29) | | 1.06 (0.80-1.39) | | | | 1.08 (0.83-1.41) | | 0.5542 |  |  |
|  | | |  | | |  | |  | |  | |  | | | |  | |  |  |  |
| Venous thromboembolism | | | Mean grams/day | | | 6.45 (18.20) | | 32.59 (33.66) | | 45.03 (37.60) | | 55.18 (41.56) | | | |  | |  |  |  |
|  | | | Total n/cases | | | 46743/294 | | 180700/1455 | | 169952/1454 | | 60574/556 | | | | 457969/3759 | |  |  |  |
|  | | | HR (95%-CI), Model 0 | | | 1.00 (Ref) | | 1.14 (1.00-1.29) | | 1.13 (1.00-1.28) | | 1.18 (1.03-1.36) | | | | 1.15 (1.01-1.31) | | 0.0401 |  |  |
|  | | | HR (95%-CI), Model 1 | | | 1.00 (Ref) | | 1.15 (1.01-1.30) | | 1.15 (1.01-1.31) | | 1.20 (1.04-1.38) | | | | 1.17 (1.02-1.33) | | 0.0213 |  |  |
|  | | | HR (95%-CI), Model 2 | | | 1.00 (Ref) | | 1.17 (1.03-1.33) | | 1.17 (1.03-1.34) | | 1.21 (1.05-1.40) | | | | 1.18 (1.03-1.35) | | 0.0158 |  |  |
|  | | | HR (95%-CI), Model 3 | | | 1.00 (Ref) | | 1.09 (0.96-1.24) | | 1.07 (0.94-1.22) | | 1.08 (0.94-1.25) | | | | 1.05 (0.92-1.20) | | 0.4740 |  |  |
|  | | |  | | |  | |  | |  | |  | | | |  | |  |  |  |
| Varicose veins | | | Mean grams/day | | | 6.40 (18.07) | | 32.72 (33.72) | | 45.12 (37.69) | | 55.10 (41.55) | | | |  | |  |  |  |
|  | | | Total n/cases | | | 45558/279 | | 175642/1033 | | 165575/977 | | 59295/347 | | | | 446070/2636 | |  |  |  |
|  | | | HR (95%-CI), Model 0 | | | 1.00 (Ref) | | 0.98 (0.85-1.11) | | 0.98 (0.86-1.12) | | 0.97 (0.82-1.13) | | | | 0.98 (0.84-1.13) | | 0.7421 |  |  |
|  | | | HR (95%-CI), Model 1 | | | 1.00 (Ref) | | 0.98 (0.85-1.12) | | 0.98 (0.86-1.12) | | 0.96 (0.82-1.13) | | | | 0.97 (0.83-1.12) | | 0.6618 |  |  |
|  | | | HR (95%-CI), Model 2 | | | 1.00 (Ref) | | 0.99 (0.87-1.14) | | 1.00 (0.87-1.15) | | 0.98 (0.83-1.15) | | | | 0.99 (0.85-1.15) | | 0.8885 |  |  |
|  | | | HR (95%-CI), Model 3 | | | 1.00 (Ref) | | 0.97 (0.84-1.11) | | 0.96 (0.84-1.11) | | 0.94 (0.80-1.10) | | | | 0.95 (0.81-1.10) | | 0.4674 |  |  |
|  | | |  | | |  | |  | |  | |  | | | |  | |  |  |  |
| Haemorrhoids | | | Mean grams/day | | | 6.42 (18.10) | | 32.60 (33.67) | | 45.02 (37.63) | | 54.98 (41.45) | | | |  | |  |  |  |
|  | | | Total n/cases | | | 45857/824 | | 177080/3262 | | 166452/3088 | | 59286/1131 | | | | 448675/8305 | |  |  |  |
|  | | | HR (95%-CI), Model 0 | | | 1.00 (Ref) | | 1.02 (0.94-1.10) | | 1.02 (0.94-1.10) | | 1.04 (0.95-1.14) | | | | 1.04 (0.96-1.13) | | 0.3729 |  |  |
|  | | | HR (95%-CI), Model 1 | | | 1.00 (Ref) | | 1.04 (0.96-1.12) | | 1.05 (0.97-1.13) | | 1.06 (0.97-1.16) | | | | 1.06 (0.97-1.15) | | 0.2002 |  |  |
|  | | | HR (95%-CI), Model 2 | | | 1.00 (Ref) | | 1.05 (0.97-1.13) | | 1.06 (0.98-1.15) | | 1.07 (0.97-1.17) | | | | 1.07 (0.98-1.16) | | 0.1365 |  |  |
|  | | | HR (95%-CI), Model 3 | | | 1.00 (Ref) | | 1.05 (0.97-1.13) | | 1.06 (0.98-1.15) | | 1.07 (0.98-1.18) | | | | 1.07 (0.98-1.17) | | 0.1310 |  |  |
|  | | |  | | |  | |  | |  | |  | | | |  | |  |  |  |
| Pneumonia | | | Mean grams/day | | | 6.47 (18.19) | | 32.66 (33.65) | | 45.01 (37.62) | | 55.02 (41.57) | | | |  | |  |  |  |
|  | | | Total n/cases | | | 46977/535 | | 181357/2313 | | 170668/2491 | | 60884/1028 | | | | 459886/6367 | |  |  |  |
|  | | | HR (95%-CI), Model 0 | | | 1.00 (Ref) | | 0.98 (0.89-1.08) | | 1.05 (0.96-1.16) | | 1.20 (1.08-1.33) | | | | 1.22 (1.10-1.35) | | 0.0001 |  |  |
|  | | | HR (95%-CI), Model 1 | | | 1.00 (Ref) | | 1.05 (0.96-1.16) | | 1.13 (1.02-1.24) | | 1.21 (1.09-1.34) | | | | 1.23 (1.11-1.37) | | <0.0001 |  |  |
|  | | | HR (95%-CI), Model 2 | | | 1.00 (Ref) | | 1.08 (0.98-1.19) | | 1.16 (1.05-1.28) | | 1.23 (1.10-1.37) | | | | 1.25 (1.13-1.39) | | <0.0001 |  |  |
|  | | | HR (95%-CI), Model 3 | | | 1.00 (Ref) | | 1.07 (0.98-1.18) | | 1.14 (1.04-1.26) | | 1.20 (1.08-1.34) | | | | 1.22 (1.10-1.35) | | 0.0001 |  |  |
|  | | |  | | |  | |  | |  | |  | | | |  | |  |  |  |
| Gastro-oesophageal reflux disease | | | Mean grams/day | | | 6.45 (18.24) | | 32.53 (33.62) | | 45.04 (37.55) | | 55.26 (41.37) | | | |  | |  |  |  |
|  | | | Total n/cases | | | 45228/728 | | 173733/2749 | | 163329/2596 | | 58101/950 | | | | 440391/7023 | |  |  |  |
|  | | | HR (95%-CI), Model 0 | | | 1.00 (Ref) | | 0.96 (0.88-1.04) | | 0.95 (0.88-1.03) | | 0.98 (0.89-1.08) | | | | 0.97 (0.89-1.06) | | 0.5417 |  |  |
|  | | | HR (95%-CI), Model 1 | | | 1.00 (Ref) | | 0.98 (0.90-1.06) | | 0.98 (0.90-1.06) | | 1.01 (0.91-1.11) | | | | 1.00 (0.91-1.09) | | 0.9522 |  |  |
|  | | | HR (95%-CI), Model 2 | | | 1.00 (Ref) | | 1.00 (0.92-1.09) | | 1.00 (0.92-1.09) | | 1.02 (0.92-1.13) | | | | 1.02 (0.93-1.12) | | 0.7319 |  |  |
|  | | | HR (95%-CI), Model 3 | | | 1.00 (Ref) | | 0.98 (0.90-1.07) | | 0.97 (0.89-1.06) | | 0.99 (0.90-1.10) | | | | 0.99 (0.90-1.08) | | 0.7584 |  |  |
|  | | |  | | |  | |  | |  | |  | | | |  | |  |  |  |
| Gastritis and duodenitis | | | Mean grams/day | | | 6.44 (18.12) | | 32.65 (33.70) | | 45.06 (37.61) | | 54.97 (41.49) | | | |  | |  |  |  |
|  | | | Total n/cases | | | 46033/1254 | | 178070/4431 | | 167745/4138 | | 59696/1557 | | | | 451544/11380 | |  |  |  |
|  | | | HR (95%-CI), Model 0 | | | 1.00 (Ref) | | 0.91 (0.85-0.97) | | 0.89 (0.84-0.95) | | 0.93 (0.87-1.01) | | | | 0.92 (0.85-0.98) | | 0.0144 |  |  |
|  | | | HR (95%-CI), Model 1 | | | 1.00 (Ref) | | 0.96 (0.90-1.02) | | 0.95 (0.89-1.02) | | 0.96 (0.89-1.04) | | | | 0.96 (0.89-1.03) | | 0.2408 |  |  |
|  | | | HR (95%-CI), Model 2 | | | 1.00 (Ref) | | 0.98 (0.92-1.05) | | 0.97 (0.91-1.04) | | 0.98 (0.91-1.06) | | | | 0.97 (0.91-1.05) | | 0.4848 |  |  |
|  | | | HR (95%-CI), Model 3 | | | 1.00 (Ref) | | 0.98 (0.91-1.04) | | 0.96 (0.90-1.03) | | 0.97 (0.90-1.05) | | | | 0.96 (0.89-1.03) | | 0.2956 |  |  |
|  | | |  | | |  | |  | |  | |  | | | |  | |  |  |  |
| Inguinal hernia | | | Mean grams/day | | | 6.48 (18.20) | | 32.53 (33.60) | | 44.97 (37.60) | | 55.22 (41.70) | | | |  | |  |  |  |
|  | | | Total n/cases | | | 45731/616 | | 174178/3099 | | 163195/3136 | | 58219/1177 | | | | 441323/8028 | |  |  |  |
|  | | | HR (95%-CI), Model 0 | | | 1.00 (Ref) | | 0.97 (0.89-1.05) | | 0.91 (0.83-0.99) | | 0.89 (0.80-0.98) | | | | 0.86 (0.79-0.94) | | 0.0010 |  |  |
|  | | | HR (95%-CI), Model 1 | | | 1.00 (Ref) | | 0.95 (0.87-1.03) | | 0.90 (0.82-0.98) | | 0.89 (0.81-0.98) | | | | 0.87 (0.80-0.95) | | 0.0031 |  |  |
|  | | | HR (95%-CI), Model 2 | | | 1.00 (Ref) | | 0.95 (0.87-1.04) | | 0.91 (0.83-0.99) | | 0.90 (0.82-1.00) | | | | 0.88 (0.81-0.97) | | 0.0090 |  |  |
|  | | | HR (95%-CI), Model 3 | | | 1.00 (Ref) | | 1.02 (0.93-1.11) | | 1.00 (0.91-1.09) | | 1.02 (0.92-1.13) | | | | 1.00 (0.91-1.10) | | 0.9870 |  |  |
|  | | |  | | |  | |  | |  | |  | | | |  | |  |  |  |
| Noninfective enteritis and colitis | | | Mean grams/day | | | 6.42 (18.14) | | 32.64 (33.69) | | 45.08 (37.67) | | 55.27 (41.59) | | | |  | |  |  |  |
|  | | | Total n/cases | | | 46183/642 | | 179079/2474 | | 168688/2237 | | 60231/888 | | | | 454181/6241 | |  |  |  |
|  | | | HR (95%-CI), Model 0 | | | 1.00 (Ref) | | 0.99 (0.91-1.08) | | 0.95 (0.87-1.04) | | 1.06 (0.96-1.17) | | | | 1.00 (0.91-1.10) | | 0.9729 |  |  |
|  | | | HR (95%-CI), Model 1 | | | 1.00 (Ref) | | 1.02 (0.93-1.11) | | 0.98 (0.89-1.07) | | 1.07 (0.96-1.19) | | | | 1.02 (0.93-1.13) | | 0.6710 |  |  |
|  | | | HR (95%-CI), Model 2 | | | 1.00 (Ref) | | 1.05 (0.96-1.15) | | 1.01 (0.92-1.11) | | 1.09 (0.99-1.22) | | | | 1.05 (0.95-1.16) | | 0.3587 |  |  |
|  | | | HR (95%-CI), Model 3 | | | 1.00 (Ref) | | 1.04 (0.95-1.14) | | 1.00 (0.91-1.09) | | 1.08 (0.97-1.20) | | | | 1.03 (0.93-1.14) | | 0.5718 |  |  |
|  | | |  | | |  | |  | |  | |  | | | |  | |  |  |  |
| Diverticular disease | | | Mean grams/day | | | 6.48 (18.25) | | 32.60 (33.72) | | 44.99 (37.65) | | 55.00 (41.51) | | | |  | |  |  |  |
|  | | | Total n/cases | | | 46748/1040 | | 179901/4853 | | 168996/5016 | | 60198/1920 | | | | 455843/12829 | |  |  |  |
|  | | | HR (95%-CI), Model 0 | | | 1.00 (Ref) | | 1.15 (1.08-1.23) | | 1.22 (1.14-1.31) | | 1.29 (1.20-1.39) | | | | 1.30 (1.21-1.39) | | <0.0001 |  |  |
|  | | | HR (95%-CI), Model 1 | | | 1.00 (Ref) | | 1.12 (1.05-1.20) | | 1.19 (1.11-1.27) | | 1.25 (1.16-1.35) | | | | 1.26 (1.17-1.35) | | <0.0001 |  |  |
|  | | | HR (95%-CI), Model 2 | | | 1.00 (Ref) | | 1.13 (1.05-1.21) | | 1.19 (1.11-1.28) | | 1.25 (1.15-1.35) | | | | 1.25 (1.17-1.35) | | <0.0001 |  |  |
|  | | | HR (95%-CI), Model 3 | | | 1.00 (Ref) | | 1.09 (1.01-1.16) | | 1.13 (1.05-1.21) | | 1.17 (1.08-1.26) | | | | 1.17 (1.09-1.26) | | <0.0001 |  |  |
|  | | |  | | |  | |  | |  | |  | | | |  | |  |  |  |
| Colon polyps | | | Mean grams/day | | | 6.39 (18.08) | | 32.59 (33.69) | | 45.19 (37.72) | | 55.10 (41.40) | | | |  | |  |  |  |
|  | | | Total n/cases | | | 45608/3405 | | 176245/14533 | | 165698/14166 | | 59050/5326 | | | | 446601/37430 | |  |  |  |
|  | | | HR (95%-CI), Model 0 | | | 1.00 (Ref) | | 1.05 (1.01-1.09) | | 1.07 (1.03-1.11) | | 1.14 (1.09-1.19) | | | | 1.13 (1.08-1.17) | | <0.0001 |  |  |
|  | | | HR (95%-CI), Model 1 | | | 1.00 (Ref) | | 1.05 (1.01-1.09) | | 1.06 (1.02-1.11) | | 1.12 (1.07-1.17) | | | | 1.11 (1.07-1.16) | | <0.0001 |  |  |
|  | | | HR (95%-CI), Model 2 | | | 1.00 (Ref) | | 1.06 (1.02-1.10) | | 1.07 (1.03-1.12) | | 1.13 (1.08-1.18) | | | | 1.12 (1.07-1.16) | | <0.0001 |  |  |
|  | | | HR (95%-CI), Model 3 | | | 1.00 (Ref) | | 1.04 (1.00-1.08) | | 1.04 (1.00-1.09) | | 1.09 (1.04-1.14) | | | | 1.08 (1.04-1.13) | | 0.0003 |  |  |
|  | | |  | | |  | |  | |  | |  | | | |  | |  |  |  |
| Gallbladder disease | | | Mean grams/day | | | 6.44 (18.19) | | 32.58 (33.71) | | 45.04 (37.63) | | 55.17 (41.53) | | | |  | |  |  |  |
|  | | | Total n/cases | | | 45931/834 | | 176792/3533 | | 166692/3348 | | 59531/1215 | | | | 448946/8930 | |  |  |  |
|  | | | HR (95%-CI), Model 0 | | | 1.00 (Ref) | | 1.14 (1.06-1.23) | | 1.16 (1.07-1.25) | | 1.18 (1.08-1.29) | | | | 1.17 (1.08-1.26) | | 0.0002 |  |  |
|  | | | HR (95%-CI), Model 1 | | | 1.00 (Ref) | | 1.16 (1.08-1.25) | | 1.20 (1.11-1.30) | | 1.23 (1.12-1.35) | | | | 1.23 (1.13-1.33) | | <0.0001 |  |  |
|  | | | HR (95%-CI), Model 2 | | | 1.00 (Ref) | | 1.19 (1.10-1.29) | | 1.23 (1.14-1.34) | | 1.25 (1.14-1.37) | | | | 1.24 (1.14-1.35) | | <0.0001 |  |  |
|  | | | HR (95%-CI), Model 3 | | | 1.00 (Ref) | | 1.07 (0.99-1.16) | | 1.07 (0.98-1.15) | | 1.05 (0.96-1.15) | | | | 1.04 (0.96-1.14) | | 0.3202 |  |  |
|  | | |  | | |  | |  | |  | |  | | | |  | |  |  |  |
| Osteoarthritis | | | Mean grams/day | | | 6.36 (18.23) | | 32.57 (33.58) | | 44.94 (37.67) | | 55.16 (41.70) | | | |  | |  |  |  |
|  | | | Total n/cases | | | 43086/1562 | | 165138/7052 | | 154659/6992 | | 54780/2489 | | | | 417663/18095 | |  |  |  |
|  | | | HR (95%-CI), Model 0 | | | 1.00 (Ref) | | 1.09 (1.03-1.15) | | 1.12 (1.06-1.18) | | 1.14 (1.07-1.21) | | | | 1.13 (1.07-1.20) | | <0.0001 |  |  |
|  | | | HR (95%-CI), Model 1 | | | 1.00 (Ref) | | 1.08 (1.02-1.14) | | 1.10 (1.04-1.16) | | 1.11 (1.04-1.18) | | | | 1.10 (1.04-1.17) | | 0.0013 |  |  |
|  | | | HR (95%-CI), Model 2 | | | 1.00 (Ref) | | 1.10 (1.04-1.17) | | 1.12 (1.06-1.18) | | 1.13 (1.06-1.20) | | | | 1.12 (1.05-1.19) | | 0.0002 |  |  |
|  | | | HR (95%-CI), Model 3 | | | 1.00 (Ref) | | 1.01 (0.95-1.07) | | 0.98 (0.93-1.04) | | 0.97 (0.91-1.04) | | | | 0.96 (0.90-1.02) | | 0.1818 |  |  |
|  | | |  | | |  | |  | |  | |  | | | |  | |  |  |  |
| Kidney stones | | | Mean grams/day | | | 6.44 (18.16) | | 32.59 (33.67) | | 44.99 (37.59) | | 55.19 (41.56) | | | |  | |  |  |  |
|  | | | Total n/cases | | | 47114/212 | | 181847/983 | | 171146/921 | | 61031/334 | | | | 461138/2450 | |  |  |  |
|  | | | HR (95%-CI), Model 0 | | | 1.00 (Ref) | | 1.10 (0.95-1.28) | | 1.05 (0.91-1.23) | | 1.03 (0.87-1.22) | | | | 1.00 (0.85-1.17) | | 0.9927 |  |  |
|  | | | HR (95%-CI), Model 1 | | | 1.00 (Ref) | | 1.18 (1.02-1.37) | | 1.17 (1.00-1.36) | | 1.13 (0.95-1.35) | | | | 1.11 (0.95-1.30) | | 0.1872 |  |  |
|  | | | HR (95%-CI), Model 2 | | | 1.00 (Ref) | | 1.18 (1.01-1.37) | | 1.16 (0.99-1.35) | | 1.11 (0.93-1.32) | | | | 1.09 (0.93-1.28) | | 0.3088 |  |  |
|  | | | HR (95%-CI), Model 3 | | | 1.00 (Ref) | | 1.11 (0.96-1.30) | | 1.08 (0.92-1.26) | | 1.01 (0.85-1.21) | | | | 1.00 (0.85-1.17) | | 0.9555 |  |  |
|  | | |  | | |  | |  | |  | |  | | | |  | |  |  |  |
| Urinary tract infection | | | Mean grams/day | | | 6.48 (18.26) | | 32.68 (33.72) | | 45.04 (37.67) | | 55.06 (41.47) | | | |  | |  |  |  |
|  | | | Total n/cases | | | 46807/499 | | 181637/1915 | | 171045/2000 | | 61062/802 | | | | 460551/5216 | |  |  |  |
|  | | | HR (95%-CI), Model 0 | | | 1.00 (Ref) | | 0.91 (0.83-1.01) | | 0.96 (0.87-1.06) | | 1.04 (0.93-1.17) | | | | 1.06 (0.95-1.18) | | 0.2946 |  |  |
|  | | | HR (95%-CI), Model 1 | | | 1.00 (Ref) | | 0.98 (0.89-1.08) | | 1.05 (0.95-1.16) | | 1.11 (0.99-1.25) | | | | 1.14 (1.02-1.27) | | 0.0167 |  |  |
|  | | | HR (95%-CI), Model 2 | | | 1.00 (Ref) | | 1.01 (0.92-1.12) | | 1.08 (0.98-1.20) | | 1.14 (1.01-1.28) | | | | 1.17 (1.04-1.30) | | 0.0062 |  |  |
|  | | | HR (95%-CI), Model 3 | | | 1.00 (Ref) | | 0.98 (0.88-1.08) | | 1.03 (0.93-1.14) | | 1.07 (0.95-1.20) | | | | 1.09 (0.98-1.22) | | 0.1135 |  |  |
|  | | |  | | |  | |  | |  | |  | | | |  | |  |  |  |
| Enlarged prostate | | | Mean grams/day | | | 7.56 (20.79) | | 36.26 (36.04) | | 48.60 (39.82) | | 59.58 (43.39) | | | |  | |  |  |  |
|  | | | Total n/cases | | | 15069/246 | | 77800/1312 | | 81640/1446 | | 31333/544 | | | | 205842/3548 | |  |  |  |
|  | | | HR (95%-CI), Model 0 | | | 1.00 (Ref) | | 0.94 (0.82-1.08) | | 0.92 (0.81-1.06) | | 0.88 (0.76-1.03) | | | | 0.89 (0.77-1.02) | | 0.0919 |  |  |
|  | | | HR (95%-CI), Model 1 | | | 1.00 (Ref) | | 1.00 (0.87-1.15) | | 1.02 (0.89-1.17) | | 0.98 (0.84-1.14) | | | | 0.99 (0.86-1.14) | | 0.8821 |  |  |
|  | | | HR (95%-CI), Model 2 | | | 1.00 (Ref) | | 1.02 (0.89-1.17) | | 1.04 (0.90-1.19) | | 1.00 (0.85-1.16) | | | | 1.01 (0.88-1.16) | | 0.9053 |  |  |
|  | | | HR (95%-CI), Model 3 | | | 1.00 (Ref) | | 1.02 (0.89-1.17) | | 1.04 (0.90-1.20) | | 1.00 (0.86-1.17) | | | | 1.01 (0.88-1.17) | | 0.8493 |  |  |
|  | | |  | | |  | |  | |  | |  | | | |  | |  |  |  |
| Female genital prolapse | | | Mean grams/day | | | 5.97 (16.93) | | 29.90 (31.60) | | 41.75 (35.27) | | 50.59 (39.02) | | | |  | |  |  |  |
|  | | | Total n/cases | | | 30661/643 | | 98098/2434 | | 83584/2229 | | 27614/763 | | | | 239957/6069 | |  |  |  |
|  | | | HR (95%-CI), Model 0 | | | 1.00 (Ref) | | 1.12 (1.03-1.22) | | 1.17 (1.07-1.28) | | 1.22 (1.09-1.35) | | | | 1.22 (1.11-1.34) | | 0.0001 |  |  |
|  | | | HR (95%-CI), Model 1 | | | 1.00 (Ref) | | 1.05 (0.96-1.14) | | 1.06 (0.97-1.16) | | 1.12 (1.00-1.24) | | | | 1.10 (1.00-1.22) | | 0.0491 |  |  |
|  | | | HR (95%-CI), Model 2 | | | 1.00 (Ref) | | 1.08 (0.99-1.18) | | 1.10 (1.00-1.20) | | 1.15 (1.03-1.28) | | | | 1.14 (1.03-1.26) | | 0.0105 |  |  |
|  | | | HR (95%-CI), Model 3 | | | 1.00 (Ref) | | 1.04 (0.95-1.14) | | 1.05 (0.96-1.15) | | 1.09 (0.98-1.21) | | | | 1.08 (0.97-1.19) | | 0.1565 |  |  |
|  | | |  | | |  | |  | |  | |  | | | |  | |  |  |  |
| Uterine fibroids | | | Mean grams/day | | | 5.72 (16.69) | | 29.63 (31.59) | | 41.39 (34.90) | | 50.56 (39.04) | | | |  | |  |  |  |
|  | | | Total n/cases | | | 23965/1232 | | 74571/3824 | | 62912/3135 | | 20555/1075 | | | | 182003/9266 | |  |  |  |
|  | | | HR (95%-CI), Model 0 | | | 1.00 (Ref) | | 1.05 (0.98-1.12) | | 1.04 (0.97-1.11) | | 1.09 (1.01-1.19) | | | | 1.07 (0.99-1.15) | | 0.0775 |  |  |
|  | | | HR (95%-CI), Model 1 | | | 1.00 (Ref) | | 1.06 (1.00-1.14) | | 1.07 (1.00-1.14) | | 1.10 (1.01-1.20) | | | | 1.09 (1.01-1.17) | | 0.0222 |  |  |
|  | | | HR (95%-CI), Model 2 | | | 1.00 (Ref) | | 1.08 (1.01-1.15) | | 1.08 (1.01-1.16) | | 1.11 (1.02-1.21) | | | | 1.10 (1.02-1.19) | | 0.0130 |  |  |
|  | | | HR (95%-CI), Model 3 | | | 1.00 (Ref) | | 1.03 (0.96-1.10) | | 1.02 (0.95-1.09) | | 1.03 (0.95-1.13) | | | | 1.02 (0.95-1.10) | | 0.5924 |  |  |
|  | | |  | | |  | |  | |  | |  | | | |  | |  |  |  |
| Iron deficiency anaemia | | | Mean grams/day | | | 6.46 (18.20) | | 32.64 (33.70) | | 45.10 (37.65) | | 55.20 (41.57) | | | |  | |  |  |  |
|  | | | Total n/cases | | | 46762/635 | | 182479/1878 | | 172025/1598 | | 61408/617 | | | | 462674/4728 | |  |  |  |
|  | | | HR (95%-CI), Model 0 | | | 1.00 (Ref) | | 0.73 (0.67-0.80) | | 0.65 (0.60-0.72) | | 0.72 (0.65-0.81) | | | | 0.66 (0.59-0.73) | | <0.0001 |  |  |
|  | | | HR (95%-CI), Model 1 | | | 1.00 (Ref) | | 0.84 (0.77-0.92) | | 0.78 (0.71-0.86) | | 0.85 (0.76-0.95) | | | | 0.80 (0.72-0.89) | | <0.0001 |  |  |
|  | | | HR (95%-CI), Model 2 | | | 1.00 (Ref) | | 0.88 (0.80-0.97) | | 0.83 (0.75-0.91) | | 0.88 (0.79-0.99) | | | | 0.84 (0.75-0.94) | | 0.0015 |  |  |
|  | | | HR (95%-CI), Model 3 | | | 1.00 (Ref) | | 0.86 (0.79-0.95) | | 0.80 (0.72-0.88) | | 0.85 (0.75-0.95) | | | | 0.80 (0.72-0.90) | | 0.0001 |  |  |
|  | | |  | | |  | |  | |  | |  | | | |  | |  |  |  |
| Diabetes | | | Mean grams/day | | | 6.42 (18.18) | | 32.40 (33.57) | | 44.77 (37.54) | | 54.90 (41.23) | | | |  | |  |  |  |
|  | | | Total n/cases | | | 45582/803 | | 175653/3453 | | 164017/3810 | | 58003/1529 | | | | 443255/9595 | |  |  |  |
|  | | | HR (95%-CI), Model 0 | | | 1.00 (Ref) | | 0.99 (0.91-1.07) | | 1.11 (1.03-1.20) | | 1.27 (1.16-1.38) | | | | 1.33 (1.23-1.45) | | <0.0001 |  |  |
|  | | | HR (95%-CI), Model 1 | | | 1.00 (Ref) | | 1.18 (1.09-1.27) | | 1.37 (1.26-1.48) | | 1.45 (1.33-1.58) | | | | 1.53 (1.41-1.66) | | <0.0001 |  |  |
|  | | | HR (95%-CI), Model 2 | | | 1.00 (Ref) | | 1.21 (1.12-1.31) | | 1.39 (1.29-1.51) | | 1.47 (1.34-1.60) | | | | 1.54 (1.41-1.67) | | <0.0001 |  |  |
|  | | | HR (95%-CI), Model 3 | | | 1.00 (Ref) | | 1.05 (0.97-1.14) | | 1.15 (1.06-1.24) | | 1.16 (1.06-1.27) | | | | 1.21 (1.11-1.32) | | <0.0001 |  |  |
|  | | |  | | |  | |  | |  | |  | | | |  | |  |  |  |
| Carpal tunnel syndrome | | | Mean grams/day | | | 6.42 (18.07) | | 32.63 (33.66) | | 45.01 (37.59) | | 55.28 (41.61) | | | |  | |  |  |  |
|  | | | Total n/cases | | | 46826/485 | | 180904/1967 | | 170480/1900 | | 60851/703 | | | | 459061/5055 | |  |  |  |
|  | | | HR (95%-CI), Model 0 | | | 1.00 (Ref) | | 1.08 (0.97-1.19) | | 1.12 (1.01-1.23) | | 1.19 (1.06-1.33) | | | | 1.18 (1.06-1.32) | | 0.0026 |  |  |
|  | | | HR (95%-CI), Model 1 | | | 1.00 (Ref) | | 1.10 (1.00-1.22) | | 1.15 (1.04-1.27) | | 1.19 (1.06-1.34) | | | | 1.20 (1.07-1.33) | | 0.0013 |  |  |
|  | | | HR (95%-CI), Model 2 | | | 1.00 (Ref) | | 1.12 (1.01-1.24) | | 1.17 (1.06-1.30) | | 1.21 (1.07-1.36) | | | | 1.21 (1.09-1.35) | | 0.0007 |  |  |
|  | | | HR (95%-CI), Model 3 | | | 1.00 (Ref) | | 1.03 (0.93-1.14) | | 1.04 (0.94-1.16) | | 1.05 (0.93-1.19) | | | | 1.05 (0.94-1.18) | | 0.3574 |  |  |
|  | | |  | | |  | |  | |  | |  | | | |  | |  |  |  |
| Cataracts | | | Mean grams/day | | | 6.47 (18.29) | | 32.39 (33.57) | | 44.74 (37.54) | | 54.76 (41.18) | | | |  | |  |  |  |
|  | | | Total n/cases | | | 43877/1566 | | 168966/6176 | | 157871/6207 | | 55749/2192 | | | | 426463/16141 | |  |  |  |
|  | | | HR (95%-CI), Model 0 | | | 1.00 (Ref) | | 0.90 (0.85-0.95) | | 0.90 (0.85-0.95) | | 0.88 (0.83-0.94) | | | | 0.90 (0.84-0.95) | | 0.0006 |  |  |
|  | | | HR (95%-CI), Model 1 | | | 1.00 (Ref) | | 0.96 (0.91-1.02) | | 0.97 (0.92-1.03) | | 0.93 (0.87-0.99) | | | | 0.94 (0.89-1.00) | | 0.0706 |  |  |
|  | | | HR (95%-CI), Model 2 | | | 1.00 (Ref) | | 0.97 (0.92-1.03) | | 0.98 (0.93-1.04) | | 0.93 (0.87-1.00) | | | | 0.95 (0.89-1.01) | | 0.1291 |  |  |
|  | | | HR (95%-CI), Model 3 | | | 1.00 (Ref) | | 0.97 (0.91-1.02) | | 0.97 (0.91-1.03) | | 0.92 (0.86-0.98) | | | | 0.93 (0.88-1.00) | | 0.0366 |  |  |
|  | | |  | | |  | |  | |  | |  | | | |  | |  |  |  |
| Cellulitis | | | Mean grams/day | | | 6.47 (18.21) | | 32.64 (33.69) | | 45.02 (37.63) | | 55.02 (41.44) | | | |  | |  |  |  |
|  | | | Total n/cases | | | 47352/336 | | 183083/1252 | | 172273/1327 | | 61483/499 | | | | 464191/3414 | |  |  |  |
|  | | | HR (95%-CI), Model 0 | | | 1.00 (Ref) | | 0.89 (0.79-1.01) | | 0.96 (0.85-1.09) | | 0.99 (0.86-1.13) | | | | 1.03 (0.90-1.18) | | 0.6607 |  |  |
|  | | | HR (95%-CI), Model 1 | | | 1.00 (Ref) | | 0.95 (0.84-1.07) | | 1.03 (0.91-1.16) | | 1.03 (0.89-1.18) | | | | 1.08 (0.94-1.23) | | 0.2679 |  |  |
|  | | | HR (95%-CI), Model 2 | | | 1.00 (Ref) | | 0.97 (0.86-1.10) | | 1.06 (0.93-1.20) | | 1.05 (0.91-1.21) | | | | 1.10 (0.96-1.26) | | 0.1598 |  |  |
|  | | | HR (95%-CI), Model 3 | | | 1.00 (Ref) | | 0.88 (0.78-1.00) | | 0.92 (0.82-1.05) | | 0.89 (0.77-1.03) | | | | 0.93 (0.81-1.07) | | 0.3005 |  |  |
| **Model 0:** Stratified for sex, age group and region and adjusted for age (underlying time variable). **Model 1:** Model 0 additionally adjusted for race (4 groups where possible: White, Asian or Asian British, Black or Black British, Mixed race or other, unknown), deprivation (Townsend index quintiles, unknown), qualification (College or university degree/vocational qualification, National examination at ages 17-18,National examination at age 16, unknown), employment (in paid employment, receiving pension, not in paid employment, unknown), smoking (never, former, current <15 cigarettes/day, current >15 cigarettes/ day, current unknown amount of cigarettes/day, unknown), physical activity (<10 excess METs, 10-<50 excess METs, ≥ 50 excess METs, unknown), alcohol intake (none, < 1 g/day, 1-<10 g, 10-<20 g, ≥20 g/day, unknown), menopausal status (pre-, postmenopausal unknown), hormone-replacement therapy (HRT) (never, past, current, unknown) and oral contraceptive pill intake (OCP) (never, past, current, unknown), and parity (nulliparous, 1-2, 3 or more, unknown). **Model 2:** Model 1 additionally adjusted for total fruit and vegetable intake (<3 servings/day, 3-<4 servings/day, 4-<5 servings/day, ≥6 servings/day, unknown), cereal fibre intake (sex-specific quintiles, unknown), oily fish intake (0 times/week,<1 time/week,1 time/week,>2 times/week, unknown) and non-oily fish intake (<1 time/week, 1 time/week, >2 times/week, unknown). **Model 3:** Model 2 additionally adjusted for BMI (sex-specific quintiles, unknown). | | | | | | | | | | | | | | | | | | |  |  |
| Additional file 1: Table 9. Risk of 25 common conditions by processed meat intake in UK Biobank. | | | | | | | | | | | | | | | | | | |  |  |
|  | | |  | | | Processed meat intake (times/week) | | | | | | | | | |  | |  |  |  |
| Disease | | | Model | | | 0 | | <1 | | 1 | | ≥2 | | | | per 20g/day increase | | p-value |  |  |
| Ischemic heart disease | | | Mean grams/day | | | 3.94 (11.36) | | 15.54 (20.07) | | 21.23 (23.52) | | 28.89 (28.38) | | | |  | |  |  |  |
|  | | | Total n/cases | | | 41076/878 | | 133163/3429 | | 126995/4010 | | 133954/4992 | | | | 435188/13309 | |  |  |  |
|  | | | HR (95%-CI), Model 0 | | | 1.00 (Ref) | | 1.05 (0.98-1.13) | | 1.12 (1.04-1.21) | | 1.18 (1.10-1.27) | | | | 1.16 (1.11-1.22) | | <0.0001 |  |  |
|  | | | HR (95%-CI), Model 1 | | | 1.00 (Ref) | | 1.14 (1.06-1.23) | | 1.21 (1.13-1.31) | | 1.24 (1.15-1.34) | | | | 1.17 (1.11-1.23) | | <0.0001 |  |  |
|  | | | HR (95%-CI), Model 2 | | | 1.00 (Ref) | | 1.16 (1.07-1.25) | | 1.23 (1.14-1.33) | | 1.25 (1.16-1.35) | | | | 1.16 (1.10-1.22) | | <0.0001 |  |  |
|  | | | HR (95%-CI), Model 3 | | | 1.00 (Ref) | | 1.10 (1.02-1.19) | | 1.14 (1.06-1.23) | | 1.15 (1.06-1.24) | | | | 1.09 (1.04-1.15) | | 0.0006 |  |  |
|  | | |  | | |  | |  | |  | |  | | | |  | |  |  |  |
| Atrial Fibrillation and flutter | | | Mean grams/day | | | 3.94 (11.36) | | 15.54 (20.07) | | 21.24 (23.54) | | 28.88 (28.37) | | | |  | |  |  |  |
|  | | | Total n/cases | | | 41076/366 | | 133163/1342 | | 126995/1429 | | 133954/1658 | | | | 435188/4795 | |  |  |  |
|  | | | HR (95%-CI), Model 0 | | | 1.00 (Ref) | | 1.00 (0.89-1.12) | | 1.01 (0.90-1.14) | | 1.04 (0.93-1.17) | | | | 1.04 (0.96-1.13) | | 0.3266 |  |  |
|  | | | HR (95%-CI), Model 1 | | | 1.00 (Ref) | | 0.97 (0.86-1.09) | | 0.97 (0.86-1.09) | | 0.98 (0.87-1.10) | | | | 1.00 (0.92-1.08) | | 0.9140 |  |  |
|  | | | HR (95%-CI), Model 2 | | | 1.00 (Ref) | | 1.01 (0.89-1.13) | | 1.02 (0.90-1.15) | | 1.03 (0.92-1.16) | | | | 1.03 (0.95-1.12) | | 0.4929 |  |  |
|  | | | HR (95%-CI), Model 3 | | | 1.00 (Ref) | | 0.96 (0.85-1.08) | | 0.95 (0.84-1.07) | | 0.94 (0.83-1.06) | | | | 0.96 (0.88-1.04) | | 0.3377 |  |  |
|  | | |  | | |  | |  | |  | |  | | | |  | |  |  |  |
| Cerebrovascular | | | Mean grams/day | | | 3.95 (11.36) | | 15.54 (20.06) | | 21.24 (23.53) | | 28.87 (28.34) | | | |  | |  |  |  |
|  | | | Total n/cases | | | 41076/389 | | 133163/1543 | | 126995/1591 | | 133954/1895 | | | | 435188/5418 | |  |  |  |
|  | | | HR (95%-CI), Model 0 | | | 1.00 (Ref) | | 1.10 (0.98-1.23) | | 1.12 (1.00-1.25) | | 1.23 (1.10-1.37) | | | | 1.18 (1.09-1.28) | | <0.0001 |  |  |
|  | | | HR (95%-CI), Model 1 | | | 1.00 (Ref) | | 1.14 (1.01-1.27) | | 1.14 (1.02-1.28) | | 1.20 (1.07-1.34) | | | | 1.13 (1.04-1.22) | | 0.0022 |  |  |
|  | | | HR (95%-CI), Model 2 | | | 1.00 (Ref) | | 1.15 (1.03-1.29) | | 1.16 (1.03-1.30) | | 1.21 (1.08-1.36) | | | | 1.13 (1.04-1.22) | | 0.0026 |  |  |
|  | | | HR (95%-CI), Model 3 | | | 1.00 (Ref) | | 1.14 (1.01-1.27) | | 1.13 (1.01-1.27) | | 1.17 (1.05-1.32) | | | | 1.10 (1.02-1.19) | | 0.0163 |  |  |
|  | | |  | | |  | |  | |  | |  | | | |  | |  |  |  |
| Ischaemic stroke | | | Mean grams/day | | | 3.95 (11.36) | | 15.55 (20.07) | | 21.23 (23.53) | | 28.88 (28.35) | | | |  | |  |  |  |
|  | | | Total n/cases | | | 41076/158 | | 133163/626 | | 126995/709 | | 133954/890 | | | | 435188/2383 | |  |  |  |
|  | | | HR (95%-CI), Model 0 | | | 1.00 (Ref) | | 1.07 (0.90-1.28) | | 1.16 (0.98-1.38) | | 1.31 (1.10-1.56) | | | | 1.29 (1.14-1.45) | | <0.0001 |  |  |
|  | | | HR (95%-CI), Model 1 | | | 1.00 (Ref) | | 1.13 (0.95-1.35) | | 1.21 (1.01-1.44) | | 1.29 (1.09-1.54) | | | | 1.23 (1.09-1.38) | | 0.0009 |  |  |
|  | | | HR (95%-CI), Model 2 | | | 1.00 (Ref) | | 1.16 (0.97-1.39) | | 1.25 (1.04-1.49) | | 1.32 (1.10-1.57) | | | | 1.23 (1.09-1.39) | | 0.0009 |  |  |
|  | | | HR (95%-CI), Model 3 | | | 1.00 (Ref) | | 1.12 (0.94-1.34) | | 1.18 (0.99-1.42) | | 1.24 (1.03-1.48) | | | | 1.17 (1.04-1.32) | | 0.0107 |  |  |
|  | | |  | | |  | |  | |  | |  | | | |  | |  |  |  |
| Haemorrhagic stroke | | | Mean grams/day | | | 3.94 (11.35) | | 15.54 (20.06) | | 21.23 (23.53) | | 28.87 (28.35) | | | |  | |  |  |  |
|  | | | Total n/cases | | | 41076/86 | | 133163/298 | | 126995/267 | | 133954/304 | | | | 435188/955 | |  |  |  |
|  | | | HR (95%-CI), Model 0 | | | 1.00 (Ref) | | 1.02 (0.80-1.29) | | 0.96 (0.75-1.22) | | 1.07 (0.84-1.36) | | | | 1.05 (0.88-1.26) | | 0.5779 |  |  |
|  | | | HR (95%-CI), Model 1 | | | 1.00 (Ref) | | 1.04 (0.82-1.33) | | 0.97 (0.76-1.24) | | 1.05 (0.82-1.35) | | | | 1.02 (0.85-1.23) | | 0.7960 |  |  |
|  | | | HR (95%-CI), Model 2 | | | 1.00 (Ref) | | 1.06 (0.83-1.36) | | 0.98 (0.76-1.26) | | 1.05 (0.82-1.36) | | | | 1.02 (0.85-1.22) | | 0.8531 |  |  |
|  | | | HR (95%-CI), Model 3 | | | 1.00 (Ref) | | 1.08 (0.84-1.38) | | 1.01 (0.78-1.30) | | 1.08 (0.84-1.40) | | | | 1.04 (0.86-1.25) | | 0.6836 |  |  |
|  | | |  | | |  | |  | |  | |  | | | |  | |  |  |  |
| Venous thromboembolism | | | Mean grams/day | | | 4.10 (11.61) | | 15.55 (20.08) | | 21.30 (23.58) | | 29.05 (28.47) | | | |  | |  |  |  |
|  | | | Total n/cases | | | 43110/260 | | 140376/1074 | | 134963/1129 | | 143900/1355 | | | | 462349/3818 | |  |  |  |
|  | | | HR (95%-CI), Model 0 | | | 1.00 (Ref) | | 1.17 (1.02-1.34) | | 1.21 (1.06-1.39) | | 1.32 (1.15-1.51) | | | | 1.23 (1.12-1.35) | | <0.0001 |  |  |
|  | | | HR (95%-CI), Model 1 | | | 1.00 (Ref) | | 1.18 (1.03-1.35) | | 1.21 (1.05-1.39) | | 1.29 (1.13-1.48) | | | | 1.19 (1.09-1.31) | | 0.0002 |  |  |
|  | | | HR (95%-CI), Model 2 | | | 1.00 (Ref) | | 1.20 (1.05-1.38) | | 1.23 (1.07-1.42) | | 1.31 (1.14-1.50) | | | | 1.19 (1.09-1.31) | | 0.0003 |  |  |
|  | | | HR (95%-CI), Model 3 | | | 1.00 (Ref) | | 1.12 (0.98-1.29) | | 1.12 (0.97-1.29) | | 1.16 (1.01-1.33) | | | | 1.10 (1.00-1.20) | | 0.0611 |  |  |
|  | | |  | | |  | |  | |  | |  | | | |  | |  |  |  |
| Varicose veins | | | Mean grams/day | | | 4.09 (11.65) | | 15.56 (20.09) | | 21.37 (23.67) | | 29.20 (28.49) | | | |  | |  |  |  |
|  | | | Total n/cases | | | 41923/267 | | 135968/811 | | 131318/797 | | 141156/784 | | | | 450365/2659 | |  |  |  |
|  | | | HR (95%-CI), Model 0 | | | 1.00 (Ref) | | 0.93 (0.81-1.07) | | 0.98 (0.85-1.12) | | 0.92 (0.80-1.06) | | | | 0.96 (0.86-1.06) | | 0.4112 |  |  |
|  | | | HR (95%-CI), Model 1 | | | 1.00 (Ref) | | 0.94 (0.81-1.08) | | 0.97 (0.84-1.11) | | 0.91 (0.79-1.05) | | | | 0.94 (0.85-1.05) | | 0.2613 |  |  |
|  | | | HR (95%-CI), Model 2 | | | 1.00 (Ref) | | 0.96 (0.83-1.10) | | 1.00 (0.86-1.15) | | 0.95 (0.82-1.10) | | | | 0.97 (0.87-1.08) | | 0.5975 |  |  |
|  | | | HR (95%-CI), Model 3 | | | 1.00 (Ref) | | 0.93 (0.81-1.07) | | 0.96 (0.83-1.11) | | 0.90 (0.78-1.05) | | | | 0.94 (0.84-1.04) | | 0.2405 |  |  |
|  | | |  | | |  | |  | |  | |  | | | |  | |  |  |  |
| Haemorrhoids | | | Mean grams/day | | | 4.10 (11.61) | | 15.58 (20.07) | | 21.32 (23.63) | | 29.01 (28.50) | | | |  | |  |  |  |
|  | | | Total n/cases | | | 42305/760 | | 137568/2612 | | 132420/2359 | | 140682/2677 | | | | 452975/8408 | |  |  |  |
|  | | | HR (95%-CI), Model 0 | | | 1.00 (Ref) | | 1.05 (0.96-1.13) | | 0.96 (0.89-1.05) | | 1.00 (0.93-1.09) | | | | 0.98 (0.92-1.04) | | 0.4802 |  |  |
|  | | | HR (95%-CI), Model 1 | | | 1.00 (Ref) | | 1.07 (0.98-1.16) | | 0.99 (0.91-1.07) | | 1.04 (0.95-1.13) | | | | 1.00 (0.94-1.06) | | 0.9903 |  |  |
|  | | | HR (95%-CI), Model 2 | | | 1.00 (Ref) | | 1.08 (0.99-1.17) | | 1.00 (0.92-1.09) | | 1.04 (0.96-1.14) | | | | 1.00 (0.94-1.07) | | 0.8915 |  |  |
|  | | | HR (95%-CI), Model 3 | | | 1.00 (Ref) | | 1.08 (0.99-1.17) | | 1.00 (0.91-1.09) | | 1.04 (0.96-1.14) | | | | 1.00 (0.94-1.07) | | 0.8935 |  |  |
|  | | |  | | |  | |  | |  | |  | | | |  | |  |  |  |
| Pneumonia | | | Mean grams/day | | | 4.09 (11.56) | | 15.56 (20.06) | | 21.27 (23.57) | | 29.06 (28.50) | | | |  | |  |  |  |
|  | | | Total n/cases | | | 43304/480 | | 141138/1651 | | 135522/1932 | | 144325/2440 | | | | 464289/6503 | |  |  |  |
|  | | | HR (95%-CI), Model 0 | | | 1.00 (Ref) | | 0.97 (0.88-1.08) | | 1.13 (1.02-1.25) | | 1.32 (1.20-1.46) | | | | 1.37 (1.28-1.47) | | <0.0001 |  |  |
|  | | | HR (95%-CI), Model 1 | | | 1.00 (Ref) | | 1.04 (0.94-1.16) | | 1.17 (1.06-1.30) | | 1.27 (1.15-1.41) | | | | 1.26 (1.18-1.36) | | <0.0001 |  |  |
|  | | | HR (95%-CI), Model 2 | | | 1.00 (Ref) | | 1.07 (0.97-1.19) | | 1.20 (1.08-1.33) | | 1.29 (1.16-1.43) | | | | 1.26 (1.17-1.35) | | <0.0001 |  |  |
|  | | | HR (95%-CI), Model 3 | | | 1.00 (Ref) | | 1.07 (0.96-1.19) | | 1.19 (1.07-1.32) | | 1.26 (1.14-1.40) | | | | 1.23 (1.15-1.32) | | <0.0001 |  |  |
|  | | |  | | |  | |  | |  | |  | | | |  | |  |  |  |
| Gastro-oesophageal reflux disease | | | Mean grams/day | | | 4.06 (11.64) | | 15.53 (20.07) | | 21.28 (23.63) | | 28.93 (28.33) | | | |  | |  |  |  |
|  | | | Total n/cases | | | 41698/664 | | 135257/2222 | | 129683/2090 | | 137919/2137 | | | | 444557/7113 | |  |  |  |
|  | | | HR (95%-CI), Model 0 | | | 1.00 (Ref) | | 1.01 (0.92-1.10) | | 1.00 (0.92-1.10) | | 0.99 (0.91-1.08) | | | | 0.99 (0.93-1.06) | | 0.7367 |  |  |
|  | | | HR (95%-CI), Model 1 | | | 1.00 (Ref) | | 1.02 (0.94-1.12) | | 1.02 (0.93-1.11) | | 0.99 (0.91-1.09) | | | | 0.98 (0.92-1.05) | | 0.6377 |  |  |
|  | | | HR (95%-CI), Model 2 | | | 1.00 (Ref) | | 1.04 (0.95-1.14) | | 1.03 (0.94-1.13) | | 1.00 (0.91-1.10) | | | | 0.98 (0.92-1.05) | | 0.6273 |  |  |
|  | | | HR (95%-CI), Model 3 | | | 1.00 (Ref) | | 1.02 (0.93-1.12) | | 1.01 (0.92-1.10) | | 0.97 (0.88-1.07) | | | | 0.96 (0.90-1.03) | | 0.2515 |  |  |
|  | | |  | | |  | |  | |  | |  | | | |  | |  |  |  |
| Gastritis and duodenitis | | | Mean grams/day | | | 4.08 (11.62) | | 15.55 (20.09) | | 21.27 (23.63) | | 29.04 (28.49) | | | |  | |  |  |  |
|  | | | Total n/cases | | | 42458/1133 | | 138632/3639 | | 133120/3202 | | 141608/3565 | | | | 455818/11539 | |  |  |  |
|  | | | HR (95%-CI), Model 0 | | | 1.00 (Ref) | | 0.97 (0.91-1.04) | | 0.93 (0.86-0.99) | | 1.02 (0.95-1.09) | | | | 1.02 (0.97-1.07) | | 0.4537 |  |  |
|  | | | HR (95%-CI), Model 1 | | | 1.00 (Ref) | | 1.03 (0.97-1.11) | | 0.98 (0.91-1.05) | | 1.05 (0.98-1.13) | | | | 1.03 (0.98-1.08) | | 0.3064 |  |  |
|  | | | HR (95%-CI), Model 2 | | | 1.00 (Ref) | | 1.06 (0.99-1.13) | | 1.00 (0.93-1.07) | | 1.06 (0.99-1.14) | | | | 1.03 (0.98-1.09) | | 0.2670 |  |  |
|  | | | HR (95%-CI), Model 3 | | | 1.00 (Ref) | | 1.05 (0.98-1.13) | | 0.99 (0.92-1.06) | | 1.05 (0.98-1.13) | | | | 1.02 (0.97-1.07) | | 0.4706 |  |  |
|  | | |  | | |  | |  | |  | |  | | | |  | |  |  |  |
| Inguinal hernia | | | Mean grams/day | | | 4.10 (11.64) | | 15.60 (20.12) | | 21.33 (23.52) | | 28.96 (28.43) | | | |  | |  |  |  |
|  | | | Total n/cases | | | 42341/497 | | 136864/1921 | | 129868/2495 | | 136518/3195 | | | | 445591/8108 | |  |  |  |
|  | | | HR (95%-CI), Model 0 | | | 1.00 (Ref) | | 0.96 (0.87-1.06) | | 0.96 (0.87-1.06) | | 0.90 (0.82-0.99) | | | | 0.91 (0.85-0.97) | | 0.0035 |  |  |
|  | | | HR (95%-CI), Model 1 | | | 1.00 (Ref) | | 0.94 (0.85-1.04) | | 0.94 (0.86-1.04) | | 0.90 (0.81-0.99) | | | | 0.92 (0.86-0.98) | | 0.0105 |  |  |
|  | | | HR (95%-CI), Model 2 | | | 1.00 (Ref) | | 0.95 (0.85-1.05) | | 0.95 (0.86-1.06) | | 0.91 (0.82-1.00) | | | | 0.93 (0.87-0.99) | | 0.0326 |  |  |
|  | | | HR (95%-CI), Model 3 | | | 1.00 (Ref) | | 1.02 (0.92-1.13) | | 1.05 (0.95-1.17) | | 1.03 (0.93-1.13) | | | | 1.01 (0.95-1.08) | | 0.7005 |  |  |
|  | | |  | | |  | |  | |  | |  | | | |  | |  |  |  |
| Noninfective enteritis and colitis | | | Mean grams/day | | | 4.08 (11.66) | | 15.55 (20.07) | | 21.25 (23.63) | | 29.07 (28.50) | | | |  | |  |  |  |
|  | | | Total n/cases | | | 42584/615 | | 139224/1940 | | 134030/1819 | | 142710/1957 | | | | 458548/6331 | |  |  |  |
|  | | | HR (95%-CI), Model 0 | | | 1.00 (Ref) | | 0.94 (0.86-1.03) | | 0.96 (0.87-1.05) | | 1.02 (0.93-1.12) | | | | 1.05 (0.98-1.12) | | 0.2019 |  |  |
|  | | | HR (95%-CI), Model 1 | | | 1.00 (Ref) | | 0.97 (0.88-1.06) | | 0.97 (0.88-1.06) | | 1.01 (0.92-1.11) | | | | 1.02 (0.96-1.10) | | 0.4947 |  |  |
|  | | | HR (95%-CI), Model 2 | | | 1.00 (Ref) | | 0.99 (0.91-1.09) | | 1.00 (0.91-1.10) | | 1.03 (0.94-1.13) | | | | 1.03 (0.96-1.11) | | 0.3764 |  |  |
|  | | | HR (95%-CI), Model 3 | | | 1.00 (Ref) | | 0.99 (0.90-1.08) | | 0.98 (0.89-1.08) | | 1.01 (0.92-1.11) | | | | 1.02 (0.95-1.09) | | 0.6547 |  |  |
|  | | |  | | |  | |  | |  | |  | | | |  | |  |  |  |
| Diverticular disease | | | Mean grams/day | | | 4.08 (11.64) | | 15.55 (20.08) | | 21.27 (23.61) | | 29.08 (28.52) | | | |  | |  |  |  |
|  | | | Total n/cases | | | 43091/986 | | 139689/3917 | | 134286/3935 | | 143145/4135 | | | | 460211/12973 | |  |  |  |
|  | | | HR (95%-CI), Model 0 | | | 1.00 (Ref) | | 1.18 (1.10-1.26) | | 1.28 (1.19-1.37) | | 1.33 (1.24-1.42) | | | | 1.23 (1.17-1.29) | | <0.0001 |  |  |
|  | | | HR (95%-CI), Model 1 | | | 1.00 (Ref) | | 1.15 (1.07-1.23) | | 1.23 (1.14-1.32) | | 1.25 (1.17-1.35) | | | | 1.18 (1.12-1.24) | | <0.0001 |  |  |
|  | | | HR (95%-CI), Model 2 | | | 1.00 (Ref) | | 1.16 (1.08-1.24) | | 1.23 (1.15-1.33) | | 1.25 (1.16-1.34) | | | | 1.17 (1.11-1.23) | | <0.0001 |  |  |
|  | | | HR (95%-CI), Model 3 | | | 1.00 (Ref) | | 1.11 (1.03-1.19) | | 1.17 (1.09-1.26) | | 1.17 (1.09-1.26) | | | | 1.11 (1.06-1.17) | | <0.0001 |  |  |
|  | | |  | | |  | |  | |  | |  | | | |  | |  |  |  |
| Colon polyps | | | Mean grams/day | | | 4.11 (11.71) | | 15.56 (20.12) | | 21.30 (23.69) | | 29.06 (28.42) | | | |  | |  |  |  |
|  | | | Total n/cases | | | 42006/3171 | | 136949/11092 | | 131907/11177 | | 139998/12439 | | | | 450860/37879 | |  |  |  |
|  | | | HR (95%-CI), Model 0 | | | 1.00 (Ref) | | 1.03 (0.99-1.07) | | 1.08 (1.04-1.13) | | 1.15 (1.11-1.20) | | | | 1.14 (1.11-1.17) | | <0.0001 |  |  |
|  | | | HR (95%-CI), Model 1 | | | 1.00 (Ref) | | 1.03 (0.99-1.07) | | 1.07 (1.03-1.12) | | 1.12 (1.08-1.17) | | | | 1.11 (1.08-1.14) | | <0.0001 |  |  |
|  | | | HR (95%-CI), Model 2 | | | 1.00 (Ref) | | 1.04 (1.00-1.08) | | 1.09 (1.04-1.13) | | 1.13 (1.08-1.17) | | | | 1.11 (1.08-1.14) | | <0.0001 |  |  |
|  | | | HR (95%-CI), Model 3 | | | 1.00 (Ref) | | 1.02 (0.98-1.06) | | 1.06 (1.01-1.10) | | 1.09 (1.04-1.13) | | | | 1.08 (1.05-1.11) | | <0.0001 |  |  |
|  | | |  | | |  | |  | |  | |  | | | |  | |  |  |  |
| Gallbladder disease | | | Mean grams/day | | | 4.08 (11.66) | | 15.53 (20.09) | | 21.24 (23.56) | | 29.08 (28.52) | | | |  | |  |  |  |
|  | | | Total n/cases | | | 42159/748 | | 136794/2911 | | 132328/2686 | | 141978/2706 | | | | 453259/9051 | |  |  |  |
|  | | | HR (95%-CI), Model 0 | | | 1.00 (Ref) | | 1.20 (1.11-1.30) | | 1.23 (1.14-1.34) | | 1.30 (1.20-1.41) | | | | 1.20 (1.13-1.27) | | <0.0001 |  |  |
|  | | | HR (95%-CI), Model 1 | | | 1.00 (Ref) | | 1.21 (1.12-1.32) | | 1.24 (1.14-1.34) | | 1.29 (1.18-1.40) | | | | 1.18 (1.11-1.25) | | <0.0001 |  |  |
|  | | | HR (95%-CI), Model 2 | | | 1.00 (Ref) | | 1.24 (1.14-1.34) | | 1.26 (1.16-1.37) | | 1.30 (1.19-1.41) | | | | 1.17 (1.11-1.25) | | <0.0001 |  |  |
|  | | | HR (95%-CI), Model 3 | | | 1.00 (Ref) | | 1.10 (1.01-1.20) | | 1.08 (0.99-1.17) | | 1.07 (0.98-1.17) | | | | 1.02 (0.96-1.08) | | 0.5471 |  |  |
|  | | |  | | |  | |  | |  | |  | | | |  | |  |  |  |
| Osteoarthritis | | | Mean grams/day | | | 3.97 (11.40) | | 15.53 (20.11) | | 21.28 (23.62) | | 28.94 (28.41) | | | |  | |  |  |  |
|  | | | Total n/cases | | | 39626/1535 | | 127755/5617 | | 122886/5501 | | 131283/5661 | | | | 421550/18314 | |  |  |  |
|  | | | HR (95%-CI), Model 0 | | | 1.00 (Ref) | | 1.07 (1.01-1.13) | | 1.12 (1.06-1.19) | | 1.15 (1.08-1.22) | | | | 1.12 (1.07-1.17) | | <0.0001 |  |  |
|  | | | HR (95%-CI), Model 1 | | | 1.00 (Ref) | | 1.06 (1.00-1.12) | | 1.10 (1.04-1.17) | | 1.12 (1.05-1.19) | | | | 1.09 (1.05-1.14) | | 0.0001 |  |  |
|  | | | HR (95%-CI), Model 2 | | | 1.00 (Ref) | | 1.09 (1.03-1.16) | | 1.14 (1.08-1.21) | | 1.17 (1.10-1.24) | | | | 1.12 (1.08-1.17) | | <0.0001 |  |  |
|  | | | HR (95%-CI), Model 3 | | | 1.00 (Ref) | | 1.00 (0.94-1.06) | | 1.01 (0.95-1.07) | | 1.00 (0.94-1.06) | | | | 1.00 (0.96-1.04) | | 0.9814 |  |  |
|  | | |  | | |  | |  | |  | |  | | | |  | |  |  |  |
| Kidney stones | | | Mean grams/day | | | 4.11 (11.65) | | 15.53 (20.05) | | 21.28 (23.60) | | 28.97 (28.45) | | | |  | |  |  |  |
|  | | | Total n/cases | | | 43487/190 | | 141626/676 | | 135892/720 | | 144563/894 | | | | 465568/2480 | |  |  |  |
|  | | | HR (95%-CI), Model 0 | | | 1.00 (Ref) | | 1.04 (0.89-1.23) | | 1.03 (0.88-1.22) | | 1.07 (0.91-1.25) | | | | 1.05 (0.94-1.17) | | 0.4186 |  |  |
|  | | | HR (95%-CI), Model 1 | | | 1.00 (Ref) | | 1.11 (0.95-1.31) | | 1.12 (0.95-1.32) | | 1.16 (0.98-1.36) | | | | 1.10 (0.98-1.23) | | 0.1013 |  |  |
|  | | | HR (95%-CI), Model 2 | | | 1.00 (Ref) | | 1.10 (0.94-1.30) | | 1.10 (0.93-1.30) | | 1.12 (0.95-1.32) | | | | 1.07 (0.95-1.20) | | 0.2828 |  |  |
|  | | | HR (95%-CI), Model 3 | | | 1.00 (Ref) | | 1.04 (0.88-1.23) | | 1.01 (0.86-1.20) | | 1.02 (0.86-1.20) | | | | 0.99 (0.89-1.12) | | 0.9257 |  |  |
|  | | |  | | |  | |  | |  | |  | | | |  | |  |  |  |
| Urinary tract infection | | | Mean grams/day | | | 4.12 (11.61) | | 15.58 (20.10) | | 21.32 (23.60) | | 29.08 (28.48) | | | |  | |  |  |  |
|  | | | Total n/cases | | | 43172/486 | | 140985/1506 | | 135870/1559 | | 144947/1748 | | | | 464974/5299 | |  |  |  |
|  | | | HR (95%-CI), Model 0 | | | 1.00 (Ref) | | 0.88 (0.80-0.98) | | 0.95 (0.85-1.05) | | 1.02 (0.92-1.13) | | | | 1.08 (1.00-1.17) | | 0.0378 |  |  |
|  | | | HR (95%-CI), Model 1 | | | 1.00 (Ref) | | 0.94 (0.85-1.05) | | 1.00 (0.90-1.11) | | 1.05 (0.94-1.16) | | | | 1.08 (1.00-1.17) | | 0.0522 |  |  |
|  | | | HR (95%-CI), Model 2 | | | 1.00 (Ref) | | 0.97 (0.88-1.08) | | 1.02 (0.92-1.14) | | 1.07 (0.96-1.19) | | | | 1.09 (1.01-1.18) | | 0.0327 |  |  |
|  | | | HR (95%-CI), Model 3 | | | 1.00 (Ref) | | 0.94 (0.85-1.05) | | 0.98 (0.88-1.09) | | 1.01 (0.90-1.12) | | | | 1.04 (0.96-1.12) | | 0.3682 |  |  |
|  | | |  | | |  | |  | |  | |  | | | |  | |  |  |  |
| Enlarged prostate | | | Mean grams/day | | | 3.82 (11.92) | | 17.14 (22.10) | | 23.39 (25.73) | | 31.98 (30.37) | | | |  | |  |  |  |
|  | | | Total n/cases | | | 11336/193 | | 44230/785 | | 61931/1112 | | 90594/1500 | | | | 208091/3590 | |  |  |  |
|  | | | HR (95%-CI), Model 0 | | | 1.00 (Ref) | | 0.93 (0.80-1.09) | | 0.96 (0.83-1.12) | | 0.93 (0.80-1.08) | | | | 0.97 (0.88-1.06) | | 0.4988 |  |  |
|  | | | HR (95%-CI), Model 1 | | | 1.00 (Ref) | | 1.00 (0.85-1.17) | | 1.05 (0.90-1.23) | | 1.03 (0.89-1.21) | | | | 1.04 (0.94-1.14) | | 0.4874 |  |  |
|  | | | HR (95%-CI), Model 2 | | | 1.00 (Ref) | | 1.01 (0.86-1.19) | | 1.07 (0.91-1.25) | | 1.05 (0.90-1.23) | | | | 1.04 (0.94-1.15) | | 0.4123 |  |  |
|  | | | HR (95%-CI), Model 3 | | | 1.00 (Ref) | | 1.01 (0.86-1.19) | | 1.07 (0.91-1.26) | | 1.06 (0.90-1.24) | | | | 1.05 (0.95-1.16) | | 0.3610 |  |  |
|  | | |  | | |  | |  | |  | |  | | | |  | |  |  |  |
| Female genital prolapse | | | Mean grams/day | | | 4.16 (11.47) | | 14.82 (19.05) | | 19.51 (21.42) | | 24.25 (24.39) | | | |  | |  |  |  |
|  | | | Total n/cases | | | 30742/668 | | 92048/2323 | | 69335/1817 | | 49891/1313 | | | | 242016/6121 | |  |  |  |
|  | | | HR (95%-CI), Model 0 | | | 1.00 (Ref) | | 1.11 (1.02-1.21) | | 1.16 (1.06-1.26) | | 1.18 (1.08-1.30) | | | | 1.14 (1.06-1.22) | | 0.0003 |  |  |
|  | | | HR (95%-CI), Model 1 | | | 1.00 (Ref) | | 1.04 (0.96-1.14) | | 1.05 (0.96-1.15) | | 1.08 (0.98-1.19) | | | | 1.06 (0.99-1.14) | | 0.1110 |  |  |
|  | | | HR (95%-CI), Model 2 | | | 1.00 (Ref) | | 1.07 (0.98-1.17) | | 1.08 (0.99-1.19) | | 1.12 (1.02-1.23) | | | | 1.09 (1.01-1.17) | | 0.0204 |  |  |
|  | | | HR (95%-CI), Model 3 | | | 1.00 (Ref) | | 1.03 (0.94-1.12) | | 1.04 (0.95-1.14) | | 1.06 (0.96-1.17) | | | | 1.05 (0.97-1.12) | | 0.2278 |  |  |
|  | | |  | | |  | |  | |  | |  | | | |  | |  |  |  |
| Uterine fibroids | | | Mean grams/day | | | 3.91 (11.20) | | 14.65 (18.94) | | 19.67 (21.53) | | 24.36 (24.36) | | | |  | |  |  |  |
|  | | | Total n/cases | | | 23633/1127 | | 69107/3493 | | 52613/2746 | | 38130/1993 | | | | 183483/9359 | |  |  |  |
|  | | | HR (95%-CI), Model 0 | | | 1.00 (Ref) | | 1.10 (1.02-1.17) | | 1.11 (1.04-1.19) | | 1.09 (1.01-1.17) | | | | 1.06 (1.00-1.12) | | 0.0354 |  |  |
|  | | | HR (95%-CI), Model 1 | | | 1.00 (Ref) | | 1.12 (1.04-1.20) | | 1.14 (1.06-1.22) | | 1.11 (1.03-1.19) | | | | 1.07 (1.01-1.13) | | 0.0135 |  |  |
|  | | | HR (95%-CI), Model 2 | | | 1.00 (Ref) | | 1.13 (1.06-1.21) | | 1.15 (1.07-1.24) | | 1.12 (1.04-1.21) | | | | 1.07 (1.02-1.13) | | 0.0109 |  |  |
|  | | | HR (95%-CI), Model 3 | | | 1.00 (Ref) | | 1.08 (1.01-1.16) | | 1.08 (1.00-1.16) | | 1.02 (0.95-1.10) | | | | 1.00 (0.95-1.06) | | 0.9818 |  |  |
|  | | |  | | |  | |  | |  | |  | | | |  | |  |  |  |
| Iron deficiency anaemia | | | Mean grams/day | | | 4.12 (11.67) | | 15.58 (20.09) | | 21.30 (23.63) | | 29.06 (28.49) | | | |  | |  |  |  |
|  | | | Total n/cases | | | 43173/562 | | 141679/1351 | | 136517/1423 | | 145734/1464 | | | | 467103/4800 | |  |  |  |
|  | | | HR (95%-CI), Model 0 | | | 1.00 (Ref) | | 0.70 (0.64-0.78) | | 0.78 (0.71-0.87) | | 0.79 (0.72-0.88) | | | | 0.92 (0.85-0.99) | | 0.0293 |  |  |
|  | | | HR (95%-CI), Model 1 | | | 1.00 (Ref) | | 0.81 (0.73-0.90) | | 0.92 (0.83-1.01) | | 0.91 (0.82-1.01) | | | | 0.99 (0.92-1.08) | | 0.8764 |  |  |
|  | | | HR (95%-CI), Model 2 | | | 1.00 (Ref) | | 0.85 (0.77-0.95) | | 0.97 (0.87-1.07) | | 0.95 (0.86-1.05) | | | | 1.02 (0.94-1.10) | | 0.6596 |  |  |
|  | | | HR (95%-CI), Model 3 | | | 1.00 (Ref) | | 0.83 (0.75-0.92) | | 0.93 (0.84-1.03) | | 0.91 (0.81-1.01) | | | | 0.98 (0.91-1.06) | | 0.6382 |  |  |
|  | | |  | | |  | |  | |  | |  | | | |  | |  |  |  |
| Diabetes | | | Mean grams/day | | | 4.08 (11.63) | | 15.50 (20.01) | | 21.18 (23.50) | | 28.76 (28.27) | | | |  | |  |  |  |
|  | | | Total n/cases | | | 42060/710 | | 137006/2436 | | 130655/2822 | | 137630/3823 | | | | 447351/9791 | |  |  |  |
|  | | | HR (95%-CI), Model 0 | | | 1.00 (Ref) | | 0.98 (0.90-1.07) | | 1.12 (1.03-1.22) | | 1.38 (1.27-1.49) | | | | 1.44 (1.35-1.52) | | <0.0001 |  |  |
|  | | | HR (95%-CI), Model 1 | | | 1.00 (Ref) | | 1.19 (1.09-1.30) | | 1.37 (1.26-1.49) | | 1.62 (1.49-1.76) | | | | 1.52 (1.43-1.61) | | <0.0001 |  |  |
|  | | | HR (95%-CI), Model 2 | | | 1.00 (Ref) | | 1.23 (1.13-1.34) | | 1.40 (1.29-1.53) | | 1.64 (1.51-1.79) | | | | 1.52 (1.43-1.61) | | <0.0001 |  |  |
|  | | | HR (95%-CI), Model 3 | | | 1.00 (Ref) | | 1.07 (0.98-1.17) | | 1.15 (1.05-1.25) | | 1.27 (1.16-1.38) | | | | 1.24 (1.17-1.32) | | <0.0001 |  |  |
|  | | |  | | |  | |  | |  | |  | | | |  | |  |  |  |
| Carpal tunnel syndrome | | | Mean grams/day | | | 4.08 (11.63) | | 15.55 (20.08) | | 21.28 (23.60) | | 29.03 (28.45) | | | |  | |  |  |  |
|  | | | Total n/cases | | | 43129/446 | | 140373/1644 | | 135338/1548 | | 144613/1506 | | | | 463453/5144 | |  |  |  |
|  | | | HR (95%-CI), Model 0 | | | 1.00 (Ref) | | 1.13 (1.02-1.26) | | 1.18 (1.06-1.31) | | 1.19 (1.06-1.32) | | | | 1.12 (1.04-1.21) | | 0.0030 |  |  |
|  | | | HR (95%-CI), Model 1 | | | 1.00 (Ref) | | 1.17 (1.05-1.30) | | 1.20 (1.08-1.34) | | 1.18 (1.06-1.31) | | | | 1.10 (1.02-1.19) | | 0.0138 |  |  |
|  | | | HR (95%-CI), Model 2 | | | 1.00 (Ref) | | 1.20 (1.07-1.33) | | 1.26 (1.13-1.40) | | 1.24 (1.11-1.38) | | | | 1.14 (1.06-1.24) | | 0.0009 |  |  |
|  | | | HR (95%-CI), Model 3 | | | 1.00 (Ref) | | 1.10 (0.98-1.22) | | 1.11 (1.00-1.24) | | 1.06 (0.95-1.19) | | | | 1.02 (0.94-1.11) | | 0.5920 |  |  |
|  | | |  | | |  | |  | |  | |  | | | |  | |  |  |  |
| Cataracts | | | Mean grams/day | | | 4.07 (11.59) | | 15.54 (20.05) | | 21.23 (23.52) | | 28.88 (28.31) | | | |  | |  |  |  |
|  | | | Total n/cases | | | 40315/1651 | | 131610/5194 | | 125840/4721 | | 132593/4766 | | | | 430358/16332 | |  |  |  |
|  | | | HR (95%-CI), Model 0 | | | 1.00 (Ref) | | 0.88 (0.83-0.93) | | 0.86 (0.81-0.91) | | 0.89 (0.84-0.95) | | | | 0.94 (0.90-0.98) | | 0.0059 |  |  |
|  | | | HR (95%-CI), Model 1 | | | 1.00 (Ref) | | 0.94 (0.89-0.99) | | 0.93 (0.88-0.98) | | 0.96 (0.90-1.01) | | | | 0.98 (0.94-1.02) | | 0.3292 |  |  |
|  | | | HR (95%-CI), Model 2 | | | 1.00 (Ref) | | 0.95 (0.90-1.00) | | 0.94 (0.88-0.99) | | 0.96 (0.91-1.02) | | | | 0.98 (0.94-1.03) | | 0.4029 |  |  |
|  | | | HR (95%-CI), Model 3 | | | 1.00 (Ref) | | 0.94 (0.89-1.00) | | 0.92 (0.87-0.98) | | 0.94 (0.89-1.00) | | | | 0.97 (0.92-1.01) | | 0.1437 |  |  |
|  | | |  | | |  | |  | |  | |  | | | |  | |  |  |  |
| Cellulitis | | | Mean grams/day | | | 4.10 (11.62) | | 15.55 (20.07) | | 21.27 (23.58) | | 29.05 (28.49) | | | |  | |  |  |  |
|  | | | Total n/cases | | | 43704/295 | | 142464/873 | | 136848/1034 | | 145630/1268 | | | | 468646/3470 | |  |  |  |
|  | | | HR (95%-CI), Model 0 | | | 1.00 (Ref) | | 0.86 (0.76-0.99) | | 1.01 (0.88-1.15) | | 1.10 (0.97-1.25) | | | | 1.20 (1.09-1.32) | | 0.0003 |  |  |
|  | | | HR (95%-CI), Model 1 | | | 1.00 (Ref) | | 0.91 (0.80-1.04) | | 1.05 (0.92-1.20) | | 1.10 (0.96-1.25) | | | | 1.15 (1.05-1.27) | | 0.0034 |  |  |
|  | | | HR (95%-CI), Model 2 | | | 1.00 (Ref) | | 0.94 (0.82-1.08) | | 1.10 (0.96-1.25) | | 1.14 (1.00-1.31) | | | | 1.19 (1.08-1.31) | | 0.0006 |  |  |
|  | | | HR (95%-CI), Model 3 | | | 1.00 (Ref) | | 0.85 (0.74-0.98) | | 0.95 (0.83-1.09) | | 0.96 (0.84-1.10) | | | | 1.04 (0.94-1.15) | | 0.4349 |  |  |
| **Model 0:** Stratified for sex, age group and region and adjusted for age (underlying time variable). **Model 1:** Model 0 additionally adjusted for race (4 groups where possible: White, Asian or Asian British, Black or Black British, Mixed race or other, unknown), deprivation (Townsend index quintiles, unknown), qualification (College or university degree/vocational qualification, National examination at ages 17-18,National examination at age 16, unknown), employment (in paid employment, receiving pension, not in paid employment, unknown), smoking (never, former, current <15 cigarettes/day, current >15 cigarettes/ day, current unknown amount of cigarettes/day, unknown), physical activity (<10 excess METs, 10-<50 excess METs, ≥ 50 excess METs, unknown), alcohol intake (none, < 1 g/day, 1-<10 g, 10-<20 g, ≥20 g/day, unknown), menopausal status (pre-, postmenopausal unknown), hormone-replacement therapy (HRT) (never, past, current, unknown) and oral contraceptive pill intake (OCP) (never, past, current, unknown), and parity (nulliparous, 1-2, 3 or more, unknown). **Model 2:** Model 1 additionally adjusted for total fruit and vegetable intake (<3 servings/day, 3-<4 servings/day, 4-<5 servings/day, ≥6 servings/day, unknown), cereal fibre intake (sex-specific quintiles, unknown), oily fish intake (0 times/week,<1 time/week,1 time/week,>2 times/week, unknown) and non-oily fish intake (<1 time/week, 1 time/week, >2 times/week, unknown). **Model 3:** Model 2 additionally adjusted for BMI (sex-specific quintiles, unknown). | | | | | | | | | | | | | | | | | | |  |  |
| Additional file 1: Table 10. Risk of 25 common conditions by poultry meat intake in UK Biobank. | | | | | | | | | | | | | | | | | | |  |  |
|  | | | |  | | | Poultry meat intake (times/week) | | | | | |  | |  | |  | |  |  |
| Disease | | | | Model | | | 0 - 1 | | 2 | | >3 | |  | | per 30g/day increase | | p-value | |  |  |
| Ischemic heart disease | | | | Mean grams/day | | | 12.17 (24.09) | | 27.63 (32.47) | | 39.49 (38.33) | |  | |  | |  | |  |  |
|  | | | | Total n/cases | | | 69175/2096 | | 155595/4987 | | 210567/6216 | |  | | 435337/13299 | |  | |  |  |
|  | | | | HR (95%-CI), Model 0 | | | 1.00 (Ref) | | 0.99 (0.94-1.04) | | 1.03 (0.98-1.08) | |  | | 1.04 (0.99-1.10) | | 0.1541 | |  |  |
|  | | | | HR (95%-CI), Model 1 | | | 1.00 (Ref) | | 1.05 (0.99-1.10) | | 1.11 (1.05-1.16) | |  | | 1.12 (1.06-1.18) | | <0.0001 | |  |  |
|  | | | | HR (95%-CI), Model 2 | | | 1.00 (Ref) | | 1.06 (1.00-1.11) | | 1.12 (1.06-1.18) | |  | | 1.14 (1.08-1.20) | | <0.0001 | |  |  |
|  | | | | HR (95%-CI), Model 3 | | | 1.00 (Ref) | | 1.03 (0.98-1.09) | | 1.07 (1.01-1.12) | |  | | 1.08 (1.02-1.14) | | 0.0082 | |  |  |
|  | | | |  | | |  | |  | |  | |  | |  | |  | |  |  |
| Atrial Fibrillation and flutter | | | | Mean grams/day | | | 12.19 (24.13) | | 27.61 (32.44) | | 39.47 (38.32) | |  | |  | |  | |  |  |
|  | | | | Total n/cases | | | 69175/727 | | 155595/1756 | | 210567/2310 | |  | | 435337/4793 | |  | |  |  |
|  | | | | HR (95%-CI), Model 0 | | | 1.00 (Ref) | | 1.01 (0.92-1.10) | | 1.13 (1.04-1.22) | |  | | 1.17 (1.07-1.28) | | 0.0008 | |  |  |
|  | | | | HR (95%-CI), Model 1 | | | 1.00 (Ref) | | 1.00 (0.91-1.09) | | 1.11 (1.02-1.21) | |  | | 1.15 (1.05-1.26) | | 0.0019 | |  |  |
|  | | | | HR (95%-CI), Model 2 | | | 1.00 (Ref) | | 1.01 (0.92-1.10) | | 1.11 (1.01-1.20) | |  | | 1.14 (1.04-1.25) | | 0.0048 | |  |  |
|  | | | | HR (95%-CI), Model 3 | | | 1.00 (Ref) | | 0.98 (0.90-1.07) | | 1.05 (0.97-1.15) | |  | | 1.08 (0.99-1.18) | | 0.1012 | |  |  |
|  | | | |  | | |  | |  | |  | |  | |  | |  | |  |  |
| Cerebrovascular | | | | Mean grams/day | | | 12.19 (24.12) | | 27.63 (32.46) | | 39.49 (38.32) | |  | |  | |  | |  |  |
|  | | | | Total n/cases | | | 69175/884 | | 155595/2058 | | 210567/2480 | |  | | 435337/5422 | |  | |  |  |
|  | | | | HR (95%-CI), Model 0 | | | 1.00 (Ref) | | 0.98 (0.91-1.06) | | 0.99 (0.92-1.07) | |  | | 1.00 (0.92-1.09) | | 1.0000 | |  |  |
|  | | | | HR (95%-CI), Model 1 | | | 1.00 (Ref) | | 1.02 (0.94-1.10) | | 1.05 (0.97-1.13) | |  | | 1.06 (0.97-1.15) | | 0.2025 | |  |  |
|  | | | | HR (95%-CI), Model 2 | | | 1.00 (Ref) | | 1.03 (0.95-1.12) | | 1.07 (0.99-1.16) | |  | | 1.08 (0.99-1.17) | | 0.0813 | |  |  |
|  | | | | HR (95%-CI), Model 3 | | | 1.00 (Ref) | | 1.03 (0.95-1.11) | | 1.05 (0.97-1.14) | |  | | 1.06 (0.97-1.15) | | 0.1996 | |  |  |
|  | | | |  | | |  | |  | |  | |  | |  | |  | |  |  |
| Ischaemic stroke | | | | Mean grams/day | | | 12.18 (24.12) | | 27.63 (32.46) | | 39.47 (38.31) | |  | |  | |  | |  |  |
|  | | | | Total n/cases | | | 69175/404 | | 155595/922 | | 210567/1062 | |  | | 435337/2388 | |  | |  |  |
|  | | | | HR (95%-CI), Model 0 | | | 1.00 (Ref) | | 0.95 (0.85-1.07) | | 0.94 (0.84-1.06) | |  | | 0.94 (0.83-1.06) | | 0.3251 | |  |  |
|  | | | | HR (95%-CI), Model 1 | | | 1.00 (Ref) | | 1.01 (0.89-1.13) | | 1.02 (0.91-1.14) | |  | | 1.02 (0.90-1.16) | | 0.7332 | |  |  |
|  | | | | HR (95%-CI), Model 2 | | | 1.00 (Ref) | | 1.04 (0.93-1.17) | | 1.07 (0.95-1.20) | |  | | 1.07 (0.94-1.21) | | 0.2889 | |  |  |
|  | | | | HR (95%-CI), Model 3 | | | 1.00 (Ref) | | 1.02 (0.91-1.15) | | 1.03 (0.91-1.16) | |  | | 1.03 (0.91-1.17) | | 0.6437 | |  |  |
|  | | | |  | | |  | |  | |  | |  | |  | |  | |  |  |
| Haemorrhagic stroke | | | | Mean grams/day | | | 12.19 (24.12) | | 27.62 (32.45) | | 39.48 (38.31) | |  | |  | |  | |  |  |
|  | | | | Total n/cases | | | 69175/160 | | 155595/347 | | 210567/450 | |  | | 435337/957 | |  | |  |  |
|  | | | | HR (95%-CI), Model 0 | | | 1.00 (Ref) | | 0.94 (0.78-1.14) | | 0.99 (0.82-1.18) | |  | | 1.00 (0.83-1.22) | | 0.9636 | |  |  |
|  | | | | HR (95%-CI), Model 1 | | | 1.00 (Ref) | | 0.97 (0.81-1.17) | | 1.04 (0.86-1.24) | |  | | 1.06 (0.87-1.29) | | 0.5868 | |  |  |
|  | | | | HR (95%-CI), Model 2 | | | 1.00 (Ref) | | 0.98 (0.81-1.19) | | 1.03 (0.86-1.25) | |  | | 1.05 (0.86-1.29) | | 0.6163 | |  |  |
|  | | | | HR (95%-CI), Model 3 | | | 1.00 (Ref) | | 0.99 (0.82-1.20) | | 1.05 (0.87-1.27) | |  | | 1.07 (0.88-1.31) | | 0.4991 | |  |  |
|  | | | |  | | |  | |  | |  | |  | |  | |  | |  |  |
| Venous thromboembolism | | | | Mean grams/day | | | 12.27 (24.22) | | 27.45 (32.37) | | 39.29 (38.32) | |  | |  | |  | |  |  |
|  | | | | Total n/cases | | | 73329/583 | | 165647/1461 | | 223536/1774 | |  | | 462512/3818 | |  | |  |  |
|  | | | | HR (95%-CI), Model 0 | | | 1.00 (Ref) | | 1.06 (0.96-1.16) | | 1.04 (0.95-1.15) | |  | | 1.03 (0.94-1.14) | | 0.5019 | |  |  |
|  | | | | HR (95%-CI), Model 1 | | | 1.00 (Ref) | | 1.08 (0.98-1.19) | | 1.09 (0.99-1.19) | |  | | 1.08 (0.98-1.19) | | 0.1316 | |  |  |
|  | | | | HR (95%-CI), Model 2 | | | 1.00 (Ref) | | 1.10 (1.00-1.22) | | 1.11 (1.01-1.22) | |  | | 1.10 (0.99-1.22) | | 0.0643 | |  |  |
|  | | | | HR (95%-CI), Model 3 | | | 1.00 (Ref) | | 1.07 (0.97-1.17) | | 1.04 (0.94-1.14) | |  | | 1.02 (0.92-1.13) | | 0.6859 | |  |  |
|  | | | |  | | |  | |  | |  | |  | |  | |  | |  |  |
| Varicose veins | | | | Mean grams/day | | | 12.25 (24.16) | | 27.44 (32.38) | | 39.34 (38.39) | |  | |  | |  | |  |  |
|  | | | | Total n/cases | | | 71629/410 | | 161126/940 | | 217783/1311 | |  | | 450538/2661 | |  | |  |  |
|  | | | | HR (95%-CI), Model 0 | | | 1.00 (Ref) | | 1.01 (0.90-1.14) | | 1.05 (0.94-1.17) | |  | | 1.06 (0.94-1.19) | | 0.3673 | |  |  |
|  | | | | HR (95%-CI), Model 1 | | | 1.00 (Ref) | | 1.01 (0.89-1.13) | | 1.03 (0.92-1.16) | |  | | 1.04 (0.92-1.17) | | 0.5101 | |  |  |
|  | | | | HR (95%-CI), Model 2 | | | 1.00 (Ref) | | 1.01 (0.90-1.14) | | 1.03 (0.91-1.15) | |  | | 1.03 (0.91-1.16) | | 0.6366 | |  |  |
|  | | | | HR (95%-CI), Model 3 | | | 1.00 (Ref) | | 0.99 (0.88-1.12) | | 1.00 (0.89-1.12) | |  | | 1.00 (0.88-1.13) | | 0.9440 | |  |  |
|  | | | |  | | |  | |  | |  | |  | |  | |  | |  |  |
| Haemorrhoids | | | | Mean grams/day | | | 12.30 (24.25) | | 27.40 (32.43) | | 39.36 (38.42) | |  | |  | |  | |  |  |
|  | | | | Total n/cases | | | 71869/1272 | | 162329/2943 | | 218937/4200 | |  | | 453135/8415 | |  | |  |  |
|  | | | | HR (95%-CI), Model 0 | | | 1.00 (Ref) | | 1.01 (0.95-1.08) | | 1.05 (0.99-1.12) | |  | | 1.06 (0.99-1.14) | | 0.0707 | |  |  |
|  | | | | HR (95%-CI), Model 1 | | | 1.00 (Ref) | | 1.02 (0.95-1.09) | | 1.05 (0.99-1.12) | |  | | 1.06 (0.99-1.14) | | 0.0886 | |  |  |
|  | | | | HR (95%-CI), Model 2 | | | 1.00 (Ref) | | 1.03 (0.96-1.10) | | 1.06 (0.99-1.13) | |  | | 1.07 (1.00-1.15) | | 0.0535 | |  |  |
|  | | | | HR (95%-CI), Model 3 | | | 1.00 (Ref) | | 1.03 (0.96-1.10) | | 1.06 (0.99-1.13) | |  | | 1.07 (1.00-1.15) | | 0.0543 | |  |  |
|  | | | |  | | |  | |  | |  | |  | |  | |  | |  |  |
| Pneumonia | | | | Mean grams/day | | | 12.26 (24.23) | | 27.45 (32.38) | | 39.33 (38.35) | |  | |  | |  | |  |  |
|  | | | | Total n/cases | | | 73543/1157 | | 166340/2449 | | 224574/2899 | |  | | 464457/6505 | |  | |  |  |
|  | | | | HR (95%-CI), Model 0 | | | 1.00 (Ref) | | 0.90 (0.84-0.96) | | 0.88 (0.82-0.94) | |  | | 0.88 (0.82-0.95) | | 0.0009 | |  |  |
|  | | | | HR (95%-CI), Model 1 | | | 1.00 (Ref) | | 0.98 (0.91-1.05) | | 1.00 (0.93-1.07) | |  | | 1.00 (0.93-1.08) | | 0.9329 | |  |  |
|  | | | | HR (95%-CI), Model 2 | | | 1.00 (Ref) | | 1.00 (0.93-1.07) | | 1.03 (0.96-1.11) | |  | | 1.04 (0.96-1.12) | | 0.3130 | |  |  |
|  | | | | HR (95%-CI), Model 3 | | | 1.00 (Ref) | | 1.00 (0.93-1.08) | | 1.02 (0.96-1.10) | |  | | 1.03 (0.96-1.11) | | 0.4236 | |  |  |
|  | | | |  | | |  | |  | |  | |  | |  | |  | |  |  |
| Gastro-oesophageal reflux disease | | | | Mean grams/day | | | 12.28 (24.18) | | 27.43 (32.40) | | 39.38 (38.43) | |  | |  | |  | |  |  |
|  | | | | Total n/cases | | | 70796/1099 | | 159339/2432 | | 214566/3590 | |  | | 444701/7121 | |  | |  |  |
|  | | | | HR (95%-CI), Model 0 | | | 1.00 (Ref) | | 0.96 (0.90-1.04) | | 1.09 (1.02-1.17) | |  | | 1.14 (1.06-1.23) | | 0.0005 | |  |  |
|  | | | | HR (95%-CI), Model 1 | | | 1.00 (Ref) | | 0.98 (0.91-1.05) | | 1.11 (1.03-1.19) | |  | | 1.16 (1.08-1.25) | | 0.0001 | |  |  |
|  | | | | HR (95%-CI), Model 2 | | | 1.00 (Ref) | | 1.00 (0.93-1.08) | | 1.14 (1.07-1.23) | |  | | 1.20 (1.11-1.29) | | <0.0001 | |  |  |
|  | | | | HR (95%-CI), Model 3 | | | 1.00 (Ref) | | 0.99 (0.92-1.06) | | 1.12 (1.04-1.20) | |  | | 1.17 (1.09-1.26) | | <0.0001 | |  |  |
|  | | | |  | | |  | |  | |  | |  | |  | |  | |  |  |
| Gastritis and duodenitis | | | | Mean grams/day | | | 12.23 (24.21) | | 27.47 (32.37) | | 39.33 (38.40) | |  | |  | |  | |  |  |
|  | | | | Total n/cases | | | 72346/1770 | | 163275/4116 | | 220356/5649 | |  | | 455977/11535 | |  | |  |  |
|  | | | | HR (95%-CI), Model 0 | | | 1.00 (Ref) | | 1.01 (0.96-1.07) | | 1.04 (0.99-1.10) | |  | | 1.05 (1.00-1.12) | | 0.0691 | |  |  |
|  | | | | HR (95%-CI), Model 1 | | | 1.00 (Ref) | | 1.05 (1.00-1.12) | | 1.09 (1.04-1.15) | |  | | 1.10 (1.04-1.17) | | 0.0008 | |  |  |
|  | | | | HR (95%-CI), Model 2 | | | 1.00 (Ref) | | 1.08 (1.02-1.14) | | 1.12 (1.06-1.18) | |  | | 1.13 (1.06-1.19) | | 0.0001 | |  |  |
|  | | | | HR (95%-CI), Model 3 | | | 1.00 (Ref) | | 1.07 (1.01-1.14) | | 1.11 (1.05-1.17) | |  | | 1.12 (1.05-1.18) | | 0.0002 | |  |  |
|  | | | |  | | |  | |  | |  | |  | |  | |  | |  |  |
| Inguinal hernia | | | | Mean grams/day | | | 12.12 (24.05) | | 27.48 (32.42) | | 39.35 (38.33) | |  | |  | |  | |  |  |
|  | | | | Total n/cases | | | 70659/1301 | | 159040/3141 | | 216071/3668 | |  | | 445770/8110 | |  | |  |  |
|  | | | | HR (95%-CI), Model 0 | | | 1.00 (Ref) | | 0.99 (0.93-1.06) | | 0.96 (0.90-1.02) | |  | | 0.95 (0.89-1.02) | | 0.1476 | |  |  |
|  | | | | HR (95%-CI), Model 1 | | | 1.00 (Ref) | | 0.98 (0.92-1.05) | | 0.95 (0.89-1.01) | |  | | 0.94 (0.88-1.00) | | 0.0637 | |  |  |
|  | | | | HR (95%-CI), Model 2 | | | 1.00 (Ref) | | 0.98 (0.92-1.05) | | 0.95 (0.89-1.01) | |  | | 0.94 (0.87-1.00) | | 0.0607 | |  |  |
|  | | | | HR (95%-CI), Model 3 | | | 1.00 (Ref) | | 1.02 (0.95-1.09) | | 1.01 (0.95-1.08) | |  | | 1.01 (0.94-1.08) | | 0.8681 | |  |  |
|  | | | |  | | |  | |  | |  | |  | |  | |  | |  |  |
| Noninfective enteritis and colitis | | | | Mean grams/day | | | 12.23 (24.12) | | 27.46 (32.42) | | 39.28 (38.29) | |  | |  | |  | |  |  |
|  | | | | Total n/cases | | | 72732/1047 | | 164534/2227 | | 221443/3058 | |  | | 458709/6332 | |  | |  |  |
|  | | | | HR (95%-CI), Model 0 | | | 1.00 (Ref) | | 0.93 (0.86-1.00) | | 0.97 (0.91-1.05) | |  | | 0.99 (0.92-1.07) | | 0.8662 | |  |  |
|  | | | | HR (95%-CI), Model 1 | | | 1.00 (Ref) | | 0.95 (0.89-1.03) | | 1.01 (0.94-1.09) | |  | | 1.03 (0.96-1.12) | | 0.4038 | |  |  |
|  | | | | HR (95%-CI), Model 2 | | | 1.00 (Ref) | | 0.98 (0.90-1.05) | | 1.03 (0.96-1.11) | |  | | 1.05 (0.97-1.14) | | 0.1914 | |  |  |
|  | | | | HR (95%-CI), Model 3 | | | 1.00 (Ref) | | 0.97 (0.90-1.05) | | 1.02 (0.95-1.10) | |  | | 1.04 (0.96-1.13) | | 0.3108 | |  |  |
|  | | | |  | | |  | |  | |  | |  | |  | |  | |  |  |
| Diverticular disease | | | | Mean grams/day | | | 12.30 (24.23) | | 27.43 (32.25) | | 39.39 (38.39) | |  | |  | |  | |  |  |
|  | | | | Total n/cases | | | 73133/1887 | | 164754/4773 | | 222480/6323 | |  | | 460367/12983 | |  | |  |  |
|  | | | | HR (95%-CI), Model 0 | | | 1.00 (Ref) | | 1.08 (1.03-1.14) | | 1.13 (1.08-1.19) | |  | | 1.14 (1.08-1.21) | | <0.0001 | |  |  |
|  | | | | HR (95%-CI), Model 1 | | | 1.00 (Ref) | | 1.07 (1.02-1.13) | | 1.13 (1.07-1.19) | |  | | 1.14 (1.08-1.20) | | <0.0001 | |  |  |
|  | | | | HR (95%-CI), Model 2 | | | 1.00 (Ref) | | 1.08 (1.03-1.14) | | 1.14 (1.08-1.20) | |  | | 1.15 (1.09-1.22) | | <0.0001 | |  |  |
|  | | | | HR (95%-CI), Model 3 | | | 1.00 (Ref) | | 1.06 (1.00-1.12) | | 1.09 (1.04-1.15) | |  | | 1.10 (1.04-1.17) | | 0.0007 | |  |  |
|  | | | |  | | |  | |  | |  | |  | |  | |  | |  |  |
| Colon polyps | | | | Mean grams/day | | | 12.20 (24.17) | | 27.42 (32.30) | | 39.34 (38.38) | |  | |  | |  | |  |  |
|  | | | | Total n/cases | | | 71500/5887 | | 161643/13874 | | 217881/18099 | |  | | 451024/37860 | |  | |  |  |
|  | | | | HR (95%-CI), Model 0 | | | 1.00 (Ref) | | 1.02 (0.99-1.05) | | 1.04 (1.01-1.07) | |  | | 1.04 (1.01-1.08) | | 0.0095 | |  |  |
|  | | | | HR (95%-CI), Model 1 | | | 1.00 (Ref) | | 1.03 (1.00-1.06) | | 1.05 (1.02-1.09) | |  | | 1.06 (1.03-1.10) | | 0.0002 | |  |  |
|  | | | | HR (95%-CI), Model 2 | | | 1.00 (Ref) | | 1.04 (1.01-1.07) | | 1.06 (1.03-1.10) | |  | | 1.07 (1.04-1.11) | | <0.0001 | |  |  |
|  | | | | HR (95%-CI), Model 3 | | | 1.00 (Ref) | | 1.03 (0.99-1.06) | | 1.04 (1.01-1.08) | |  | | 1.05 (1.01-1.08) | | 0.0052 | |  |  |
|  | | | |  | | |  | |  | |  | |  | |  | |  | |  |  |
| Gallbladder disease | | | | Mean grams/day | | | 12.21 (24.13) | | 27.42 (32.37) | | 39.34 (38.41) | |  | |  | |  | |  |  |
|  | | | | Total n/cases | | | 72272/1342 | | 162662/3136 | | 218492/4577 | |  | | 453426/9055 | |  | |  |  |
|  | | | | HR (95%-CI), Model 0 | | | 1.00 (Ref) | | 1.03 (0.97-1.10) | | 1.15 (1.08-1.22) | |  | | 1.19 (1.11-1.27) | | <0.0001 | |  |  |
|  | | | | HR (95%-CI), Model 1 | | | 1.00 (Ref) | | 1.05 (0.99-1.12) | | 1.18 (1.11-1.26) | |  | | 1.22 (1.15-1.31) | | <0.0001 | |  |  |
|  | | | | HR (95%-CI), Model 2 | | | 1.00 (Ref) | | 1.09 (1.02-1.16) | | 1.23 (1.15-1.31) | |  | | 1.27 (1.19-1.36) | | <0.0001 | |  |  |
|  | | | | HR (95%-CI), Model 3 | | | 1.00 (Ref) | | 1.02 (0.95-1.09) | | 1.09 (1.02-1.16) | |  | | 1.11 (1.04-1.19) | | 0.0018 | |  |  |
|  | | | |  | | |  | |  | |  | |  | |  | |  | |  |  |
| Osteoarthritis | | | | Mean grams/day | | | 12.05 (23.97) | | 27.38 (32.28) | | 39.43 (38.42) | |  | |  | |  | |  |  |
|  | | | | Total n/cases | | | 67217/2681 | | 150676/6688 | | 203802/8935 | |  | | 421695/18304 | |  | |  |  |
|  | | | | HR (95%-CI), Model 0 | | | 1.00 (Ref) | | 1.08 (1.03-1.13) | | 1.18 (1.13-1.23) | |  | | 1.21 (1.15-1.27) | | <0.0001 | |  |  |
|  | | | | HR (95%-CI), Model 1 | | | 1.00 (Ref) | | 1.07 (1.02-1.12) | | 1.17 (1.12-1.22) | |  | | 1.19 (1.14-1.25) | | <0.0001 | |  |  |
|  | | | | HR (95%-CI), Model 2 | | | 1.00 (Ref) | | 1.08 (1.03-1.13) | | 1.16 (1.11-1.22) | |  | | 1.19 (1.13-1.24) | | <0.0001 | |  |  |
|  | | | | HR (95%-CI), Model 3 | | | 1.00 (Ref) | | 1.02 (0.98-1.07) | | 1.06 (1.02-1.11) | |  | | 1.07 (1.03-1.13) | | 0.0025 | |  |  |
|  | | | |  | | |  | |  | |  | |  | |  | |  | |  |  |
| Kidney stones | | | | Mean grams/day | | | 12.23 (24.17) | | 27.47 (32.40) | | 39.32 (38.37) | |  | |  | |  | |  |  |
|  | | | | Total n/cases | | | 73956/361 | | 166854/884 | | 224927/1238 | |  | | 465737/2483 | |  | |  |  |
|  | | | | HR (95%-CI), Model 0 | | | 1.00 (Ref) | | 1.05 (0.93-1.19) | | 1.10 (0.98-1.24) | |  | | 1.12 (0.99-1.27) | | 0.0835 | |  |  |
|  | | | | HR (95%-CI), Model 1 | | | 1.00 (Ref) | | 1.11 (0.98-1.26) | | 1.18 (1.05-1.33) | |  | | 1.19 (1.05-1.35) | | 0.0063 | |  |  |
|  | | | | HR (95%-CI), Model 2 | | | 1.00 (Ref) | | 1.11 (0.98-1.26) | | 1.19 (1.05-1.34) | |  | | 1.21 (1.06-1.37) | | 0.0041 | |  |  |
|  | | | | HR (95%-CI), Model 3 | | | 1.00 (Ref) | | 1.08 (0.95-1.22) | | 1.12 (0.99-1.27) | |  | | 1.13 (0.99-1.28) | | 0.0622 | |  |  |
|  | | | |  | | |  | |  | |  | |  | |  | |  | |  |  |
| Urinary tract infection | | | | Mean grams/day | | | 12.27 (24.19) | | 27.47 (32.37) | | 39.28 (38.34) | |  | |  | |  | |  |  |
|  | | | | Total n/cases | | | 73625/901 | | 166616/1955 | | 224888/2446 | |  | | 465129/5302 | |  | |  |  |
|  | | | | HR (95%-CI), Model 0 | | | 1.00 (Ref) | | 0.92 (0.85-1.00) | | 0.93 (0.86-1.01) | |  | | 0.94 (0.86-1.02) | | 0.1428 | |  |  |
|  | | | | HR (95%-CI), Model 1 | | | 1.00 (Ref) | | 0.97 (0.90-1.05) | | 0.99 (0.92-1.07) | |  | | 1.00 (0.92-1.09) | | 0.9980 | |  |  |
|  | | | | HR (95%-CI), Model 2 | | | 1.00 (Ref) | | 0.98 (0.91-1.06) | | 1.00 (0.92-1.08) | |  | | 1.00 (0.92-1.09) | | 0.9212 | |  |  |
|  | | | | HR (95%-CI), Model 3 | | | 1.00 (Ref) | | 0.97 (0.89-1.05) | | 0.97 (0.89-1.05) | |  | | 0.97 (0.89-1.05) | | 0.4449 | |  |  |
|  | | | |  | | |  | |  | |  | |  | |  | |  | |  |  |
| Enlarged prostate | | | | Mean grams/day | | | 13.24 (24.97) | | 27.01 (33.20) | | 39.31 (39.44) | |  | |  | |  | |  |  |
|  | | | | Total n/cases | | | 31496/577 | | 76672/1416 | | 99945/1599 | |  | | 208113/3592 | |  | |  |  |
|  | | | | HR (95%-CI), Model 0 | | | 1.00 (Ref) | | 0.99 (0.90-1.09) | | 0.98 (0.89-1.08) | |  | | 0.98 (0.88-1.08) | | 0.6374 | |  |  |
|  | | | | HR (95%-CI), Model 1 | | | 1.00 (Ref) | | 1.02 (0.93-1.13) | | 1.02 (0.93-1.12) | |  | | 1.02 (0.92-1.13) | | 0.7598 | |  |  |
|  | | | | HR (95%-CI), Model 2 | | | 1.00 (Ref) | | 1.03 (0.94-1.14) | | 1.03 (0.93-1.13) | |  | | 1.02 (0.92-1.14) | | 0.6751 | |  |  |
|  | | | | HR (95%-CI), Model 3 | | | 1.00 (Ref) | | 1.03 (0.94-1.14) | | 1.03 (0.93-1.13) | |  | | 1.03 (0.92-1.14) | | 0.6424 | |  |  |
|  | | | |  | | |  | |  | |  | |  | |  | |  | |  |  |
| Female genital prolapse | | | | Mean grams/day | | | 11.58 (23.71) | | 27.93 (31.82) | | 39.52 (37.48) | |  | |  | |  | |  |  |
|  | | | | Total n/cases | | | 40099/878 | | 84287/2100 | | 117775/3148 | |  | | 242161/6126 | |  | |  |  |
|  | | | | HR (95%-CI), Model 0 | | | 1.00 (Ref) | | 1.09 (1.01-1.18) | | 1.23 (1.14-1.32) | |  | | 1.27 (1.17-1.38) | | <0.0001 | |  |  |
|  | | | | HR (95%-CI), Model 1 | | | 1.00 (Ref) | | 0.99 (0.91-1.07) | | 1.07 (0.99-1.16) | |  | | 1.11 (1.02-1.20) | | 0.0141 | |  |  |
|  | | | | HR (95%-CI), Model 2 | | | 1.00 (Ref) | | 1.01 (0.93-1.09) | | 1.10 (1.02-1.18) | |  | | 1.13 (1.04-1.23) | | 0.0033 | |  |  |
|  | | | | HR (95%-CI), Model 3 | | | 1.00 (Ref) | | 0.98 (0.91-1.06) | | 1.05 (0.97-1.13) | |  | | 1.08 (0.99-1.17) | | 0.0714 | |  |  |
|  | | | |  | | |  | |  | |  | |  | |  | |  | |  |  |
| Uterine fibroids | | | | Mean grams/day | | | 11.32 (23.37) | | 27.76 (31.56) | | 39.35 (37.41) | |  | |  | |  | |  |  |
|  | | | | Total n/cases | | | 31179/1533 | | 63704/3067 | | 88697/4765 | |  | | 183580/9365 | |  | |  |  |
|  | | | | HR (95%-CI), Model 0 | | | 1.00 (Ref) | | 1.01 (0.95-1.07) | | 1.07 (1.01-1.13) | |  | | 1.09 (1.02-1.16) | | 0.0089 | |  |  |
|  | | | | HR (95%-CI), Model 1 | | | 1.00 (Ref) | | 1.02 (0.96-1.09) | | 1.07 (1.01-1.13) | |  | | 1.08 (1.02-1.16) | | 0.0118 | |  |  |
|  | | | | HR (95%-CI), Model 2 | | | 1.00 (Ref) | | 1.03 (0.97-1.10) | | 1.08 (1.02-1.14) | |  | | 1.09 (1.03-1.17) | | 0.0066 | |  |  |
|  | | | | HR (95%-CI), Model 3 | | | 1.00 (Ref) | | 0.99 (0.93-1.06) | | 1.01 (0.95-1.07) | |  | | 1.01 (0.95-1.08) | | 0.6656 | |  |  |
|  | | | |  | | |  | |  | |  | |  | |  | |  | |  |  |
| Iron deficiency anaemia | | | | Mean grams/day | | | 12.30 (24.20) | | 27.47 (32.42) | | 39.34 (38.37) | |  | |  | |  | |  |  |
|  | | | | Total n/cases | | | 73817/920 | | 167438/1785 | | 226009/2091 | |  | | 467264/4796 | |  | |  |  |
|  | | | | HR (95%-CI), Model 0 | | | 1.00 (Ref) | | 0.84 (0.78-0.91) | | 0.76 (0.70-0.82) | |  | | 0.74 (0.68-0.81) | | <0.0001 | |  |  |
|  | | | | HR (95%-CI), Model 1 | | | 1.00 (Ref) | | 0.93 (0.85-1.00) | | 0.84 (0.78-0.91) | |  | | 0.82 (0.76-0.90) | | <0.0001 | |  |  |
|  | | | | HR (95%-CI), Model 2 | | | 1.00 (Ref) | | 0.96 (0.89-1.05) | | 0.87 (0.81-0.95) | |  | | 0.85 (0.78-0.93) | | 0.0003 | |  |  |
|  | | | | HR (95%-CI), Model 3 | | | 1.00 (Ref) | | 0.95 (0.88-1.03) | | 0.85 (0.78-0.92) | |  | | 0.83 (0.76-0.90) | | <0.0001 | |  |  |
|  | | | |  | | |  | |  | |  | |  | |  | |  | |  |  |
| Diabetes | | | | Mean grams/day | | | 12.20 (24.16) | | 27.58 (32.45) | | 39.38 (38.22) | |  | |  | |  | |  |  |
|  | | | | Total n/cases | | | 70894/1481 | | 160465/3499 | | 216144/4819 | |  | | 447503/9799 | |  | |  |  |
|  | | | | HR (95%-CI), Model 0 | | | 1.00 (Ref) | | 1.00 (0.95-1.07) | | 1.12 (1.05-1.18) | |  | | 1.16 (1.09-1.23) | | <0.0001 | |  |  |
|  | | | | HR (95%-CI), Model 1 | | | 1.00 (Ref) | | 1.13 (1.07-1.21) | | 1.28 (1.21-1.36) | |  | | 1.32 (1.24-1.41) | | <0.0001 | |  |  |
|  | | | | HR (95%-CI), Model 2 | | | 1.00 (Ref) | | 1.15 (1.08-1.22) | | 1.30 (1.23-1.39) | |  | | 1.35 (1.26-1.44) | | <0.0001 | |  |  |
|  | | | | HR (95%-CI), Model 3 | | | 1.00 (Ref) | | 1.07 (1.00-1.13) | | 1.12 (1.06-1.19) | |  | | 1.14 (1.07-1.21) | | 0.0001 | |  |  |
|  | | | |  | | |  | |  | |  | |  | |  | |  | |  |  |
| Carpal tunnel syndrome | | | | Mean grams/day | | | 12.24 (24.21) | | 27.42 (32.37) | | 39.31 (38.34) | |  | |  | |  | |  |  |
|  | | | | Total n/cases | | | 73594/775 | | 166145/1816 | | 223872/2555 | |  | | 463611/5146 | |  | |  |  |
|  | | | | HR (95%-CI), Model 0 | | | 1.00 (Ref) | | 1.04 (0.95-1.13) | | 1.11 (1.02-1.20) | |  | | 1.13 (1.03-1.23) | | 0.0069 | |  |  |
|  | | | | HR (95%-CI), Model 1 | | | 1.00 (Ref) | | 1.06 (0.98-1.16) | | 1.13 (1.04-1.23) | |  | | 1.15 (1.06-1.26) | | 0.0014 | |  |  |
|  | | | | HR (95%-CI), Model 2 | | | 1.00 (Ref) | | 1.08 (0.99-1.18) | | 1.14 (1.05-1.24) | |  | | 1.15 (1.06-1.26) | | 0.0015 | |  |  |
|  | | | | HR (95%-CI), Model 3 | | | 1.00 (Ref) | | 1.03 (0.94-1.12) | | 1.04 (0.95-1.13) | |  | | 1.04 (0.95-1.13) | | 0.4186 | |  |  |
|  | | | |  | | |  | |  | |  | |  | |  | |  | |  |  |
| Cataracts | | | | Mean grams/day | | | 12.12 (24.06) | | 27.70 (32.48) | | 39.45 (38.31) | |  | |  | |  | |  |  |
|  | | | | Total n/cases | | | 67820/2800 | | 154075/6295 | | 208624/7254 | |  | | 430519/16349 | |  | |  |  |
|  | | | | HR (95%-CI), Model 0 | | | 1.00 (Ref) | | 0.93 (0.89-0.97) | | 0.93 (0.89-0.97) | |  | | 0.93 (0.89-0.98) | | 0.0053 | |  |  |
|  | | | | HR (95%-CI), Model 1 | | | 1.00 (Ref) | | 0.97 (0.93-1.01) | | 0.97 (0.92-1.01) | |  | | 0.97 (0.92-1.01) | | 0.1623 | |  |  |
|  | | | | HR (95%-CI), Model 2 | | | 1.00 (Ref) | | 0.98 (0.93-1.02) | | 0.97 (0.93-1.02) | |  | | 0.97 (0.93-1.02) | | 0.2587 | |  |  |
|  | | | | HR (95%-CI), Model 3 | | | 1.00 (Ref) | | 0.97 (0.93-1.02) | | 0.96 (0.92-1.01) | |  | | 0.96 (0.92-1.01) | | 0.1192 | |  |  |
|  | | | |  | | |  | |  | |  | |  | |  | |  | |  |  |
| Cellulitis | | | | Mean grams/day | | | 12.25 (24.18) | | 27.45 (32.37) | | 39.31 (38.36) | |  | |  | |  | |  |  |
|  | | | | Total n/cases | | | 74329/546 | | 167971/1234 | | 226517/1684 | |  | | 468817/3464 | |  | |  |  |
|  | | | | HR (95%-CI), Model 0 | | | 1.00 (Ref) | | 0.97 (0.88-1.07) | | 1.02 (0.93-1.13) | |  | | 1.04 (0.94-1.16) | | 0.4341 | |  |  |
|  | | | | HR (95%-CI), Model 1 | | | 1.00 (Ref) | | 1.03 (0.93-1.14) | | 1.12 (1.01-1.23) | |  | | 1.14 (1.03-1.27) | | 0.0120 | |  |  |
|  | | | | HR (95%-CI), Model 2 | | | 1.00 (Ref) | | 1.05 (0.94-1.16) | | 1.13 (1.02-1.24) | |  | | 1.15 (1.03-1.28) | | 0.0097 | |  |  |
|  | | | | HR (95%-CI), Model 3 | | | 1.00 (Ref) | | 1.00 (0.90-1.10) | | 1.02 (0.92-1.13) | |  | | 1.03 (0.92-1.14) | | 0.6162 | |  |  |
| **Model 0:** Stratified for sex, age group and region and adjusted for age (underlying time variable). **Model 1:** Model 0 additionally adjusted for race (4 groups where possible: White, Asian or Asian British, Black or Black British, Mixed race or other, unknown), deprivation (Townsend index quintiles, unknown), qualification (College or university degree/vocational qualification, National examination at ages 17-18,National examination at age 16, unknown), employment (in paid employment, receiving pension, not in paid employment, unknown), smoking (never, former, current <15 cigarettes/day, current >15 cigarettes/ day, current unknown amount of cigarettes/day, unknown), physical activity (<10 excess METs, 10-<50 excess METs, ≥ 50 excess METs, unknown), alcohol intake (none, < 1 g/day, 1-<10 g, 10-<20 g, ≥20 g/day, unknown), menopausal status (pre-, postmenopausal unknown), hormone-replacement therapy (HRT) (never, past, current, unknown) and oral contraceptive pill intake (OCP) (never, past, current, unknown), and parity (nulliparous, 1-2, 3 or more, unknown). **Model 2:** Model 1 additionally adjusted for total fruit and vegetable intake (<3 servings/day, 3-<4 servings/day, 4-<5 servings/day, ≥6 servings/day, unknown), cereal fibre intake (sex-specific quintiles, unknown), oily fish intake (0 times/week,<1 time/week,1 time/week,>2 times/week, unknown) and non-oily fish intake (<1 time/week, 1 time/week, >2 times/week, unknown). **Model 3:** Model 2 additionally adjusted for BMI (sex-specific quintiles, unknown). | | | | | | | | | | | | | | | | | | |  |  |

# Additional file 1: Figures

503 317 participants recruited (2006-2010)

Exclusion criteria:

- Withdrawn consent (n=824)
- Prior history of malignant cancer (n=27 174)
- Genetic sex differed to reported gender (n=334)

474 985 Maximal study sample

Total number of participants with available data by meat type

- Total meat (n=467 384)
- Unprocessed red and processed meat: (n= 467 741)
- Unprocessed red meat (n=468 328)
- Processed meat (n=472 844)
- Poultry meat (n=473 011)

## Additional file 1: Fig. 1. Participant flow chart of the study.


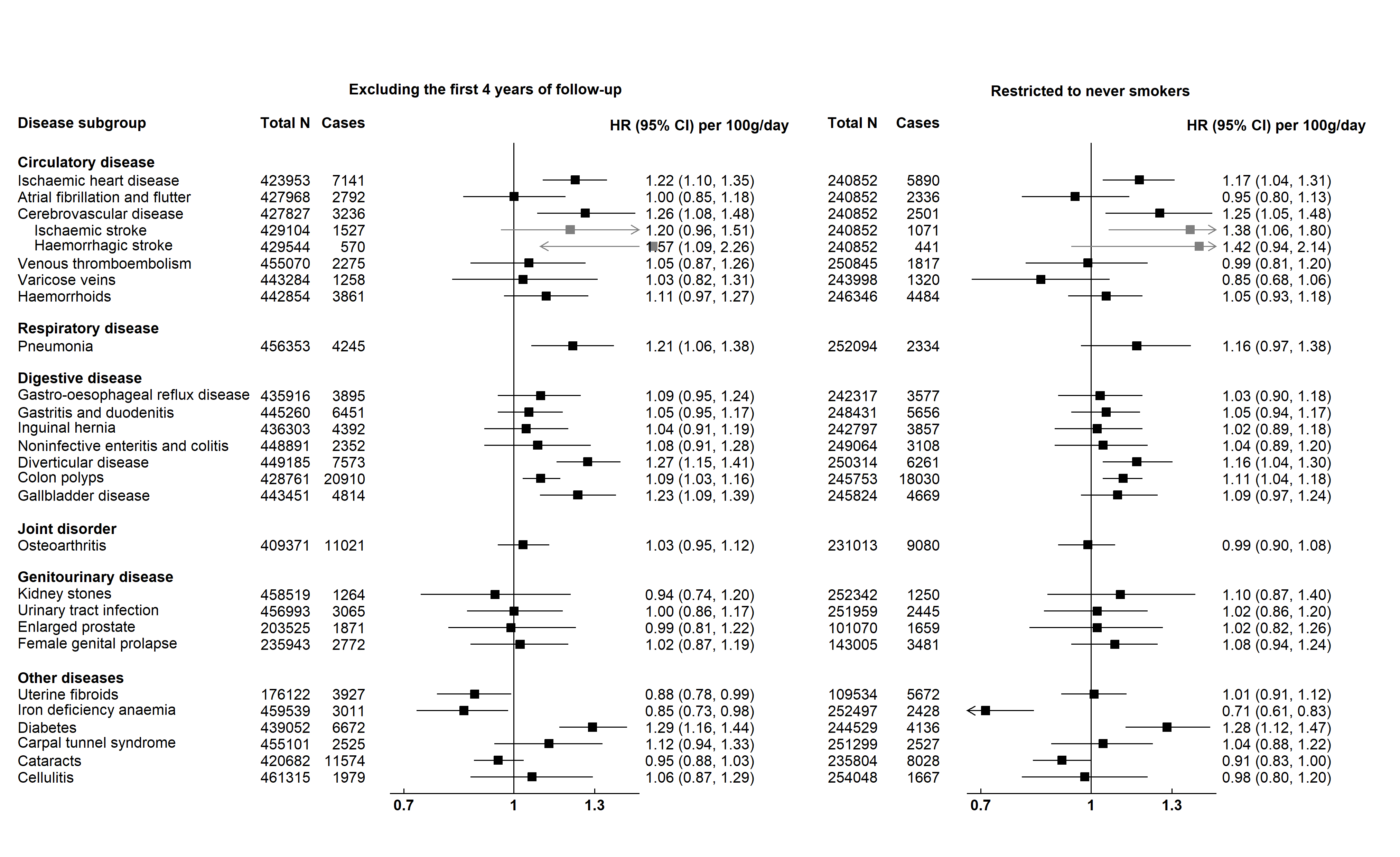


## Additional file 1: Fig. 2. **Risk of 25 common conditions by higher daily intake of total meat excluding the first 4 years of follow-up,** in never smokers.

Stratified for sex, age group and region and adjusted for age (underlying time variable), race (4 groups where possible: White, Asian or Asian British, Black or Black British, Mixed race or other, unknown), deprivation (Townsend index quintiles, unknown), qualification (College or university degree/vocational qualification, National examination at ages 17-18, National examination at age 16, other/unknown), employment (in paid employment, receiving pension, not in paid employment, unknown), smoking (never, former, current <15 cigarettes/day, current > 15 cigarettes/ day, current unknown amount of cigarettes/day, unknown), physical activity (<10 excess METs per/week, 10-<50 excess METs per/week, ≥ 50 excess METs per/week, unknown), alcohol intake (none, < 1 g/day, 1-<10 g/day, 10-<20 g/day, ≥20 g/day, unknown), total fruit and vegetable intake (< 3 servings/day, 3-< 4 servings/day, 4-< 6 servings/day, ≥ 6 servings/day, unknown), cereal fibre score (sex-specific quintiles, unknown), oily fish intake (0 times/week,<1 time/week,1 time/week,>2 times/week, unknown), non-oily fish intake (<1 time/week, 1 time/week, >2 times/week, unknown), BMI (sex-specific quintiles, unknown), in women: menopausal status (pre-, postmenopausal, unknown), HRT (never, past, current, unknown), OCP use (never, past, current, unknown), and parity (nulliparous, 1-2, ≥ 3, unknown). BMI: Body mass index, HRT: hormone replacement therapy, OCP: oral contraceptive pill.


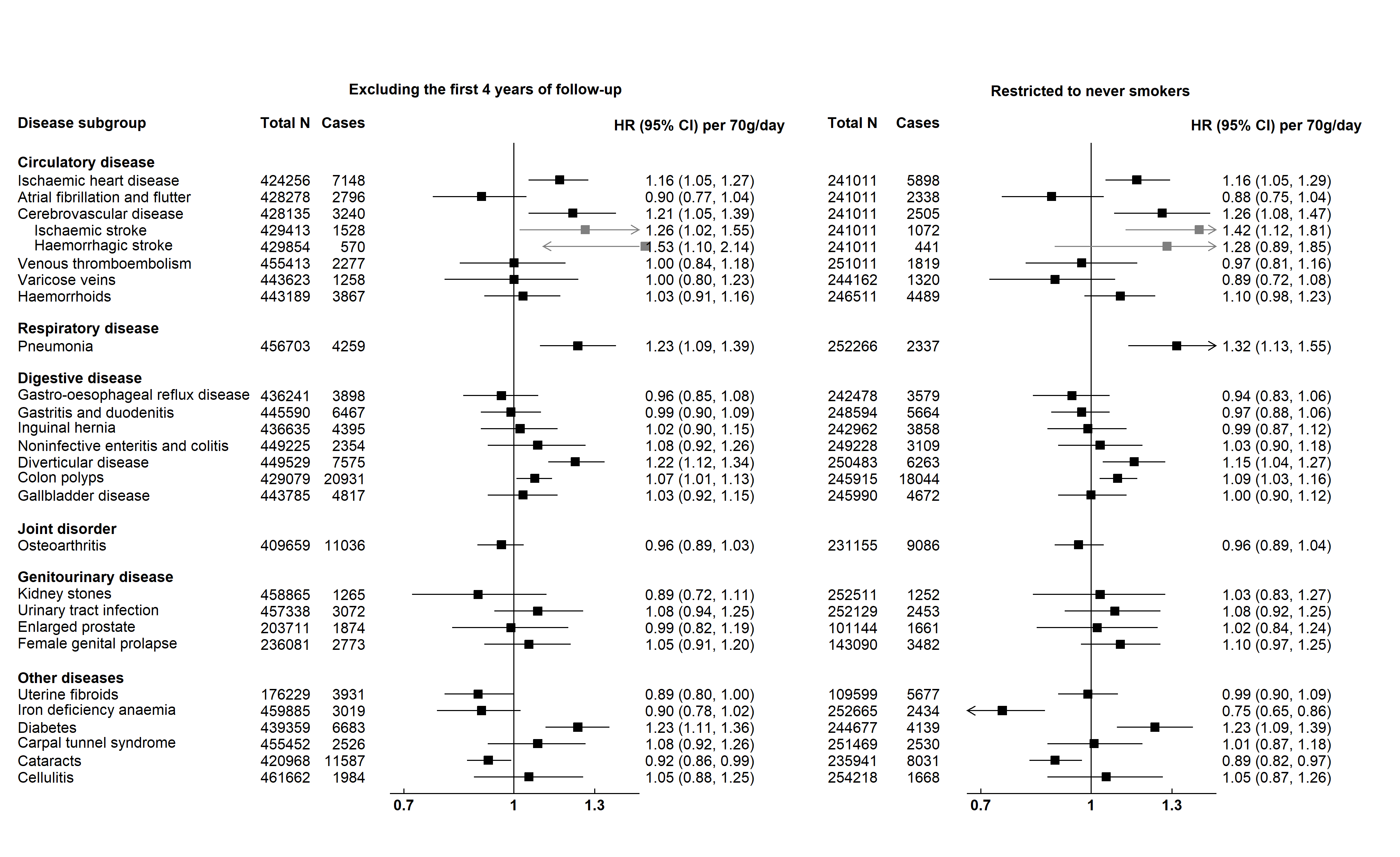


Additional file 1: Fig. 3. Risk of 25 common conditions by higher daily intake of unprocessed red and processed meat excluding the first 4 years of follow-up, in never smokers. Stratified for sex, age group and region and adjusted for age (underlying time variable), race (4 groups where possible: White, Asian or Asian British, Black or Black British, Mixed race or other, unknown), deprivation (Townsend index quintiles, unknown), qualification (College or university degree/vocational qualification, National examination at ages 17-18, National examination at age 16, other/unknown), employment (in paid employment, receiving pension, not in paid employment, unknown), smoking (never, former, current <15 cigarettes/day, current > 15 cigarettes/ day, current unknown amount of cigarettes/day, unknown), physical activity (<10 excess METs per/week, 10-<50 excess METs per/week, ≥ 50 excess METs per/week, unknown), alcohol intake (none, < 1 g/day, 1-<10 g/day, 10-<20 g/day, ≥20 g/day, unknown), total fruit and vegetable intake (< 3 servings/day, 3-< 4 servings/day, 4-< 6 servings/day, ≥ 6 servings/day, unknown), cereal fibre score (sex-specific quintiles, unknown), oily fish intake (0 times/week,<1 time/week,1 time/week,>2 times/week, unknown), non-oily fish intake (<1 time/week, 1 time/week, >2 times/week, unknown), BMI (sex-specific quintiles, unknown), in women: menopausal status (pre-, postmenopausal, unknown), HRT (never, past, current, unknown), OCP use (never, past, current, unknown), and parity (nulliparous, 1-2, ≥ 3, unknown). BMI: Body mass index, HRT: hormone replacement therapy, OCP: oral contraceptive pill.


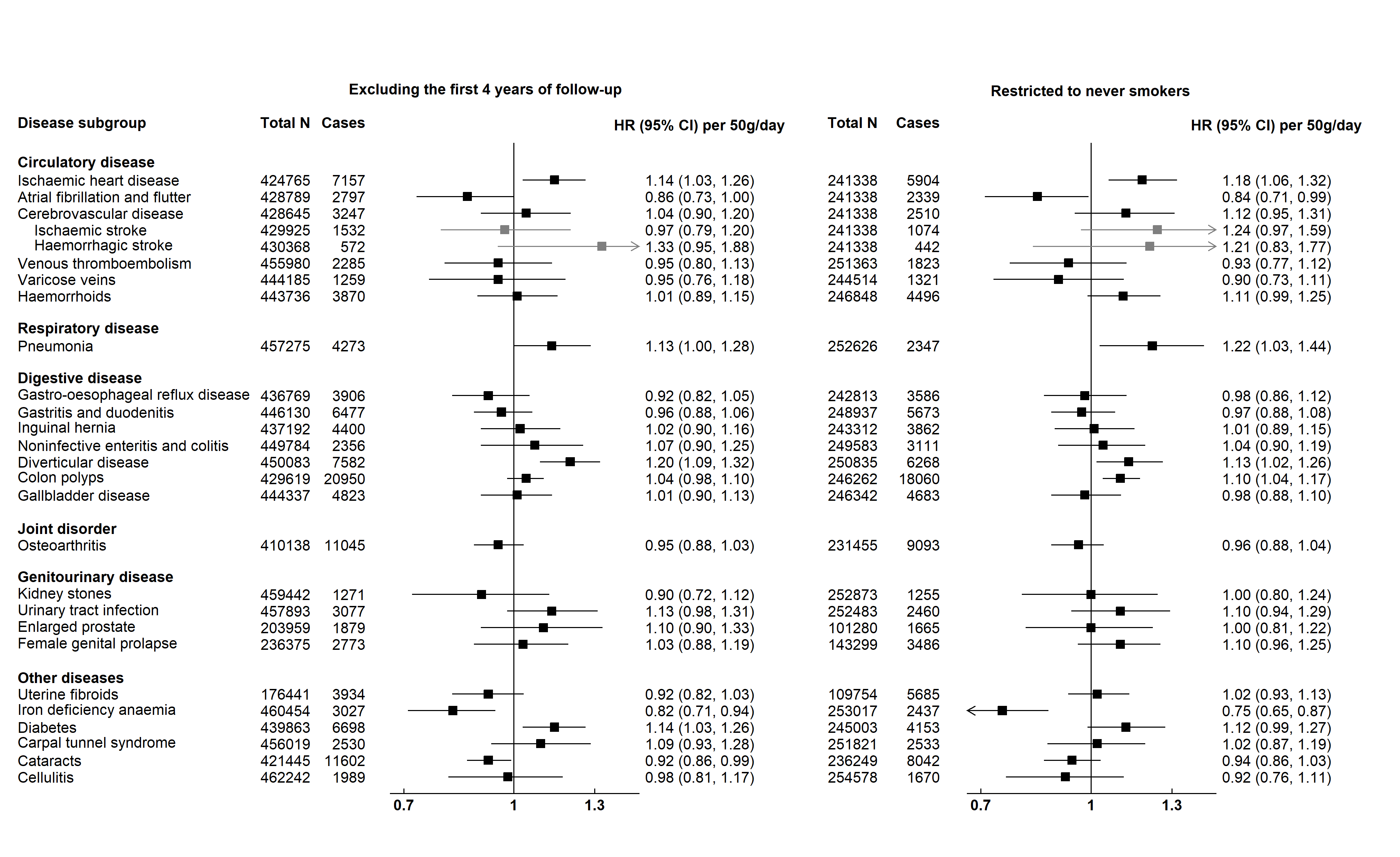


## Additional file 1: Fig. 4. Risk of 25 common conditions by higher daily intake of unprocessed red meat excluding the first 4 years of follow-up, in never smokers.

Stratified for sex, age group and region and adjusted for age (underlying time variable), race (4 groups where possible: White, Asian or Asian British, Black or Black British, Mixed race or other, unknown), deprivation (Townsend index quintiles, unknown), qualification (College or university degree/vocational qualification, National examination at ages 17-18, National examination at age 16, other/unknown), employment (in paid employment, receiving pension, not in paid employment, unknown), smoking (never, former, current <15 cigarettes/day, current > 15 cigarettes/ day, current unknown amount of cigarettes/day, unknown), physical activity (<10 excess METs per/week, 10-<50 excess METs per/week, ≥ 50 excess METs per/week, unknown), alcohol intake (none, < 1 g/day, 1-<10 g/day, 10-<20 g/day, ≥20 g/day, unknown), total fruit and vegetable intake (< 3 servings/day, 3-< 4 servings/day, 4-< 6 servings/day, ≥ 6 servings/day, unknown), cereal fibre score (sex-specific quintiles, unknown), oily fish intake (0 times/week,<1 time/week,1 time/week,>2 times/week, unknown), non-oily fish intake (<1 time/week, 1 time/week, >2 times/week, unknown), BMI (sex-specific quintiles, unknown), in women: menopausal status (pre-, postmenopausal, unknown), HRT (never, past, current, unknown), OCP use (never, past, current, unknown), and parity (nulliparous, 1-2, ≥ 3, unknown). BMI: Body mass index, HRT: hormone replacement therapy, OCP: oral contraceptive pill.


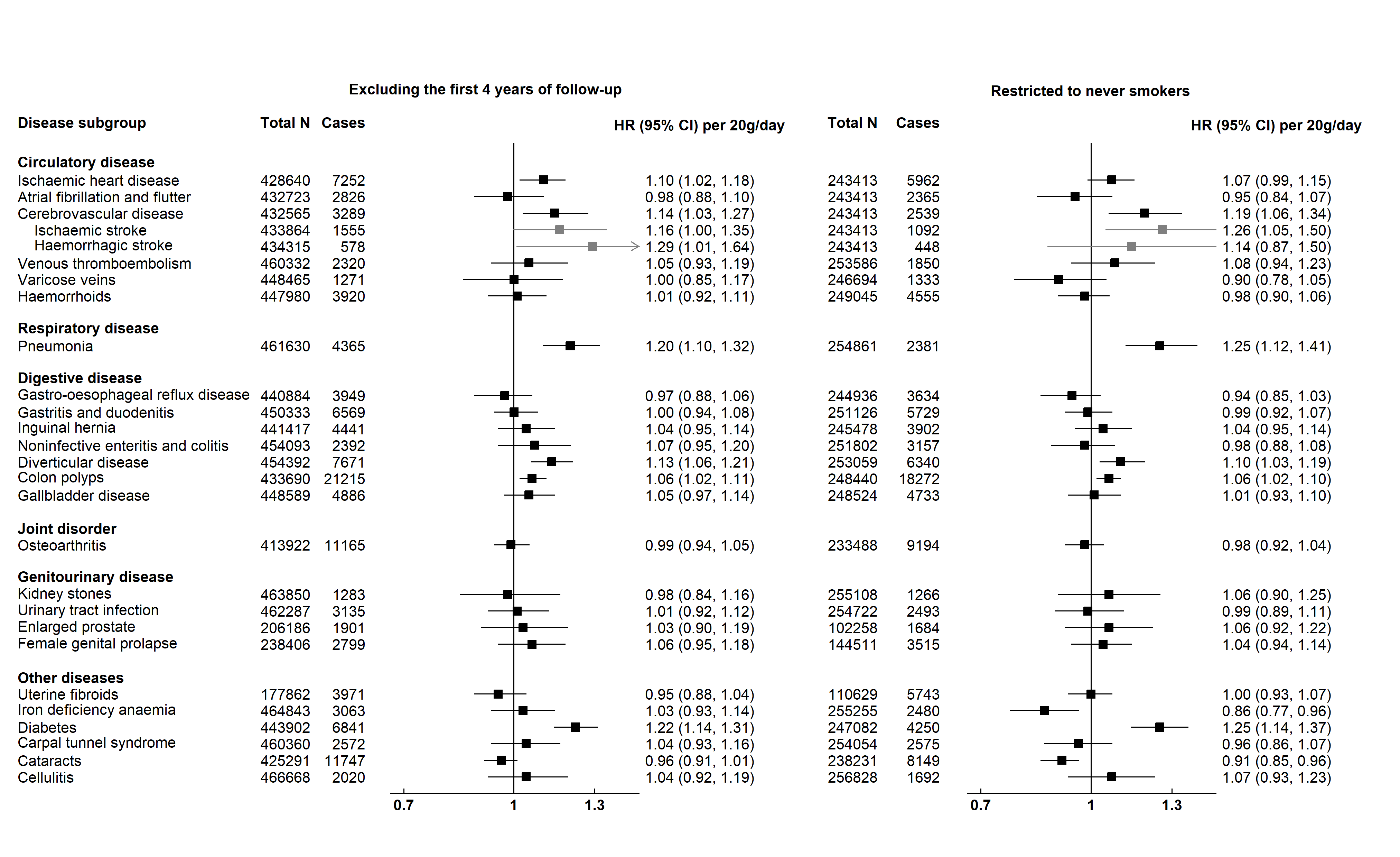


Additional file 1: Fig. 5. Risk of 25 common conditions by higher daily intake of processed meat excluding the first 4 years of follow-up, in never smokers.

Stratified for sex, age group and region and adjusted for age (underlying time variable), race (4 groups where possible: White, Asian or Asian British, Black or Black British, Mixed race or other, unknown), deprivation (Townsend index quintiles, unknown), qualification (College or university degree/vocational qualification, National examination at ages 17-18, National examination at age 16, other/unknown), employment (in paid employment, receiving pension, not in paid employment, unknown), smoking (never, former, current <15 cigarettes/day, current > 15 cigarettes/ day, current unknown amount of cigarettes/day, unknown), physical activity (<10 excess METs per/week, 10-<50 excess METs per/week, ≥ 50 excess METs per/week, unknown), alcohol intake (none, < 1 g/day, 1-<10 g/day, 10-<20 g/day, ≥20 g/day, unknown), total fruit and vegetable intake (< 3 servings/day, 3-< 4 servings/day, 4-< 6 servings/day, ≥ 6 servings/day, unknown), cereal fibre score (sex-specific quintiles, unknown), oily fish intake (0 times/week,<1 time/week,1 time/week,>2 times/week, unknown), non-oily fish intake (<1 time/week, 1 time/week, >2 times/week, unknown), BMI (sex-specific quintiles, unknown), in women: menopausal status (pre-, postmenopausal, unknown), HRT (never, past, current, unknown), OCP use (never, past, current, unknown), and parity (nulliparous, 1-2, ≥ 3, unknown). BMI: Body mass index, HRT: hormone replacement therapy, OCP: oral contraceptive pill.


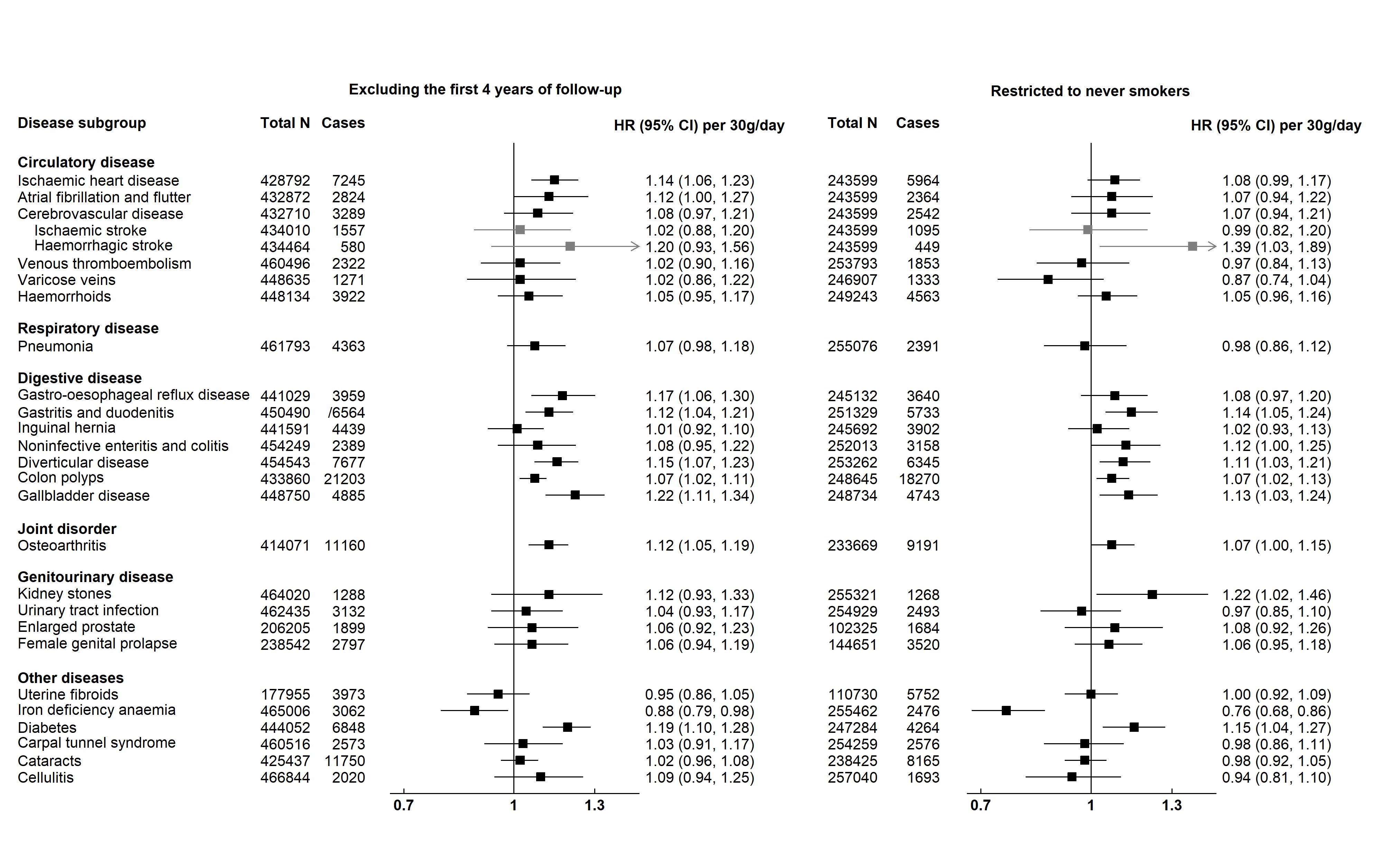


## Additional file 1: Fig. 6. Risk of 25 common conditions by higher daily intake of poultry meat excluding the first 4 years of follow-up, in never smokers.

Stratified for sex, age group and region and adjusted for age (underlying time variable), race (4 groups where possible: White, Asian or Asian British, Black or Black British, Mixed race or other, unknown), deprivation (Townsend index quintiles, unknown), qualification (College or university degree/vocational qualification, National examination at ages 17-18, National examination at age 16, other/unknown), employment (in paid employment, receiving pension, not in paid employment, unknown), smoking (never, former, current <15 cigarettes/day, current > 15 cigarettes/ day, current unknown amount of cigarettes/day, unknown), physical activity (<10 excess METs per/week, 10-<50 excess METs per/week, ≥ 50 excess METs per/week, unknown), alcohol intake (none, < 1 g/day, 1-<10 g/day, 10-<20 g/day, ≥20 g/day, unknown), total fruit and vegetable intake (< 3 servings/day, 3-< 4 servings/day, 4-< 6 servings/day, ≥ 6 servings/day, unknown), cereal fibre score (sex-specific quintiles, unknown), oily fish intake (0 times/week,<1 time/week,1 time/week,>2 times/week, unknown), non-oily fish intake (<1 time/week, 1 time/week, >2 times/week, unknown), BMI (sex-specific quintiles, unknown), in women: menopausal status (pre-, postmenopausal, unknown), HRT (never, past, current, unknown), OCP use (never, past, current, unknown), and parity (nulliparous, 1-2, ≥ 3, unknown). BMI: Body mass index, HRT: hormone replacement therapy, OCP: oral contraceptive pill.


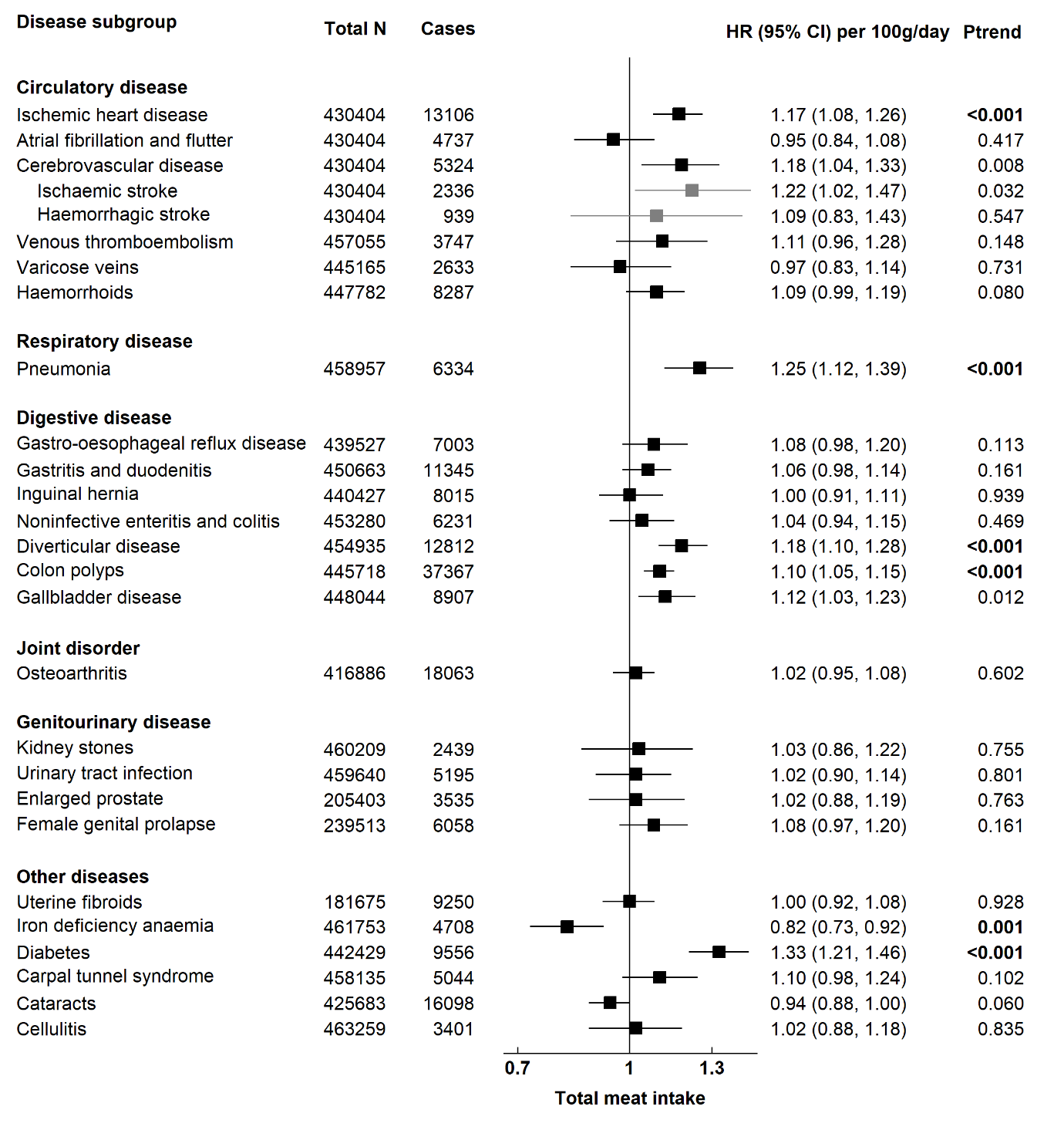


## Additional file 1: Fig. 7. Risk of 25 common conditions per 100 g/day higher daily intake of total meat.

Stratified for sex, age group and region and adjusted for age (underlying time variable), race (4 groups where possible: White, Asian or Asian British, Black or Black British, Mixed race or other, unknown), deprivation (Townsend index quintiles, unknown), qualification (College or university degree/vocational qualification, National examination at ages 17-18, National examination at age 16, other/unknown), employment (in paid employment, receiving pension, not in paid employment, unknown), smoking (never, former, current <15 cigarettes/day, current > 15 cigarettes/ day, current unknown amount of cigarettes/day, unknown), physical activity (<10 excess METs per/week, 10-<50 excess METs per/week, ≥ 50 excess METs per/week, unknown), alcohol intake (none, < 1 g/day, 1-<10 g/day, 10-<20 g/day, ≥20 g/day, unknown), total fruit and vegetable intake (< 3 servings/day, 3-< 4 servings/day, 4-< 6 servings/day, ≥ 6 servings/day, unknown), cereal fibre score (sex-specific quintiles, unknown), oily fish intake (0 times/week,<1 time/week,1 time/week,>2 times/week, unknown), non-oily fish intake (<1 time/week, 1 time/week, >2 times/week, unknown), BMI (sex-specific quintiles, unknown), in women: menopausal status (pre-, postmenopausal, unknown), HRT (never, past, current, unknown), OCP use (never, past, current, unknown), and parity (nulliparous, 1-2, ≥ 3, unknown). BMI: Body mass index, HRT: hormone replacement therapy, OCP: oral contraceptive pill. P trend in bold: P value robust to Bonferroni correction (*P*<0.002).
